# Gene regulatory networks involved in activation of Notch signaling by AGEs in the pathogenesis of diabetic kidney disease

**DOI:** 10.1371/journal.pone.0335768

**Published:** 2026-01-02

**Authors:** Somorita Baishya, Adyasha Sarangi, Pramod R. Somvanshi, Anil Kumar Pasupulati

**Affiliations:** 1 Department of Biochemistry, University of Hyderabad, Hyderabad, Telangana, India; 2 Department of Biotechnology, School of Sciences, Woxsen University, Hyderabad, Telangana, India,; 3 Department of Systems and Computational Biology, University of Hyderabad, Hyderabad, Telangana, India; 4 Division of Renal Medicine, Department of Medicine, Brigham and Women’s Hospital, Harvard Medical School, Boston, Massachusetts, United States of America; Brigham and Women’s Hospital Division of Cardiovascular Medicine, UNITED STATES OF AMERICA

## Abstract

Diabetic kidney disease (DKD) is a progressive disease characterized by early events such as podocyte injury followed by glomerulosclerosis, varying degrees of proteinuria, decreased glomerular filtration rate, and eventual organ failure. Podocytes, essential for glomerular permselectivity, are targets for an array of noxious stimuli in diabetes. Notch signaling, critical for podocyte differentiation during nephrogenesis, reactivates in mature podocytes, as evidenced in DKD patients and animal models. Notch reactivation in adult podocytes implicated in de-differentiation and apoptosis. Although elevated advanced glycation end-products (AGEs), heterogeneous molecules derived from non-enzymatic glycation, were paralleled with Notch reactivation in podocytes, the precise mechanism remains unknown. This study identified a condensed network of 58 genes modulated by AGEs. These genes non-canonically reactivate Notch by suppressing the PI3K-AKT pathway and activating NF-kB signaling, affecting podocyte biology and function. The study provides novel information about reno-toxic events and new therapeutic targets to prevent podocyte injury and kidney dysfunction.

## Introduction

According to WHO, approximately 422 million individuals globally have diabetes, with the majority residing in low- and middle-income nations. The disease is directly responsible for 1.5 million fatalities annually (WHO, 2024). Over the past few decades, there has been a steady rise in both the number of cases and the incidence of diabetes [1]. Persistent hyperglycemia is a hallmark of diabetes mellitus (DM), which can be caused by insulin resistance (type II) or an absolute or relative insulin shortage (type I) [2]. DM is one of the leading causes of the development of kidney problems; around 30% of people with type I and 10% to 40% of those with type II DM will eventually experience renal failure [3]. The longer a patient suffers from DM, the greater their risk of developing secondary complications of diabetes, including diabetic kidney disease (DKD). DKD is widespread across the globe, adversely affecting human health with substantial economic burdens on human society [4]. DKD is a significant contributor to chronic kidney disease (CKD) [5]. DKD is presented with functional alterations such as decreased glomerular filtration rate (GFR) and varying degrees of proteinuria [6]. It also leads to altered metabolic, inflammatory, haemodynamic signalling pathways along with several molecular mediators that creates a feedback loop in promoting general kidney damage [7]**.** Structural alterations that prevail in DKD include glomerulosclerosis, thickening of the glomerular basement membrane, and podocytopathy [8]. Podocytes, the visceral cells of the glomerulus, provide epithelial coverage to the capillaries, thereby playing an instrumental role in the permselectivity and preventing protein loss into the urine. Podocytes are terminally differentiated cells with very limited proliferative potential, therefore an absolute podocyte number and integrity are critical factors in maintaining normal renal function [6].

Podocytes are targets of intensive insults in various clinical conditions [9]. Podocyte injury is considered an early insult in the settings of DKD, and injury of podocytes parallels with proteinuria [10]. For instance, the transition from epithelial to mesenchymal phenotype is one of the insults to podocytes in the settings of DKD [11,12]. This phenomenon of de-differentiation of podocytes is an outcome of the reactivation of Notch signaling in the adult podocytes [13]. Notch signaling is crucial for fate determination during embryological development; particularly, it is warranted for the differentiation of progenitors during nephrogenesis [12]. This juxta cellular signaling is activated when one cell’s ligand (Delta or Jagged) interacts with the adjacent cell’s Notch receptor (Notch 1–4). Notch ligand and receptor interaction triggers proteolytic cleavage of the Notch intracellular domain (NICD), which could trigger the expression of target genes upon translocation to the nucleus. [11]. However, in terminally differentiated podocytes Notch signaling is generally minimal and inactive [14]. Accumulated evidence suggests that reactivation of Notch signaling leads to the loss of terminal differentiation markers and forces podocytes to re-enter the cell cycle, thereby creating deleterious effects [12,14–16] while prevention of Notch reactivation showed nephroprotective effects [11,17,18].

Chronic hyperglycemia leads to the accumulation of advanced glycation end-products (AGEs) in various tissues, contributing to microvascular complications in the eye lens, retina, and kidney, as well as macrovascular complications in the arteries of the heart, brain, and lower limbs. These pathogenic processes are strongly implicated in the development of cataracts, diabetic retinopathy, diabetic kidney disease (DKD), cardiovascular disease, cerebrovascular disease, and peripheral vascular disease [19–23]

Several noxious stimuli that prevail in diabetes settings were shown to induce reactivation of Notch signaling in adult podocytes. They include glucose, growth hormone, and advanced glycation end-products (AGEs) [11,24–26]. During chronic hyperglycemic conditions, glucose reacts with a free-amino group of amino acids and eventually forms glycation adducts on client proteins by a series of non-enzymatic reactions [27,28]. Such chronic exposure leads to the accumulation of AGEs in various tissues, contributing to microvascular complications in the eye lens, retina, and kidney as well as macrovascular complications in the arteries of the heart, brain, and lower limbs. These processes are implicated in the pathogenesis of several complications, including cataracts, retinopathy, DKD, cardiovascular disease, cerebrovascular disease, and peripheral vascular diseases [29–31]. AGEs also interact with lipids and nucleic acids, elicit cellular and molecular changes, and orchestrate the end-organ pathology in diabetes and aging settings. Kidneys eliminate AGEs circulating in the bloodstream through glomerular filtration. However, elevated levels of AGEs due to hyperglycemia result in insufficient removal, increasing their concentration. Excess AGEs in the circulation or accumulated AGEs in the kidney increases the risk of renal dysfunction [11,32–37]. Upon binding to their receptors (RAGEs), AGEs trigger anomalous signaling cascades [38]. Recent study have also found upregulation of RAGE expression in SGLT2-mediated glucose uptake in proximal tubular cells, being involved in the tubulointerstitial [39]. However, the precise mechanism of Notch activation by these cytotoxic AGE molecules is largely unknown.

Generally, for eliciting a response, proteins work in bio-molecular networks utilizing multiple feedback loops to accomplish a signal transduction. The topology and kinetics of the network dictate its output response [40,41]. Hence, we took a holistic approach to understanding the Notch reactivation mechanism. We used dataset generated by high throughput technologies to get insights into the molecular and cellular players in the reactivation. This panoramic view generated a condensed network of 58 genes that predicted that AGEs fostered by chronic hyperglycemia suppress PI3K-AKT pathway, activate NF-kB signaling and elicit inflammatory responses which in turn reactivates Notch signaling Reactivation of Notch also suppresses PI3K-AKT signaling. This induces apoptosis in the terminally differentiated podocytes. These findings can be exploited to develop treatments that can stop podocyte injury and can cure Diabetic nephropathy (DN).

## Materials and methods

### Data acquisition

The gene expression profile for microarray data was collected from Gene ExpressionOmnibus (GEO) database through the keywords “DKD” or “DN”. We downloaded and analyzed the expression profile of GSE30122 from GPL571 platform, that had 19 DN and 50 Control. GSE30122 contained both glomerular and tubular specimens. Raw data for the dataset was contained in zipped. TAR files were downloaded and untarred manually to obtain individual.CEL files. “GEOquery” (v2.70.0) [42] package was used for accessing GEO SOFT files for each dataset..CEL files for the dataset was parsed to R studio using “ReadAffy” of “Affy” (v 1.80.0) package [43]. To confirm homogeneity across datasets and evaluation of their quality we performed background correcting, data normalization, batch effect removal using “rma” from “Affy” (v 1.80.0) package and “ComBat” from “sva” package [44]. Subsequently, probe annotation was performed and probes with multiple genes were excluded. Genes mapping to multiple probes were calculated as average.

### Identification of differentially expressed genes

Differentially expressed genes (DEGs) from the dataset were identified using “limma” package (v 3.58.1) of R software with |log2FC| > 1 and p < 0.001 considered significant [45]. Volcano plot for the DEGs were visualized using “ggplot2” package (v 3.4.4).

### Functional enrichment analysis

To understand the significant pathways involved in DN and to identify the genes involved with Notch signaling and AGE-RAGE signaling we performed their Gene Ontology (GO) and Kyoto Encyclopedia of Genes and Genomes (KEGG) pathway analysis of the DEGs. The GO resource tool provides annotation for molecular function (MF), cellular component (CC), and biological process (BP) of genes to facilitate the functional aspects of genes during computational analysis. KEGG analysis was performed to assess the functional aspect of the genes. “Clusterprofiler” was employed to give insights into the enrichment analysis of DEGs [46]. R package “org.Hs.e.g.,db” was used for conversion between gene IDs. Cluepedia app of Cytoscape was used to generate and explore the expression, activation, and inhibitory relationship between the gene interaction network [47,48]. STRING Action File in Cluepedia panel was used as source for action query and the interactions were set to different colours to indicate the action. The action were based on a statistical method called Kappa scoring, which range from 0 to 1, but can also be customized.

### Gene set enrichment analysis

Gene set enrichment analysis (GSEA) was performed using “clusterProfiler” (v 4.10.0) to determine the pathways that were enriched in the dataset [46]. This analysis uses set of predefined genes to determine whether the pooled genes represent specific well-defined biological states and display coherent expression. The top “HALLMARK” pathway terms were depicted based on Net Enrichment Score (NES), p, and gene ratio. P was set at 0.05, minimum and maximum gene set size was set at 25 and 500 respectively and Benjamini–Hochberg (BH) method was applied to adjust the p for the false discovery rate (FDR) in our study. The tests were regarded as significant with an adjusted p threshold of 0.05 and gene count ≥2. The gene set database was downloaded from Human MSigDB Collections.

### PPI network creation and identification of subnetworks

Protein-protein interaction (PPI) network for the genes involved with the top 5 pathways along with Notch signaling and AGE-RAGE signaling was generated using STRING app integrated with Cytoscape 3.10.1. confidence level 0.90 [48,49]. The highly interconnected subnetworks (clusters/ hubs) of the merged network were identified using MCODE plugin of Cytoscape software, where degree cutoff = 2, node score cutoff = 0.2, and k-core = 2 was considered as filtering criteria [50].

### Analysis of topological parameters

Network Analyzer plugin of Cytoscape was utilized to check the central measures of the nodes [51]. Among the various central measures provided by Network Analyzer, we based our study on the degree, betweenness centrality (BC) and clustering coefficient. Degree of a node indicates the number of node connections a particular node has, whereas BC indicates the frequency with which a node is located on the shortest path linking other nodes and clustering coefficient reflects the strength of degree. We discarded nodes having clustering co-efficient > 0.5 in order to focus on a tight network. Thus, analysing topological properties will give insights into the functioning and essentiality of the nodes at the systemic level [52]. To understand more deeply correlation between the genes a correlation plot was generated using “corplot” of R studio.

### Validation with RNA-Seq data

To independently validate the transcriptional changes identified from the microarray dataset GSE30122, we performed a complementary analysis of publicly available RNA-seq data (GSE299230), which profiles gene expression in human kidney tissue under DN–relevant conditions. Raw FPKM values were retrieved from GEO and processed in R studio. Genes with FPKM > 1 in at least two samples were retained to remove low-abundance transcripts, and expression values were log2-transformed.

Differential expression analysis was performed using the limma-voom pipeline, with statistical thresholds set at adjusted p-value < 0.01 (Benjamini–Hochberg correction) and absolute log2 fold-change > 0. Overlapping genes between RNA-seq DEGs and the 167 AGE-associated genes from the microarray dataset were identified using base R functions.

Venn diagram of the genes common between the 2 platforms was constructed using https://bioinformatics.psb.ugent.be/webtools/Venn/.

### Validation of existing dataset with regional transcriptomic datasets

The significant driver genes identified based on centrality and statistical measures were cross-referenced to the publicaly available Kidney Precision Medicine Project (KPMP) database (https://atlas.kpmp.org/). We checked the regional transcriptomic expression of the key genes to find consistency and relevance of our findings with regards to AGE-induced DN. A violin plot was plotted to compare the logFC distribution of the expressions from the existing and the independent dataset and scatter plot with connected lines was plotted to using “ggplot2” package (v 3.4.4).

### Statistical analysis

All statistical analysis were performed on R software (version 4.3.2).

## Results

### 1620 differentially expressed genes (DEGs) identified between control and DN patients

**Fig 1:**
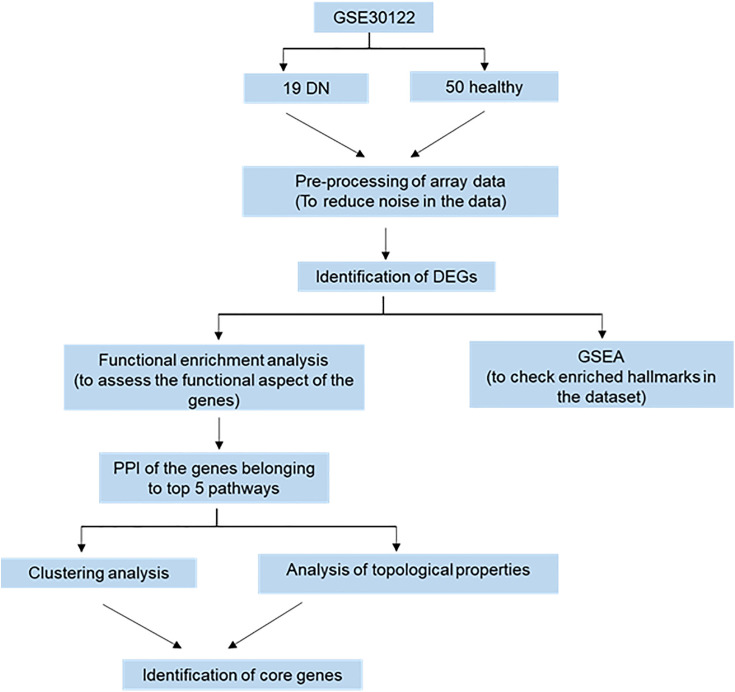
Scheme of study workflow. GSE30122 was acquired fromGEO Database. Post pre-processing data in R studio, differentially expressed genes (DEGs) were identified using “limma”. Gene set enrichment analysis (GSEA) and functional enrichment analysis was performed using “ClusterProfiler” to identify the hallmarks and pathways enriched in pathogenesis of DN. PPI network was generated in Cytoscape with the genes involved in top 5 pathways at 0.90 confidence level. Clustering analysis and analysis of topological parameters were performed to identify the core gene network using MCODE and Network Analyzer respectively. Cluepedia was used for visualising the genes and their pathway association.

**Fig 2:**
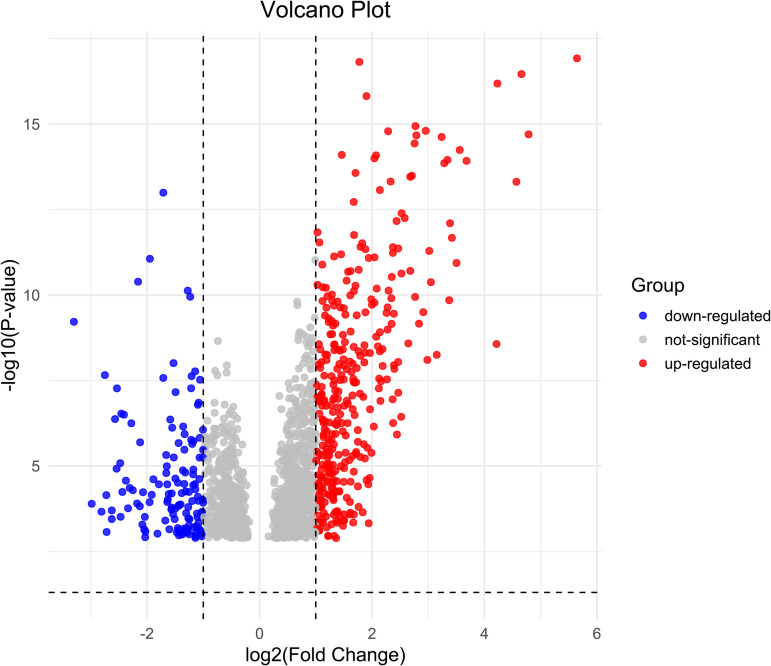
1620 DEGs identified. DEGs from the dataset were identified using “limma” package of R software with |log2FC| > 0 and p < 0.01 considered significant. Volcano plot for the DEGs were visualized using “ggplot2” package. 960 genes denoted in red were upregulated and 660 gene depicted in blue were downregulated and the non-significant genes are denoted in grey (|log2FC| > 0 and p < 0.01).

Fig 1 depicts the workflow of the present study. Post pre-processing and annotating GSE30122 we performed differential gene expression analysis with |log2FC| > 0 and p < 0.01. We identified 1620 genes were differentially expressed between healthy (control) and DN patients, which included 960 upregulated and 660 downregulated (Supplementary table 1 in S1 File). The DEGs are represented as a volcano plot in Fig 2.

### PI3K-Akt signaling pathway, inflammatory pathways and epithelial mesenchymal transition processes are most enriched in DN condition

**Fig 3:**
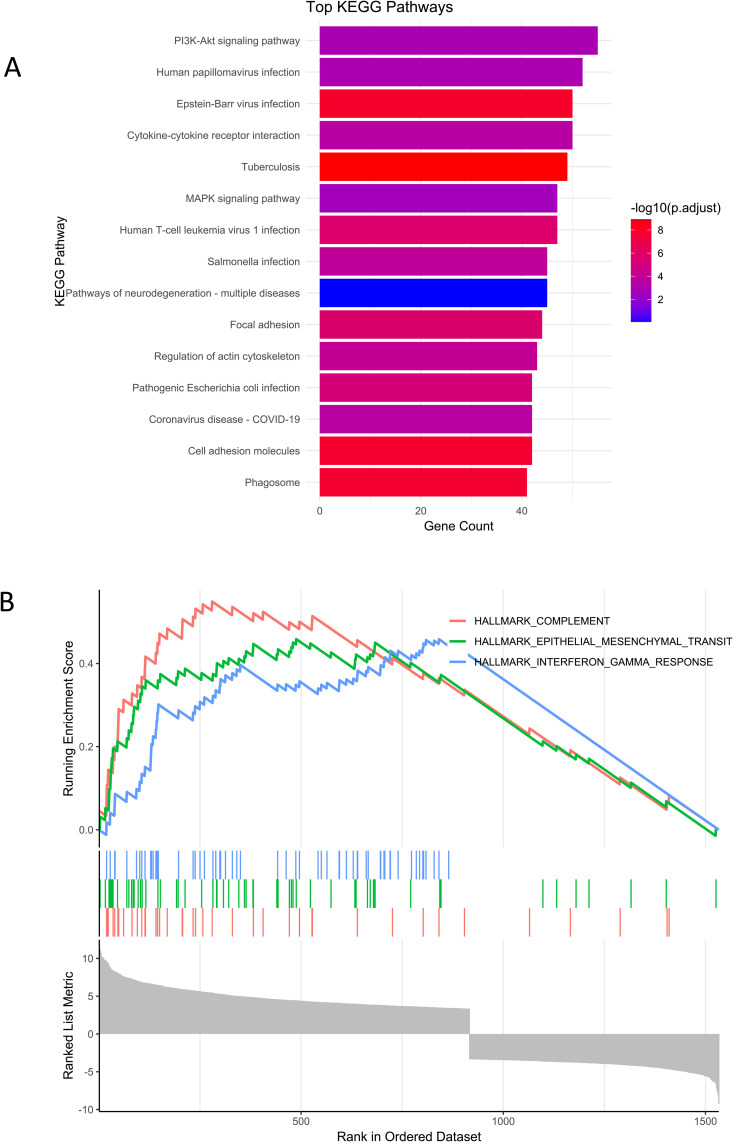
PI3K-Akt signaling pathway, inflammatory pathways and epithelial mesenchymal transition processes are most enriched in DN condition. A. KEGG pathway analysis shows the top 15 enriched pathways are represented as bar plot. PI3K-AKT pathway, Cytokine-cytokine receptor interaction, MAPK signaling pathway, focal adhesion and regulation of actin cytoskeleton are the most enriched pathways in DN condition. B. Gene set enrichment analysis (GSEA) shows the complement, interferon gamma response, and epithelial mesenchymal transition hallmarks upregulated in DN pathogenesis based on NES, meaning that inflammation and fibrosis- related pathways are upregulated in DN condition.

We tried to dive into the pathways that are most enriched in diabetic condition and identify genes associated with Notch signaling and AGE-RAGE signaling (Supplementary table 2 in S1 File). KEGG pathway analysis revealed PI3K-AKT pathway is the most enriched pathway in DN condition. 55 genes were involved in this pathway. Apart from this Cytokine-cytokine receptor interaction, MAPK signaling pathway, Focal adhesion, and Regulation of actin cytoskeleton pathways were the top 5 pathways that were enriched in DN condition (Fig 3A, Table 1). We also found 21 genes were involved in AGE-RAGE signaling and 3 genes were involved with Notch signaling (Supplementary table 2 in S1 File). We also studied the hallmarks enriched across the dataset using GSEA and found hallmarks for COMPLEMENT, INTERFERON GAMMA RESPONSE, and EPITHELIAL MESENCHYMAL TRANSITION were the top 3 hallmarks descriptions based on NES (Fig 3B, Supplementary table 3 in S1 File). KEGG pathway analysis helped in identifying 167 unique genes with which we carried on our further analysis (Table 2).

**Table 1:** Top 5 pathways and their genes from KEGG analysis.

ID	Description	pvalue	p.adjust	Count	qvalue
hsa04151 Gene ID	PI3K-Akt signaling pathway	0.000219	0.001299	55	0.000874
	COL1A2/TNC/COL6A3/THBS2/FN1/FGF9/IL7R/CSF1R/LAMC2/TLR2/SYK/VWF/ITGA4/LPAR1/EGF/COMP/NGF/GHR/SPP1/ITGA2/IL7/PDGFRA/YWHAH/LAMA2/PTEN/RXRA/MAGI2/VEGFA/HGF/LAMC1/CCND2/FLT1/JAK1/MYC/PHLPP1/COL4A1/PIK3CB/ITGB7/COL9A1/EFNA4/MET/FGF7/CCNE1/LAMB3/IFNAR2/EFNA1/LAMB1/FLT3LG/RPS6/TCL1B/IKBKG/IL2RB/CDK4/COL6A2/FGF4				
hsa04060 Gene ID	Cytokine-cytokine receptor interaction	3.35E-05	0.000288	50	0.000194
	CXCL6/IL10RA/CCR2/ACKR4/FAS/IL7R/CSF1R/CCL19/CCR7/IL33/CCL5/CCL2/LTB/IFNGR2/TNFSF15/BMP7/CXCR4/CX3CR1/TNFRSF1B/NGF/CD27/GHR/TNFSF10/IL7/CXCL1/CCR5/CCR6/IL32/CXCR6/TNFRSF17/PF4V1/CTF1/TNFRSF1A/XCL1/IL18/BMP5/TNFRSF11B/CXCL12/IL21R/IFNAR2/INHBA/ACVR2A/IL2RB/CD40/CXCL8/CCL18/IFNGR1/IL16/CCL21/IL37				
hsa04010 Gene ID	MAPK signaling pathway	0.000307	0.00176	47	0.001184
	FGF9/FAS/CSF1R/MYD88/PLA2G4A/GADD45B/EGF/RAC2/PRKCB/CASP3/NGF/RASA2/MAP4K1/PDGFRA/MAPK13/CACNB2/VEGFA/HGF/RPS6KA3/GNA12/FLT1/MYC/TNFRSF1A/GADD45G/CD14/MAPK8/HSPA1L/EFNA4/IRAK1/NLK/DUSP1/MET/FGF7/RPS6KA1/CACNA2D3/GADD45A/MAPK10/ELK1/EFNA1/FLT3LG/PAK2/IKBKG/CDC25B/DDIT3/MAPK8IP2/CACNA1D/FGF4				
hsa04510 Gene ID	Focal adhesion	8.90E-08	2.43E-06	44	1.63E-06
	COL1A2/TNC/COL6A3/THBS2/FN1/ACTN1/LAMC2/VWF/ITGA4/EGF/COMP/RAC2/PRKCB/BIRC3/SPP1/ITGA2/PDGFRA/PIP5K1C/LAMA2/PTEN/ACTB/VAV1/VEGFA/HGF/LAMC1/CCND2/FLT1/COL4A1/PIK3CB/ITGB7/COL9A1/MAPK8/MET/LAMB3/MAPK10/ELK1/PAK6/LAMB1/MYL10/PAK2/PAK4/MYL12B/COL6A2/CTNNB1				
hsa04810 Gene ID	Regulation of actin cytoskeleton	8.04E-06	8.48E-05	43	5.70E-05
	ITGB2/FN1/FGF9/ACTN1/ITGAM/BAIAP2/C7/PFN1/ENAH/ARPC1B/ITGA4/LPAR1/MSN/EGF/RAC2/CXCR4/GNA13/ITGA2/PDGFRA/PIP5K1C/ACTB/VAV1/GNA12/PIK3CB/ITGB7/FGF7/SSH1/ITGAX/ITGAL/ARPC3/CXCL12/TMSB4Y/PAK6/MYL10/PAK2/ACTR3/PAK4/MYL12B/SCIN/RDX/KNG1/C6/FGF4				

**Table 2:** 167 genes identified from KEGG pathway analysis that are closely associated with DN pathogenesis along with their module and topological properties.

SYMBOL	log2FC	p	Cluster	Clustering co-efficient	Degree	Betweeness Centrality
ACKR4	1.18	6.12E-11	Unclustered	0.833333	4	1.06E-05
ACTB	0.78	4.87E-06	Cluster 3	0.066667	15	0.161055
ACTN1	1.44	2.30E-10	Unclustered	1	2	0
ACTR3	0.79	4.87E-04	Cluster 3	1	3	0
ACVR2A	0.52	5.18E-04	Unclustered	0	3	1
ARPC1B	1.67	2.81E-08	Cluster 3	1	3	0
ARPC3	1.38	2.30E-04	Cluster 3	1	3	0
BAIAP2	−0.74	2.21E-09	Unclustered	0.333333	3	0.006713
BIRC3	1.44	3.05E-07	Cluster 4	0.4	6	0.052912
BMP5	0.47	1.20E-04	Unclustered	0	1	0
BMP7	−1.08	1.41E-07	Unclustered	0	1	0
C6	0.76	0.001029	Unclustered	0	1	0
C7	2.65	2.59E-09	Unclustered	0	1	0
CACNA1D	0.46	0.001226	Unclustered	0	2	0.031492
CACNA2D3	0.57	1.91E-04	Unclustered	0	1	0
CACNB2	−0.91	1.27E-05	Unclustered	0	2	0.015873
CASP3	0.97	2.07E-07	Unclustered	0	7	0.140125
CCL18	0.51	8.92E-04	Unclustered	0	1	0
CCL19	2.84	6.86E-10	Cluster 1	0.5	12	0.006027
CCL2	1.86	9.33E-09	Cluster 1	0.6	10	0.028701
CCL21	0.64	0.001182	Cluster 1	0.575758	12	0.004554
CCL5	2.47	9.09E-09	Cluster 1	0.45	16	0.012644
CCND2	0.80	2.56E-05	Unclustered	0	1	0
CCNE1	0.48	1.40E-04	Unclustered	1	2	0
CCR2	1.45	6.43E-12	Cluster 1	0.781818	11	6.79E-04
CCR5	0.83	1.25E-05	Cluster 1	0.564103	13	0.019444
CCR6	0.48	1.45E-05	Unclustered	0.761905	7	2.56E-04
CCR7	1.06	2.74E-09	Cluster 1	0.487179	13	0.044848
CD14	1.31	7.38E-05	Unclustered	0	1	0
CD27	1.14	5.49E-07	Unclustered	0	2	0.018803
CD40	0.31	6.30E-04	Cluster 4	0.4	6	0.036619
CDC25B	0.75	5.42E-04	Unclustered	0	2	0.015873
CDK4	0.49	6.21E-04	Unclustered	0.333333	3	0.015873
COL18A1	3.34		Unclustered	0	1	0
COL1A2	2.52	1.12E-14	Unclustered	0.5	5	0.023492
COL3A1	1.42	3.64E-07	Unclustered	0.666667	4	0.00781
COL4A1	0.48	5.46E-05	Unclustered	0	0	0
COL6A2	3.39	8.19E-04	Unclustered	1	2	0
COL6A3	−0.43	7.94E-13	Unclustered	0.666667	3	6.35E-05
COL9A1	1.35	7.10E-05	Unclustered	0	0	0
COMP	1.35	6.10E-08	Unclustered	0	1	0
CSF1R	0.68	6.83E-10	Unclustered	0	1	0
CTBP1	−0.67	5.00E-04	Unclustered	0	1	0
CTF1	0.89	3.34E-05	Unclustered	0	0	0
CTNNB1	1.97	8.41E-04	Unclustered	0.133333	6	0.058742
CX3CR1	1.60	2.19E-07	Unclustered	0.666667	7	3.38E-04
CXCL1	1.14	6.72E-06	Cluster 1	0.6	10	0.015026
CXCL12	4.66	2.38E-04	Cluster 1	0.45	16	0.136083
CXCL6	0.98	3.44E-17	Unclustered	1	3	0
CXCL8	1.39	8.33E-04	Cluster 1	0.538462	13	0.027678
CXCR4	0.52	1.92E-07	Cluster 1	0.5	13	0.097876
CXCR6	0.71	1.97E-05	Unclustered	0.833333	4	1.06E-05
CYBB	−0.72	1.21E-09	Unclustered	0	3	0.026234
DDIT3	−1.00	5.57E-04	Unclustered	0	1	0
DUSP1	0.82	1.18E-04	Cluster 6	1	2	0
DUSP16	−0.71		Unclustered	0	1	0
EDN1	0.70	1.98E-04	Unclustered	0	1	0
EFNA1	−2.54	3.83E-04	Unclustered	0	0	0
EFNA4	−0.44	9.19E-05	Unclustered	0	0	0
EGF	1.08	5.37E-08	Unclustered	0.178571	8	0.044319
ELK1	1.12	3.28E-04	Cluster 6	0.333333	3	0.006117
ENAH	−0.29	3.88E-09	Unclustered	0.666667	3	3.34E-04
FAS	0.44	1.49E-10	Unclustered	0.333333	3	0.022936
FGF4	−1.95	0.001278	Unclustered	0	1	0
FGF7	−0.77	1.32E-04	Unclustered	0.666667	3	8.30E-04
FGF9	0.27	8.68E-12	Unclustered	0	1	0
FLT1	1.80	2.95E-05	Unclustered	0.166667	4	0.003144
FLT3LG	−1.31	4.07E-04	Unclustered	0	2	1
FN1	−1.53	3.89E-12	Cluster 2	0.153846	14	0.169605
GADD45A	−1.56	2.40E-04	Cluster 7	1	2	0
GADD45B	−2.28	9.80E-09	Cluster 7	1	2	0
GADD45G	−0.81	6.35E-05	Cluster 7	1	2	0
GHR	−0.54	5.63E-07	Unclustered	0	0	0
GNA12	−0.95	2.53E-05	Cluster 9	0.333333	4	0.023683
GNA13	0.58	3.03E-07	Cluster 9	0.666667	3	0.00781
HEY1	−0.50	2.10E-04	Unclustered	0	0	0
HGF	0.60	1.66E-05	Unclustered	0	4	0.011035
HSPA1L	0.99	8.11E-05	Unclustered	0	2	0.010692
IFNAR2	0.52	3.25E-04	Unclustered	1	2	0
IFNGR1	−0.39	9.47E-04	Unclustered	0.5	4	0.005006
IFNGR2	2.37	3.78E-08	Unclustered	1	2	0
IKBKG	0.64	4.51E-04	Cluster 4	0.6	5	0.00319
IL10RA	0.80	5.84E-12	Unclustered	1	2	0
IL16	0.38	0.001091	Unclustered	0	0	0
IL18	0.56	8.19E-05	Unclustered	0.166667	4	0.033162
IL21R	1.04	3.15E-04	Unclustered	0.666667	3	0.001452
IL2RB	1.33	6.03E-04	Unclustered	1	2	0
IL32	−0.28	1.87E-05	Unclustered	0	1	0
IL33	0.93	2.77E-09	Unclustered	1	2	0
IL37	2.00	0.001238	Unclustered	0	1	0
IL7	0.55	1.65E-06	Unclustered	0.3	5	0.004205
IL7R	0.56	1.93E-10	Unclustered	0.333333	3	0.01615
INHBA	0.72	4.68E-04	Unclustered	0	1	0
IRAK1	0.64	1.03E-04	Unclustered	0.5	4	0.005121
ITGA2	0.50	1.43E-06	Cluster 2	0.464286	8	0.022655
ITGA4	1.62	2.84E-08	Cluster 2	0.666667	7	0.004541
ITGAL	0.52	1.69E-04	Cluster 2	0.5	8	0.004652
ITGAM	2.33	1.17E-09	Unclustered	0.666667	4	0.003024
ITGAX	0.72	1.61E-04	Unclustered	1	3	0
ITGB2	1.28	4.79E-14	Cluster 2	0.418182	11	0.026134
ITGB7	−1.47	6.25E-05	Cluster 2	0.619048	7	0.05336
JAK1	0.37	4.40E-05	Unclustered	0.181818	12	0.071918
KNG1	0.95	8.84E-04	Unclustered	0	0	0
LAMA2	0.57	3.87E-06	Cluster 8	1	2	0
LAMB1	0.64	3.91E-04	Cluster 8	1	2	0
LAMB3	0.83	1.46E-04	Unclustered	0	1	0
LAMC1	0.91	1.92E-05	Cluster 8	1	2	0
LAMC2	2.19	2.38E-09	Unclustered	0	1	0
LPAR1	−2.54	3.85E-08	Cluster 9	1	2	0
LTB	0.84	1.19E-08	Unclustered	0	0	0
MAGI2	−0.48	1.20E-05	Unclustered	1	2	0
MAP4K1	0.88	2.60E-06	Unclustered	0	1	0
MAPK13	−0.50	3.44E-06	Cluster 6	0.2	5	0.047386
MAPK8	−0.38	7.42E-05	Cluster 6	0.055556	9	0.158478
MAPK8IP2	0.46	0.001162	Unclustered	0	2	0.015873
MET	0.76	1.28E-04	Unclustered	0.111111	10	0.17753
MMP2	1.00	2.10E-04	Unclustered	0	4	0.033256
MSN	1.30	4.10E-08	Unclustered	0.333333	3	0.02271
MYC	0.90	4.42E-05	Unclustered	0.2	5	0.050895
MYD88	−0.41	8.80E-10	Unclustered	0.333333	7	0.041756
MYL10	0.63	4.08E-04	Unclustered	0	1	0
MYL12B	−0.63	6.94E-04	Unclustered	0	1	0
NGF	−1.20	4.10E-07	Unclustered	0	0	0
NLK	0.33	1.06E-04	Unclustered	0	0	0
PAK2	−0.42	4.08E-04	Unclustered	0	2	0.003293
PAK4	0.40	6.55E-04	Unclustered	1	2	0
PAK6	0.64	3.65E-04	Unclustered	1	2	0
PDGFRA	0.69	3.06E-06	Unclustered	0.4	5	0.006608
PF4V1	1.87	2.60E-05	Unclustered	0.8	5	3.60E-05
PFN1	−0.36	2.95E-09	Unclustered	1	2	0
PHLPP1	−0.44	4.67E-05	Unclustered	0	0	0
PIK3CB	−0.57	6.07E-05	Unclustered	0.186813	14	0.109273
PIP5K1C	1.52	3.66E-06	Unclustered	0.2	6	0.050518
PLA2G4A	0.73	2.38E-09	Unclustered	0	1	0
PLCB4	−1.09	3.17E-04	Unclustered	0	2	0.001472
PLCG2	1.51	4.65E-04	Cluster 5	0.222222	9	0.068073
PRKCB	0.60	1.74E-07	Unclustered	0	5	0.062154
PTEN	1.09	4.16E-06	Unclustered	0.190476	7	0.030663
RAC2	0.39	8.47E-08	Cluster 5	0.111111	10	0.082098
RASA2	−0.77	2.32E-06	Unclustered	0	1	0
RDX	0.58	7.86E-04	Unclustered	1	2	0
RPS6	0.89	4.08E-04	Unclustered	0	1	0
RPS6KA1	0.60	1.41E-04	Unclustered	0	1	0
RPS6KA3	−0.61	1.95E-05	Unclustered	0	1	0
RXRA	0.43	9.86E-06	Unclustered	0	0	0
SCIN	0.68	7.48E-04	Unclustered	0	1	0
SNW1	1.48	2.85E-06	Unclustered	0	0	0
SPP1	0.66	9.67E-07	Unclustered	1	2	0
SSH1	0.55	1.46E-04	Unclustered	0	0	0
STAT3	0.77	5.98E-04	Unclustered	0.127273	11	0.167661
SYK	−0.40	1.01E-08	Cluster 5	0.5	5	0.004511
TCL1B	3.42	4.39E-04	Unclustered	0	0	0
THBS2	0.93	2.12E-12	Unclustered	0	0	0
TLR2	0.33	2.49E-09	Unclustered	0.133333	6	0.069196
TMSB4Y	1.68	2.59E-04	Unclustered	0	1	0
TNC	1.11	1.89E-13	Unclustered	0	0	0
TNFRSF11B	1.96	1.54E-04	Unclustered	0	1	0
TNFRSF17	0.65	2.24E-05	Unclustered	0	0	0
TNFRSF1A	0.93	6.06E-05	Cluster 4	0.333333	6	0.01659
TNFRSF1B	1.14	2.62E-07	Unclustered	1	2	0
TNFSF10	0.91	1.21E-06	Unclustered	0.333333	3	0.015873
TNFSF15	0.85	4.47E-08	Unclustered	0	0	0
VAV1	1.37	5.47E-06	Cluster 5	0.533333	6	0.007671
VCAM1	−1.66	6.39E-07	Cluster 2	0.254545	11	0.125962
VWF	1.73	1.37E-08	Unclustered	0	2	0.00994
XCL1	0.42	7.87E-05	Unclustered	0.866667	6	2.90E-04
YWHAH	1.02	3.82E-06	Unclustered	0	1	0

### 58 genes formed a tightly regulated network

STRING integrated in Cytoscape was used to generate a PPI network for 167 DEGs at 0.90 confidence level. 9 highly connected regions, or clusters were found in the network, according to module analysis done with MCODE. We also studied the topological properties of the gene using Network Analyzer plugin of the software. We checked for their betweenness centrality, degree, and clustering co-efficient and a tightly regulated network with 58 genes (Table 3). CXCR4, CXCL8, and CCR5 had highest number of connections.

**Table 3:** 58 genes from the condensed network.

ACKR4	CCR5	CXCR4	IFNGR1	ITGAM	PF4V1
ACTN1	CCR6	CXCR6	IFNGR2	ITGAX	PFN1
ACTR3	COL1A2	DUSP1	IKBKG	ITGB7	RDX
ARPC1B	COL3A1	ENAH	IL10RA	LAMA2	SPP1
ARPC3	COL6A2	FGF7	IL21R	LAMB1	SYK
CCL19	COL6A3	GADD45A	IL2RB	LAMC1	TNFRSF1B
CCL2	CX3CR1	GADD45B	IL33	LPAR1	VAV1
CCL21	CXCL1	GADD45G	IRAK1	MAGI2	XCL1
CCNE1	CXCL6	GNA13	ITGA4	PAK4	
CCR2	CXCL8	IFNAR2	ITGAL	PAK6	

### Chemokine markers, ECM/ fibrotic markers and stress response markers were the central players

**Fig 4:**
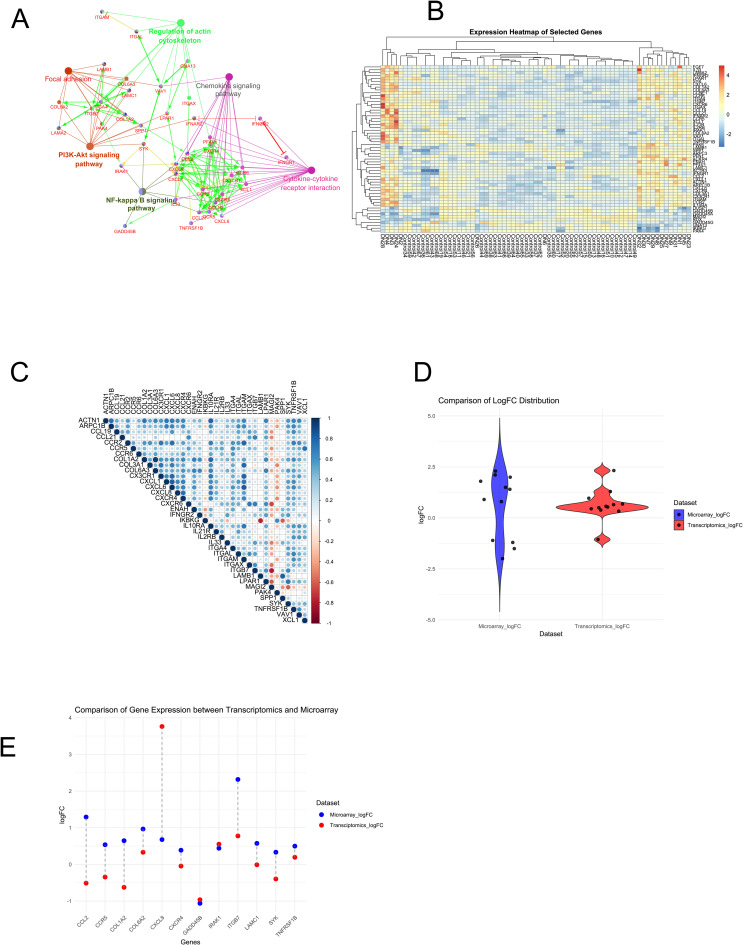
Chemokine markers, ECM/ fibrotic markers and stress response markers were the central players. A. Network shows the interaction between the 58 core genes and the pathways. Edges in green, yellow and red depict activation, expression and inhibition respectively. The network depicts 3 major clusters- inflammatory cluster (CXCR4, CCR5, CXCL6, CCL19, TNFRSF1B, IKBKG, IRAK1), signaling cluster (PI3K-AKT signaling), and fibrosis cluster (COL1A2, COL6A3, LAMB1, ITGB7). B. Heatmap shows differential expression of the 58 genes in the condensed network based on their expression. Most of the genes were positively correlated except for IKBKG, LAMB1, MAGI2, and PAK4 suggesting that inflammation and ECM genes are tightly regulated together. C. Correlation plot highlight 27 genes whose p values were ≤ 1e-14. The upregulated genes (depicted in red) point at their potential involvement in the disease progression and may serve as biomarkers or therapeutic targets for AGE induced DN, while downregulated genes (in blue) indicate alteration and suppression in the signaling of the regulatory mechanism. D. The violin plot shows the distribution of expressional fold changes of the 12 driver genes across the microarray dataset (existing dataset) (blue violin) and regional transcriptomic dataset (independent dataset) (red violin) from KPMP public database. E. The scatter plot with connected lines shows difference in expression in the two datasets. The blue dots represent expression values of existing dataset and the red dot depicts expression values of independent dataset. The graph shows consistent pattern of expression in COL6A2, ITGB7, IRAK1, TNFRSF1B, GADD45B. CXCL8 is shows upregulation in both sets but the long connecting line indicates difference in the expression level.

To get more comprehensive idea about how these 58 genes interact with each other, we used Cluepedia app from Cytoscape. We realized these genes were majorly involved with Cytokine-cytokine receptor interaction, Regulation of actin cytoskeleton, PI3K-Akt signaling pathway, Chemokine signaling pathway, Focal adhesion, and NF-kB signaling pathway. Cluepedia showed CXCL8, CCL2, CCR5, CXCR4, COL1A2, COL6A2, LAMC1, ITGB7, SYK, IRAK1, TNFRSF1B, GADD45B were the key players in the network (Fig 4A). The expression pattern of the 58 genes across samples has been depicted as a heatmap in Fig 4B. Among several cytokines, CCL2 is a significant driver based on its logFC and centrality measures. To get better insights into the interaction of the pathways and the genes involved we constructed a correlation plot. The plot was constructed to highlight genes whose p values were ≤ 1e-14 and identified 27 genes, which was plotted as a matrix (Fig 4C). The plot elucidated that except for IKBKG, LAMB1, MAGI2, and PAK4 all other genes were positively correlated. These 4 genes are also downregulated in DN condition.

### 15 genes were found to be common upon cross-platform validation with RNA-Seq data

**Fig 5:**
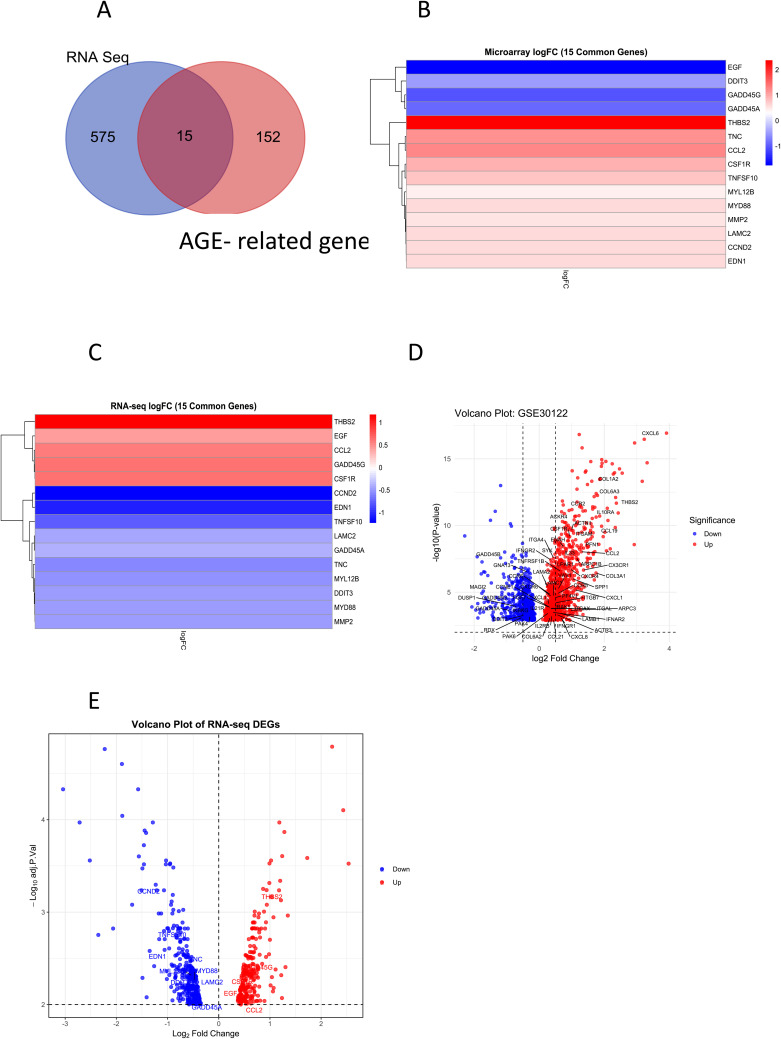
15 genes were found to be common upon cross-platform validation with RNA-Seq data. A. Venn diagram representing 15 genes are common between signifiant RNA seq dataset and the 167 genes related to AGEs obtained from microarray dataset. B: Heatmap representing the logFC of expression values 15 genes from microarray dataset. C. Heatmap representing the logFC of the quantified transcript expression values 15 genes from RNA Seq dataset. DDIT3, GADD45A, THBS2, CCL2, and CSF1R showed consistent expression trends across platforms. D. Volcano plot showing the differentially expressed genes from microarray dataset GSE30122. 58 core genes from the condensed gene network along with the 5 genes that were consistently altered across microarray and RNA-seq dataset are highlighted. Genes significantly upregulated are shown in red, while significantly downregulated genes are shown in blue. E. Volcano plot showing the differentially expressed genes from RNA-seq dataset GSE299230. 15 genes from the condensed gene network from microarray dataset GSE30122 and RNA-seq dataset GSE299230 including the 5 genes that were consistently altered across microarray and RNA-seq dataset are highlighted. Genes significantly upregulated are shown in red, while significantly downregulated genes are shown in blue.

To independently test whether the presumed core genes were differentially expressed in a DKD-relevant system, we analyzed RNA-seq dataset GSE299230. From GSE299230, a total of 12665 DEGs were identified, of which 246 were upregulated and 344 were downregulated significantly. Comparison with the 167 AGE-related genes from GSE30122 revealed 15 common genes: CCND2, THBS2, TNFSF10, EDN1, TNC, GADD45G, CSF1R, MYL12B, MYD88, LAMC2, DDIT3, MMP2, EGF, GADD45A, and CCL2 (Fig 5A, Table 4). DDIT3, GADD45A, THBS2, CCL2, and CSF1R showed consistent expression trends across platforms (Fig 5B and 5C), and are known mediators of inflammation, extracellular matrix remodeling, and stress responses in diabetic kidney disease. DDIT3 and GADD45A were consistently downregulated, while THBS2, CCL2, and CSF1R were consistently upregulated (Fig 5D and 5E). However, DDIT3, THBS2, and CSF1R were not part of the sub-network of 58 genes on which we are focussing in this work. This partial convergence highlights both the strengths and the contextual limits of our findings. This computational replication, nevertheless, strengthens the credibility of the proposed network.

**Table 4:** List of the common genes between microarray dataset and RNA Seq dataset.

Common genes
MMP2
EDN1
TNC
MYD88
GADD45G
MYL12B
GADD45A
DDIT3
TNFSF10
THBS2
EGF
LAMC2
CCND2
CSF1R
CCL2

### Five genes showed consistent trends in the independent dataset validation

The existing microarray dataset was also cross-validated with independent regional transcriptomic datasets available in KPMP database (https://atlas.kpmp.org/). The violin plot (Fig 4D) showed the distribution of expressional fold changes of the 12 driver genes across the microarray dataset (existing dataset) (blue violin) and regional transcriptomic dataset (independent dataset) (red violin) from KPMP public database. Greater variability can be seen in the transcriptomic dataset than the microarray. We observed of the 12 key driver genes (CXCL8, CCL2, CCR5, CXCR4, COL1A2, COL6A2, LAMC1, ITGB7, SYK, IRAK1, TNFRSF1B, GADD45B) 5 genes viz. COL6A2, ITGB7, IRAK1, TNFRSF1B, GADD45B showed similar expression trends strengthening the relevance of these genes in the context of AGE-induced DN (Fig 4E, Supplementary table 4 in S1 File). CXCL8 is upregulated in both sets but shows stronger expression in transcriptomic dataset. Differences in the distribution projected by violin plot may be due to platform-specific biases.

## Discussion

Persistent hyperglycemia results in the accumulation of AGEs in both tissues and blood. Kidneys play major role in clearing out the AGEs, hence their accumulation has been linked to the development of diabetic nephropathy, which is a major complication of diabetes and increases risk for end-stage renal disease and mortality [32,53]. Recent study from our laboratory suggests that elevated levels of AGEs are implicated in the reactivation of Notch signaling in podocytes. Notch signaling is crucial for fate determination of podocytes during embryological development, however, reactivation of Notch in adult podocytes induces loss of epithelial features and compromises its permselective potential [11]. However, the mechanistic understanding of how AGEs induce EMT is still lacking. Uncovering the signaling events that integrate and activate Notch signaling during exposure to AGEs could potentially improve our understanding about the pathophysiology of diabetic kidney disease and also guide us to develop novel intervention strategies. Network biology aids in understanding large-scale characteristics of various biological processes and in identifying specific biological attributes [54]. Therefore, we utilized network biology approach to identify genes and pathways that might play crucial role in reactivating Notch signaling.

We performed an array of bioinformatic analyses on GSE30122 to draw a gene interaction network that can highlight the molecular pathways that implicate the reactivation of Notch signaling in AGEs induced DN. Functional enrichment analysis on the dataset revealed that PI3K-AKT pathway is highly relevant in the pathogenesis of DN. The analysis also highlighted that inflammatory signaling pathways, and ECM related pathways were enriched in DN condition, with 156 unique genes contributing to these pathways. Additionally, 21 genes involved in AGE-RAGE signaling and 3 genes involved with Notch signaling were also identified. AGE-RAGE interaction is known to trigger oxidative stress in renal tissues by increasing ROS levels, which sets the wheel in motion for PI3K-AKT signaling [33]. Together, we proceeded to look into these 167 genes to understand the reactivation mechanism.

Prioritizing the 167 genes we created a protein interaction network via STRING app of Cytoscape. Among them 58 showed high connectivity based on their topological properties such as degree, betweenness centrality, and clustering coefficient (Table 2). Hence, we created a sub-network with this condensed interaction. This core was reanalyzed for highlighting the core pathways. Apart from the previous mentioned pathways the core was associated with Chemokine signaling pathway, and NF-kB signaling pathway. PI3K-AKT signaling [33] induced by AGE-RAGE interaction can activate NF-kB [55].

In our dataset CCNE1, COL1A2, COL6A2, COL6A3, FGF7, IFNAR2, IKBKG, IL2RB, ITGA4, ITGB7, LAMA2, LAMB1, LAMC1, LPAR1, MAGI2, SPP1, and SYK were associated with PI3K-AKT signaling. COL6A2 and ITGB7 showed similar expression pattern in both independent and existing dataset. Most of these differentially expressed genes were responsible for focal adhesion and regulation of actin cytoskeleton, thereby justifying enrichment of the EMT hallmark from GSEA, except for IFNAR2, IKBKG, and IL2RB, which were associated with inflammation.

Down regulation of laminins (LAMA2, LAMB1, and LAMC1) and integrins (ITGA4, ITGAL, ITGAX, and ITGB7) were observed in our dataset. LAMB1 (log2FC = .95, p = 0.00039) was negatively correlated to IKBKG (β = −0.8) and, IL21R (β = −0.05), ITGAX (β = .-0.06), MAGI2 (β = −0.33) and PAK4 (β = −0.60). LAMA2 (log2FC = .37, p = 3.87E-06), a key molecule in the PI3K-AKT pathway, is known to activate cell migration [56]. Down regulation of laminins and integrins are associated with modulation of focal adhesion, which is also corroborated from KEGG analysis [57]. CCNE1 and MAGI2 were unique to PI3K-AKT signaling. All these genes, except COL1A2, COL6A3, and SPP1 were downregulated. Interaction of RAGE with AGEs is linked with PI3K-AKT signaling. Reports suggest RAGE is a multiligand receptor that activates PI3K-AKT signaling but upon interaction with AGEs leads to inhibition of the signaling, which induces autophagy. Administration of RAGE neutralizing antibody, PI3K/AKT signaling agonist and/ or AKT inhibitors can revert the inhibition [58–60]. Down regulation of CCNE1 (log2FC = 0.48, p = 0.000140249) indicated dysregulation in podocyte phenotype [61]. MAGI2 (log2FC = −2.54, p = 1.20E-05) has been reported to have a protective role in the podocytes and its down regulation indicates a decrease in anti-apoptotic proteins and is correlated to podocyte loss [62]. Just like Notch, COL1A2 (log2FC = 3.34, p = 1.12E-14) is active during developmental stages but gets attenuated in adults [63]. COL6A3 (log2FC = 3.38, p =  7.94E-13) and SPP1(log2FC = 1.48, p = 9.67E-07) have been proposed to be profibrotic genes that participate in immunologic modulation. The upregulation of these genes indicates kidney injury [64,65]. Down regulation of the rest of the genes due to low PI3K-AKT signaling led to dysregulation of focal adhesion and regulation of cytoskeleton. Chronic hypergylcemia result in down regulation of PI3K-AKT signaling which is evident from the dysregulation in the expression profiles of the genes involved with the signaling. Since PI3K-AKT signaling is a survival pathway, its down regulation in DN condition manifests as podocytopathy. Findings from biopsies of FSGS patients have also shown decreased expression of pSer473-Akt, showing progression to end stage kidney disease [66].

The network evidently showed inflammatory signals were the major drivers in DN pathogenesis. The inflammatory markers, some of which are also involved with NF-kB signaling, are positively correlated amongst themselves as well as with certain ECM and cytoskeletal markers (Fig 4C) indicating their tight regulation. A previous report from our lab also showed exposure of renal cells with N-carboxymethyl-lysine (CML), a predominant type of AGEs, induced Zeb2, a transcription factor of EMT via NF-kB signaling, thereby affecting podocyte integrity [67]. Genes viz IKBKG (log2FC = −0.38, p = .00045) and SYK (log2FC=0.77, p = 1.01E-08) both of which participated in both NF-kB signaling and PI3K-AKT signaling were found to downregulated. IKBKG was negatively correlated with most of the other genes (β_COL1A2_ = −0.27, β_COL6A3_ = −0.33, β_CX3CR1_ = −0.21). Both these genes are crucial for mediating NF-kB signaling, which is a key cell survival pathway and their down regulation leads to fibrosis. In steatohepatitis knockdown of IKBKG resulted in fibrosis, while inhibition of SYK decreased fibrosis in kidney [68,69]. Upregulation was observed in chemokine and cytokine regulating genes (ACKR4 (log2FC = 1.18, p = 6.12E-11), CCL19 (log2FC 2.84, p = 6.86E-10), CCL2 (log2FC = 1.86. p = 9.33E-09), CCR2 (log2FC = 1.45, p = 6.43E-12), CX3CR1 (log2FC = 1.97, p = 2.19E-07), CXCL1 (log2FC = 1.60, p = 6.72E-06), CXCL6 (log2FC = 4.66, p = 3.44E-17), CXCR4 (log2FC = 1.39, p = 1.92E-07), IL10RA (log2FC = 2.37, p = 5.84E-12), and IL33 (log2FC = 1.33, p = 2.77E-09)) indicating an increase in recruitment and activation of inflammatory pathways [70]. The differential levels of cytokines and chemokines boost inflammation which affects ECM remodeling as evident from the expression profiles of the gene participating in focal adhesion and regulation of actin cytoskeleton. These levels of cytokines and chemokines lead to constitutive activation of NF-kB signaling which can activate Notch signaling [71]. This is known to exacerbate podocyte injury, a classic hallmark of DN insult [72]. Damaged kidney cells recruit macrophages that are polarized into the proinflammatory M1 phenotype and participate in the death of intrinsic kidney cells. The crosstalk between Notch and key NF-kB molecules favors this polarization and aggravates the tissue damage, providing a new direction of therapeutic target for diabetic nephropathy in the future [73].

Our cross-platform results identified a condensed AGE-associated network with several inflammation and ECM-related mediators. Importantly, DDIT3, GADD45A, THBS2, CCL2 and CSF1R were consistently altered across microarray and RNA-seq datasets, implicating stress (DDIT3), DNA damage/repair stress pathways (GADD45A), matricellular remodeling (THBS2), monocyte recruitment (CCL2) and macrophage signaling (CSF1R) in AGE-induced perturbations of renal cells. Although DDIT3, THBS2, and CSF1R were not part of the sub-network of 58 genes and not discussed in detail above. Recent studies corroborate roles for DDIT3 in renal fibrosis and ER stress-driven injury [74]. Interestingly, in both our datasets DDIT3 is downregulated. Reports by Kong et al. suggest upregulation of DDIT3 promotes fibrosis in diabetic kidney whereas report by Li-Li Ma et al. suggest DDIT3 is downregulated in DN condition [75]. This may be an adaptive response to attenuate persistent ER stress rather. GADD45A/B, THBS2 are identified as biomarkers and functional modulators in acute renal injury and DKD [76–78]. The centrality of CCL2 has been discussed above. These reports place our findings within the contemporary literature and highlight the translational potential of targeting inflammation/macrophage and matricellular remodeling pathways in AGE-driven DN.

**Fig 6:**
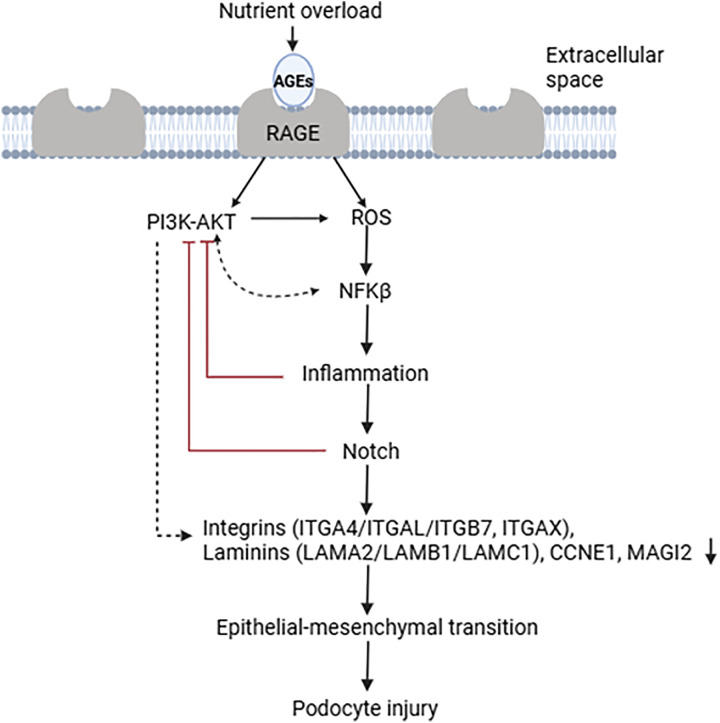
Interaction between AGE-RAGE induces ROS production and typically sets on PI3K-AKT pathway. The simultaneous influence of both activates the NFKB signaling which elicits inflammatory responses. The induction of the NFKB signaling also triggers NICD1, thus leading to non-cannonical activation of the Notch signaling pathway. This reactivation suppresses PI3K-AKT signaling and disregulates the expression of integrins (ITGA4, ITGAL, ITGB7, ITGAX) and laminins (LAMA2, LAMB1, LAMC1), along with structural proteins like CCNE1 and MAGI2, leading to ECM remodeling and changes in cell adhesion and migration. These molecular changes contribute to EMT, where podocytes lose their epithelial characteristics and acquire mesenchymal-like properties, ultimately leading to podocyte injury.

Collectively our study showed that AGEs fostered by chronic hyperglycemia suppress PI3K-AKT pathway, activate NF-kB signaling and elicit inflammatory responses. Both these responses have been shown to reactivate Notch signaling [79,80]. On the other hand, reactivation of Notch also suppresses PI3K-AKT signaling. This induces apoptosis in the terminally differentiated podocytes [81,82] (Fig 6).

## Conclusion

The study provides novel information about AGEs induced podocyte injury. AGEs induced selective network of 58 genes in podocytes and are associated with non-canonical reactivation of Notch signaling, probably via activating NF-kB signaling and supressing PI3K-AKT signaling. These signaling cascades that are selectively induced in podocytes under the stimulus of AGEs could be explored as therapeutic targets to prevent the progression of diabetic kidney disease. Our earlier experimental studies also suggest reactivation of Notch signaling is implicated in the podocytes [11].

## Limitations

The public RNA-seq dataset used for validation (GSE299230) is an in vitro HK-2 cell model (hnRNPF overexpression; n = 3 per group) rather than human patient tissue; the small sample size and cellular model limit direct generalizability to complex human kidney disease. Second, for comparisons across platforms (microarray GSE30122 vs RNA-seq GSE299230), platform-specific biases (probe design, dynamic range, normalization) can influence observed concordance; we therefore used conservative thresholds and variance-shrinkage methods and present GSE299230 results as supportive, not definitive, evidence. Finally, our study is computational and correlative; experimental validation (knockdown/overexpression, protein-level assays, in vivo models) is necessary to confirm the mechanistic roles of condensed gene network in AGE-driven podocyte/renal injury.

## Supporting information

S1 File **S1 Table.** List of up and down regulated genes from GSE 30122. **S2 Table.** KEGG Pathways associated with DEGs. **S3 Table.** Hallmarks in the dataset obtained from GSE analysis. **S4 Table.** Trend for logFC within the existing data and independent data from KPMP Database.
(ZIP)

## References

[1] Moucheraud C, Lenz C, Latkovic M, Wirtz VJ. The costs of diabetes treatment in low- and middle-income countries: a systematic review. BMJ Glob Health. 2019;4(1):e001258. doi: 10.1136/bmjgh-2018-001258 30899566 PMC6407562

[2] Mekala KC, Bertoni AG. Epidemiology of diabetes mellitus. Transplantation, bioengineering, and regeneration of the endocrine pancreas. Elsevier. 2020. p. 49–58.

[3] Gheith O, Farouk N, Nampoory N, Halim MA, Al-Otaibi T. Diabetic kidney disease: world wide difference of prevalence and risk factors. J Nephropharmacol. 2015;5(1):49–56. 28197499 PMC5297507

[4] Gupta S, Dominguez M, Golestaneh L. Diabetic kidney disease: an update. Med Clin North Am. 2023;107(4):689–705. 37258007 10.1016/j.mcna.2023.03.004

[5] Samsu N. Diabetic nephropathy: challenges in pathogenesis, diagnosis, and treatment. Biomed Res Int. 2021;2021:1497449. 34307650 10.1155/2021/1497449 PMC8285185

[6] Anil Kumar P, Welsh GI, Saleem MA, Menon RK. Molecular and cellular events mediating glomerular podocyte dysfunction and depletion in diabetes mellitus. Front Endocrinol (Lausanne). 2014;5:151. doi: 10.3389/fendo.2014.00151 25309512 PMC4174857

[7] Efiong EE, Maedler K, Effa E, Osuagwu UL, Peters E, Ikebiuro JO, et al. Decoding diabetic kidney disease: a comprehensive review of interconnected pathways, molecular mediators, and therapeutic insights. Diabetol Metab Syndr. 2025;17(1):192. doi: 10.1186/s13098-025-01726-4 40468401 PMC12139089

[8] Khoury CC, Chen S, Ziyadeh FN. Pathophysiology of diabetic nephropathy. Chronic renal disease. Elsevier. 2020. p. 279–96.

[9] Zeng L, Szeto C-C. Urinary podocyte markers in kidney diseases. Clin Chim Acta. 2021;523:315–24. doi: 10.1016/j.cca.2021.10.017 34666027

[10] Ricciardi CA, Gnudi L. Kidney disease in diabetes: From mechanisms to clinical presentation and treatment strategies. Metabolism. 2021;124:154890. doi: 10.1016/j.metabol.2021.154890 34560098

[11] Nishad R, Meshram P, Singh AK, Reddy GB, Pasupulati AK. Activation of Notch1 signaling in podocytes by glucose-derived AGEs contributes to proteinuria. BMJ Open Diabetes Res Care. 2020;8(1):e001203. doi: 10.1136/bmjdrc-2020-001203 32601154 PMC7326296

[12] Pasupulati AK. Podocyte developmental pathways in diabetic nephropathy: A spotlight on Notch signaling. Diabetic Nephropathy. 2022;2(1):1–6. doi: 10.2478/dine-2022-0015

[13] Sirin Y, Susztak K. Notch in the kidney: development and disease. J Pathol. 2012;226(2):394–403. doi: 10.1002/path.2967 21952830 PMC3677191

[14] Mukherjee M, Fogarty E, Janga M, Surendran K. Notch Signaling in Kidney Development, Maintenance, and Disease. Biomolecules. 2019;9(11). 10.3390/biom9110692 PMC6920979 31690016

[15] Barutta F, Bellini S, Gruden G. Mechanisms of podocyte injury and implications for diabetic nephropathy. Clin Sci (Lond). 2022;136(7):493–520. doi: 10.1042/CS20210625 35415751 PMC9008595

[16] Liu WJ, Reiser J, Park TS, Liu Z, Ishibe S. New Insights into Diabetic Kidney Disease: The Potential Pathogenesis and Therapeutic Targets. J Diabetes Res. 2017;2017:3945469. doi: 10.1155/2017/3945469 29250556 PMC5698813

[17] Wyss J-C, Kumar R, Mikulic J, Schneider M, Aebi JD, Juillerat-Jeanneret L, et al. Targeted γ-secretase inhibition of Notch signaling activation in acute renal injury. Am J Physiol Renal Physiol. 2018;314(5):F736–46. doi: 10.1152/ajprenal.00414.2016 28971991

[18] Marquez-Exposito L, Cantero-Navarro E, Lavoz C, Fierro-Fernández M, Poveda J, Rayego-Mateos S, et al. Could Notch signaling pathway be a potential therapeutic option in renal diseases?. Nefrologia (Engl Ed). 2018;38(5):466–75. doi: 10.1016/j.nefro.2017.11.027 29439807

[19] Kato S, Matsumura T, Sugawa H, Nagai R. Correlation between serum advanced glycation end-products and vascular complications in patient with type 2 diabetes. Sci Rep. 2024;14(1):18722. doi: 10.1038/s41598-024-69822-5 39134632 PMC11319737

[20] Schalkwijk CG, Micali LR, Wouters K. Advanced glycation endproducts in diabetes-related macrovascular complications: focus on methylglyoxal. Trends Endocrinol Metab. 2023;34(1):49–60. doi: 10.1016/j.tem.2022.11.004 36446668

[21] Cooksley G, Nam M-H, Nahomi RB, Rankenberg J, Smith AJO, Wormstone YM, et al. Lens capsule advanced glycation end products induce senescence in epithelial cells: Implications for secondary cataracts. Aging Cell. 2024;23(10):e14249. doi: 10.1111/acel.14249 39384405 PMC11464126

[22] Saxena S, Mishra N, Cheung G, Sadda SR. Unravelling advanced glycation end products as emerging therapeutic targets in diabetic retinopathy management. Eur J Ophthalmol. 2025;35(5):1527–30. doi: 10.1177/11206721251328562 40123240

[23] Zgutka K, Tkacz M, Tomasiak P, Tarnowski M. A role for advanced glycation end products in molecular ageing. Int J Mol Sci. 2023;24(12). 10.3390/ijms24129881 PMC10298716 37373042

[24] Niranjan T, Bielesz B, Gruenwald A, Ponda MP, Kopp JB, Thomas DB, et al. The Notch pathway in podocytes plays a role in the development of glomerular disease. Nat Med. 2008;14(3):290–8. doi: 10.1038/nm1731 18311147

[25] Nishad R, Mukhi D, Tahaseen SV, Mungamuri SK, Pasupulati AK. Growth hormone induces Notch1 signaling in podocytes and contributes to proteinuria in diabetic nephropathy. J Biol Chem. 2019;294(44):16109–22. doi: 10.1074/jbc.RA119.008966 31511328 PMC6827306

[26] Tan H, Xu W, Ding X, Ye H, Hu Y, He X, et al. Notch/NICD/RBP-J signaling axis regulates M1 polarization of macrophages mediated by advanced glycation end products. Glycoconj J. 2022;39(4):487–97. doi: 10.1007/s10719-022-10062-y 35666407

[27] Kumar Pasupulati A, Chitra PS, Reddy GB. Advanced glycation end products mediated cellular and molecular events in the pathology of diabetic nephropathy. Biomol Concepts. 2016;7(5–6):293–309. doi: 10.1515/bmc-2016-0021 27816946

[28] Xue L, Zhang Y, Zhang Q. The relationship between advanced glycation end products, metabolic metrics, HbA1c, and diabetic nephropathy. Front Endocrinol (Lausanne). 2025;16:1468737. doi: 10.3389/fendo.2025.1468737 40123890 PMC11925793

[29] Diallo AM, Jaisson S, Barriquand R, Lukas C, Barraud S, Decoudier B, et al. Association Between the Tissue and Circulating Advanced Glycation End-Products and the Micro- and Macrovascular Complications in Type 1 Diabetes: The DIABAGE Study. Diabetes Ther. 2022;13(8):1531–46. doi: 10.1007/s13300-022-01285-1 35779209 PMC9309113

[30] Lee J, Yun J-S, Ko S-H. Advanced Glycation End Products and Their Effect on Vascular Complications in Type 2 Diabetes Mellitus. Nutrients. 2022;14(15):3086. doi: 10.3390/nu14153086 35956261 PMC9370094

[31] Zhang Y, Wang Y, Kang Q, Chen Y, Ai L, Hu K, et al. The role of advanced glycation end products between thyroid function and diabetic nephropathy and metabolic disorders. Sci Rep. 2025;15(1):7202. doi: 10.1038/s41598-025-88806-7 40021692 PMC11871035

[32] Koska J, Gerstein HC, Beisswenger PJ, Reaven PD. Advanced Glycation End Products Predict Loss of Renal Function and High-Risk Chronic Kidney Disease in Type 2 Diabetes. Diabetes Care. 2022;45(3):684–91. doi: 10.2337/dc21-2196 35051276 PMC8918197

[33] Wu X-Q, Zhang D-D, Wang Y-N, Tan Y-Q, Yu X-Y, Zhao Y-Y. AGE/RAGE in diabetic kidney disease and ageing kidney. Free Radic Biol Med. 2021;171:260–71. doi: 10.1016/j.freeradbiomed.2021.05.025 34019934

[34] Pasupulati AK, Nagati V, Paturi AS, Reddy GB. Non-enzymatic glycation and diabetic kidney disease. 2024. 10.1016/bs.vh.2024.01.002 38997166

[35] Thomas MC, Forbes JM, Cooper ME. Advanced glycation end products and diabetic nephropathy. Am J Ther. 2005;12(6):562–72. doi: 10.1097/01.mjt.0000178769.52610.69 16280650

[36] Bohlender JM, Franke S, Stein G, Wolf G. Advanced glycation end products and the kidney. Am J Physiol Renal Physiol. 2005;289(4):F645-59. doi: 10.1152/ajprenal.00398.2004 16159899

[37] Nishad R, Tahaseen V, Kavvuri R, Motrapu M, Singh AK, Peddi K, et al. Advanced-Glycation End-Products Induce Podocyte Injury and Contribute to Proteinuria. Front Med (Lausanne). 2021;8:685447. doi: 10.3389/fmed.2021.685447 34277660 PMC8280521

[38] Kim Y. Blood and Tissue Advanced Glycation End Products as Determinants of Cardiometabolic Disorders Focusing on Human Studies. Nutrients. 2023;15(8):2002. doi: 10.3390/nu15082002 37111220 PMC10144557

[39] Matsui T, Sotokawauchi A, Nishino Y, Koga Y, Yamagishi S-I. Empagliflozin ameliorates renal and metabolic derangements in obese type 2 diabetic mice by blocking advanced glycation end product-receptor axis. Mol Med. 2025;31(1):88. doi: 10.1186/s10020-025-01138-0 40050708 PMC11887197

[40] Erbe R, Gore J, Gemmill K, Gaykalova DA, Fertig EJ. The use of machine learning to discover regulatory networks controlling biological systems. Mol Cell. 2022;82(2):260–73. doi: 10.1016/j.molcel.2021.12.011 35016036 PMC8905511

[41] Somvanshi PR, Tomar M, Kareenhalli V. Computational Analysis of Insulin-Glucagon Signalling Network: Implications of Bistability to Metabolic Homeostasis and Disease states. Sci Rep. 2019;9(1):15298. doi: 10.1038/s41598-019-50889-4 31653897 PMC6814820

[42] Davis S, Meltzer PS. GEOquery: a bridge between the Gene Expression Omnibus (GEO) and BioConductor. Bioinformatics. 2007;23(14):1846–7. doi: 10.1093/bioinformatics/btm254 17496320

[43] Gautier L, Cope L, Bolstad BM, Irizarry RA. affy--analysis of Affymetrix GeneChip data at the probe level. Bioinformatics. 2004;20(3):307–15. doi: 10.1093/bioinformatics/btg405 14960456

[44] Leek JT, Johnson WE, Parker HS, Jaffe AE, Storey JD. The sva package for removing batch effects and other unwanted variation in high-throughput experiments. Bioinformatics. 2012;28(6):882–3. doi: 10.1093/bioinformatics/bts034 22257669 PMC3307112

[45] Ritchie ME, Phipson B, Wu D, Hu Y, Law CW, Shi W, et al. limma powers differential expression analyses for RNA-sequencing and microarray studies. Nucleic Acids Res. 2015;43(7):e47. doi: 10.1093/nar/gkv007 25605792 PMC4402510

[46] Yu G, Wang L-G, Han Y, He Q-Y. clusterProfiler: an R package for comparing biological themes among gene clusters. OMICS. 2012;16(5):284–7. doi: 10.1089/omi.2011.0118 22455463 PMC3339379

[47] Bindea G, Galon J, Mlecnik B. CluePedia Cytoscape plugin: pathway insights using integrated experimental and in silico data. Bioinformatics. 2013;29(5):661–3. doi: 10.1093/bioinformatics/btt019 23325622 PMC3582273

[48] Otasek D, Morris JH, Bouças J, Pico AR, Demchak B. Cytoscape Automation: empowering workflow-based network analysis. Genome Biol. 2019;20(1):185. doi: 10.1186/s13059-019-1758-4 31477170 PMC6717989

[49] Szklarczyk D, Kirsch R, Koutrouli M, Nastou K, Mehryary F, Hachilif R, et al. The STRING database in 2023: protein-protein association networks and functional enrichment analyses for any sequenced genome of interest. Nucleic Acids Res. 2023;51(D1):D638–46. doi: 10.1093/nar/gkac1000 36370105 PMC9825434

[50] Bader GD, Hogue CWV. An automated method for finding molecular complexes in large protein interaction networks. BMC Bioinformatics. 2003;4:2. doi: 10.1186/1471-2105-4-2 12525261 PMC149346

[51] Shannon P, Markiel A, Ozier O, Baliga NS, Wang JT, Ramage D, et al. Cytoscape: a software environment for integrated models of biomolecular interaction networks. Genome Res. 2003;13(11):2498–504. doi: 10.1101/gr.1239303 14597658 PMC403769

[52] Nithya C, Kiran M, Nagarajaram HA. Comparative analysis of pure hubs and pure bottlenecks in human protein-protein interaction networks. bioRxiv. 2021. doi: 2021.04.06.438602

[53] D’Agati V, Yan SF, Ramasamy R, Schmidt AM. RAGE, glomerulosclerosis and proteinuria: roles in podocytes and endothelial cells. Trends Endocrinol Metab. 2010;21(1):50–6. doi: 10.1016/j.tem.2009.07.003 19783154

[54] Barabási A-L, Oltvai ZN. Network biology: understanding the cell’s functional organization. Nat Rev Genet. 2004;5(2):101–13. doi: 10.1038/nrg1272 14735121

[55] Liu R, Chen Y, Liu G, Li C, Song Y, Cao Z, et al. PI3K/AKT pathway as a key link modulates the multidrug resistance of cancers. Cell Death Dis. 2020;11(9):797. doi: 10.1038/s41419-020-02998-6 32973135 PMC7515865

[56] Liang J, Li H, Han J, Jiang J, Wang J, Li Y, et al. Mex3a interacts with LAMA2 to promote lung adenocarcinoma metastasis via PI3K/AKT pathway. Cell Death Dis. 2020;11(8):614. doi: 10.1038/s41419-020-02858-3 32792503 PMC7427100

[57] Frommherz LH, Sayar SB, Wang Y, Trefzer LK, He Y, Leppert J, et al. Integrin α3 negative podocytes: A gene expression study. Matrix Biol Plus. 2022;16:100119. doi: 10.1016/j.mbplus.2022.100119 36060790 PMC9429797

[58] Ramasubbu K, Devi Rajeswari V. Impairment of insulin signaling pathway PI3K/Akt/mTOR and insulin resistance induced AGEs on diabetes mellitus and neurodegenerative diseases: a perspective review. Mol Cell Biochem. 2023;478(6):1307–24. doi: 10.1007/s11010-022-04587-x 36308670

[59] Li G, Xu J, Li Z. Receptor for advanced glycation end products inhibits proliferation in osteoblast through suppression of Wnt, PI3K and ERK signaling. Biochem Biophys Res Commun. 2012;423(4):684–9. doi: 10.1016/j.bbrc.2012.06.015 22699121

[60] Wang Z, Bao Z, Ding Y, Xu S, Du R, Yan J, et al. Nε-carboxymethyl-lysine-induced PI3K/Akt signaling inhibition promotes foam cell apoptosis and atherosclerosis progression. Biomed Pharmacother. 2019;115:108880. doi: 10.1016/j.biopha.2019.108880 31035012

[61] Barisoni L, Mokrzycki M, Sablay L, Nagata M, Yamase H, Mundel P. Podocyte cell cycle regulation and proliferation in collapsing glomerulopathies. Kidney Int. 2000;58(1):137–43. doi: 10.1046/j.1523-1755.2000.00149.x 10886558

[62] Wang T, Li C, Wang X, Liu F. MAGI2 ameliorates podocyte apoptosis of diabetic kidney disease through communication with TGF-β-Smad3/nephrin pathway. FASEB J. 2023;37(12):e23305. doi: 10.1096/fj.202301058R 37950637

[63] Fragiadaki M, Witherden AS, Kaneko T, Sonnylal S, Pusey CD, Bou-Gharios G, et al. Interstitial fibrosis is associated with increased COL1A2 transcription in AA-injured renal tubular epithelial cells in vivo. Matrix Biol. 2011;30(7–8):396–403. doi: 10.1016/j.matbio.2011.07.004 21864682

[64] Xu D, Jiang C, Xiao Y, Ding H. Identification and validation of disulfidptosis-related gene signatures and their subtype in diabetic nephropathy. Front Genet. 2023;14:1287613. doi: 10.3389/fgene.2023.1287613 38028597 PMC10658004

[65] Ye Q, Xu G, Xue C, Pang S, Xie B, Huang G, et al. Urinary SPP1 has potential as a non-invasive diagnostic marker for focal segmental glomerulosclerosis. FEBS Open Bio. 2023;13(11):2061–80. doi: 10.1002/2211-5463.13704 37696527 PMC10626280

[66] Chhaing R, Ma Q, Schuh M, Erkan E. Downregulation of Akt induces proximal tubule epithelial cell apoptosis via Foxo-1-BIM pathway in proteinuric states. Res Sq. 2025. 10.1038/s41598-025-21498-1 PMC12568932 41152299

[67] Kumar PA, Welsh GI, Raghu G, Menon RK, Saleem MA, Reddy GB. Carboxymethyl lysine induces EMT in podocytes through transcription factor ZEB2: Implications for podocyte depletion and proteinuria in diabetes mellitus. Arch Biochem Biophys. 2016;590:10–9. doi: 10.1016/j.abb.2015.11.003 26550927

[68] Blagotinsek Cokan K, Urlep Z, Moskon M, Mraz M, Kong XY, Eskild W. Common Transcriptional Program of Liver Fibrosis in Mouse Genetic Models and Humans. Int J Mol Sci. 2021;22(2). 10.3390/ijms22020832 PMC7830925 33467660

[69] Ren N, Wang W-F, Zou L, Zhao Y-L, Miao H, Zhao Y-Y. The nuclear factor kappa B signaling pathway is a master regulator of renal fibrosis. Front Pharmacol. 2024;14:1335094. doi: 10.3389/fphar.2023.1335094 38293668 PMC10824958

[70] Chang TT, Chen JW. The Role of Chemokines and Chemokine Receptors in Diabetic Nephropathy. Int J Mol Sci. 2020;21(9). 10.3390/ijms21093172 PMC7246426 32365893

[71] Marozzi M, Parnigoni A, Negri A, Viola M, Vigetti D, Passi A. Inflammation, Extracellular Matrix Remodeling, and Proteostasis in Tumor Microenvironment. Int J Mol Sci. 2021;22(15). 10.3390/ijms22158102 PMC8346982 34360868

[72] Mou X, Leeman SM, Roye Y, Miller C, Musah S. Fenestrated Endothelial Cells across Organs: Insights into Kidney Function and Disease. Int J Mol Sci. 2024;25(16). 10.3390/ijms25169107 PMC11354928 39201792

[73] Ma T, Li X, Zhu Y, Yu S, Liu T, Zhang X, et al. Excessive Activation of Notch Signaling in Macrophages Promote Kidney Inflammation, Fibrosis, and Necroptosis. Front Immunol. 2022;13:835879. doi: 10.3389/fimmu.2022.835879 35280997 PMC8913942

[74] Kong L, Kong L, Li P, Gao L, Ma H, Shi B. Tribbles pseudokinase 3 promoted renal fibrosis by regulating the expression of DNA damage-inducible transcript 3 in diabetic nephropathy. Biomol Biomed. 2024;24(6):1559–70. doi: 10.17305/bb.2024.10419 38733632 PMC11496876

[75] Ma L-L, Bai Y, Liu W-H, Diao Z-L. Bioinformatics analysis of potential key ferroptosis-related genes involved in tubulointerstitial injury in patients with diabetic nephropathy. Ren Fail. 2023;45(1):2199095. doi: 10.1080/0886022X.2023.2199095 37038746 PMC10101677

[76] Xie M, Xie R, Huang P, Yap DYH, Wu P. GADD45A and GADD45B as novel biomarkers associated with chromatin regulators in renal ischemia-reperfusion injury. Int J Mol Sci. 2023;24(14). 10.3390/ijms241411304 PMC10379085 37511062

[77] Zhang X, Chao P, Zhang L, Xu L, Cui X, Wang S, et al. Single-cell RNA and transcriptome sequencing profiles identify immune-associated key genes in the development of diabetic kidney disease. Front Immunol. 2023;14:1030198. doi: 10.3389/fimmu.2023.1030198 37063851 PMC10091903

[78] Chen Y, Liu X, Shengbu M, Shi Q, Jiaqiu S, Lai X. Biomarkers: New Advances in Diabetic Nephropathy. Natural Product Communications. 2025;20(2):1934578X251321758.

[79] Andersen P, Uosaki H, Shenje LT, Kwon C. Non-canonical Notch signaling: emerging role and mechanism. Trends Cell Biol. 2012;22(5):257–65. doi: 10.1016/j.tcb.2012.02.003 22397947 PMC3348455

[80] Caliceti C, Nigro P, Rizzo P, Ferrari R. ROS, Notch, and Wnt signaling pathways: crosstalk between three major regulators of cardiovascular biology. Biomed Res Int. 2014;2014:318714. doi: 10.1155/2014/318714 24689035 PMC3932294

[81] Wang J, Fu D, Senouthai S, You Y. Critical roles of PI3K/Akt/NF‑κB survival axis in angiotensin II‑induced podocyte injury. Mol Med Rep. 2019;20(6):5134–44. doi: 10.3892/mmr.2019.10733 31638199 PMC6854545

[82] Wang X-M, Yao M, Liu S-X, Hao J, Liu Q-J, Gao F. Interplay between the Notch and PI3K/Akt pathways in high glucose-induced podocyte apoptosis. Am J Physiol Renal Physiol. 2014;306(2):F205-13. doi: 10.1152/ajprenal.90005.2013 24226527

