# Supplementary material for: Gene regulatory networks involved in activation of Notch signaling by AGEs in the pathogenesis of diabetic kidney disease
Source: PLoS One. 2026 Jan 2;21(1):e0335768. doi: 10.1371/journal.pone.0335768 (PMC12758727; doi:10.1371/journal.pone.0335768)
Supplement: S1 File — S1 Table. List of up and down regulated genes from GSE 30122. S2 Table. KEGG Pathways associated with DEGs. S3 Table. Hallmarks in the dataset obtained from GSE analysis. S4 Table. Trend for logFC within the existing data and independent data from KPMP Database. (ZIP) [file pone.0335768.s001.zip › S1 Table.docx]

| Down regulated genes | Up regulated genes |
| --- | --- |
| AVPI1 | LTF |
| FGF9 | CD1C |
| HRG | CXCL6 |
| ERVMER34-1 | MMP7 |
| EFHD1 | CD163 |
| LPL | MS4A6A |
| BAIAP2 | MS4A4A |
| GADD45B | RNASE6 |
| SYN1 | IGJ |
| ACACB | CD53 |
| BCAM | C1QB |
| AAMDC | CD48 |
| NELL1 | CPA3 |
| COPZ2 | TRIM16 |
| SLC46A3 | ARL4C |
| CEL | FCER1A |
| PCDH9 | COL1A2 |
| OPCML | SERPINA3 |
| EGF | EVI2A |
| USP2 | CASP1 |
| ITIH2 | C1QA |
| BMP7 | RARRES1 |
| ARHGEF17 | ITGB2 |
| KLF9 | C3 |
| ING2 | VSIG4 |
| VAMP2 | TNC |
| FKBP5 | VCAN |
| APOLD1 | TRBC1 |
| GNA13 | IRF8 |
| TYRP1 | COL6A3 |
| SLC6A2 | WIPF1 |
| NGF | NLGN4X |
| THRA | THBS2 |
| ETNPPL | PSTPIP2 |
| SLC31A2 | LY86 |
| TCF15 | FN1 |
| GHR | LAPTM5 |
| NPR2 | TNFAIP8 |
| TEX261 | MARCKS |
| B3GNT1 | GZMA |
| IQSEC2 | IL10RA |
| KBTBD11 | CCR2 |
| BTNL8 | CDC20 |
| CHCHD7 | CD2 |
| DNAJB12 | RRM2 |
| RABL3 | EZH2 |
| PRSS53 | LUM |
| GNAO1 | DYRK2 |
| PIGV | CTSS |
| SPTB | CLU |
| KLHL3 | PDLIM1 |
| ENO3 | KLRB1 |
| SIK3 | PLK2 |
| FBXO9 | AGR2 |
| KCTD13 | MARCKSL1 |
| DENND1A | MOXD1 |
| TAF15 | PPAP2C |
| EPB41L4A | TLR1 |
| PMS2P4 | HN1 |
| MYOZ1 | ACKR4 |
| WNT10B | QPCT |
| XPA | MRC1 |
| FRMD1 | GABRP |
| CCBL1 | LCK |
| ZSWIM1 | DOCK2 |
| FOXO4 | GPR65 |
| GOLIM4 | WFDC2 |
| SH3BGR | CORO1A |
| NR4A3 | TDO2 |
| FXR2 | TAC1 |
| ZNF91 | C3AR1 |
| LINC00472 | FAS |
| EFCC1 | FCGR2B |
| ZNF204P | FYB |
| ALOX12 | TLR7 |
| RPA3 | IL7R |
| UBAP2 | CD247 |
| C9orf9 | CDH6 |
| PCGF1 | COL15A1 |
| NSUN3 | ACTN1 |
| RGS7 | ALOX5 |
| KLHL21 | APOBEC3B |
| ZKSCAN3 | EVI2B |
| PIP5K1C | ACKR1 |
| GTF3C1 | TGFBI |
| MB | GZMK |
| PLXNA2 | ALDH1A3 |
| FAXDC2 | RARRES3 |
| JADE1 | MELK |
| CSPP1 | SERPINH1 |
| TSPYL2 | CLEC4A |
| MXI1 | CDH11 |
| PDE1B | FAM110B |
| WT1-AS | CSF1R |
| MTSS1 | CCL19 |
| TXNRD2 | HLA-DPA1 |
| SNAP29 | CELF2 |
| SOWAHC | MYD88 |
| C11orf71 | TYROBP |
| FBXO21 | ITGAM |
| EPN3 | CYBB |
| CDKL3 | SERPINE2 |
| KIAA1644 | TMPRSS4 |
| LTK | ALOX5AP |
| DXO | KIAA0226L |
| GPRASP1 | TES |
| TEX11 | TNFAIP2 |
| TNNC2 | CD3D |
| ATAT1 | CD44 |
| SSBP3 | PPIC |
| MPP3 | TOP2A |
| AL590762.11 | PLA2G4A |
| NFE2L1 | LAMC2 |
| TCEAL2 | TLR2 |
| ST3GAL4 | LMNB2 |
| RXRA | C7 |
| TEAD3 | LYZ |
| AKAP3 | CCR7 |
| ZBTB16 | IL33 |
| PAF1 | CFB |
| LONRF3 | PFN1 |
| FAM131A | FMOD |
| C11orf63 | CD52 |
| PPP1R15A | CST6 |
| MAGI2 | PTPRC |
| CACNB2 | DHRS9 |
| G0S2 | GCNT3 |
| TM6SF2 | ENAH |
| ZNF10 | ADRA2A |
| KLK1 | TMEM243 |
| IGFALS | IGSF6 |
| JAKMIP2 | REG1A |
| NPEPL1 | LYPD1 |
| CLTB | GPR18 |
| CDA | P2RY14 |
| PKI55 | TNFAIP6 |
| DNMT3L | IGLC1 |
| VEGFA | STK10 |
| RNF8 | CHI3L2 |
| HIPK2 | HLA-DMA |
| ASB9 | DIRAS2 |
| MPDZ | Mar-01 |
| TACR1 | CLEC10A |
| CXXC4 | PROM1 |
| COPG2IT1 | ADRB2 |
| THTPA | PRRC1 |
| CDC42BPA | KCNJ8 |
| C11orf24 | CCL5 |
| CES3 | STAB1 |
| MAPRE3 | CCL2 |
| MCUR1 | SYK |
| MINK1 | PLSCR1 |
| C1orf21 | GLIPR1 |
| VPS52 | SAMHD1 |
| GNA12 | PYCARD |
| ADAM30 | SOX4 |
| PKNOX2 | CD36 |
| MS4A12 | LTB |
| APOH | COL5A2 |
| KCNMB2 | VWF |
| FAM189A2 | PPIP5K1 |
| FLT1 | CDCP1 |
| NPIPA1 | VTCN1 |
| AASS | PROCR |
| CTNNBIP1 | ADAMTSL3 |
| GRIP2 | DACT1 |
| RASSF8 | IGFBP6 |
| SIRT4 | RP11-209A2.1 |
| SAP18 | DHCR24 |
| CTF1 | ST14 |
| DHRS3 | BLNK |
| C3orf18 | ARPC1B |
| EXPH5 | ITGA4 |
| HPGD | HLA-DRA |
| NEBL | GPR171 |
| MYOG | PTPRE |
| PHYHIP | FCER1G |
| CNTNAP2 | SERPINF1 |
| DUSP13 | SEL1L3 |
| ZNF224 | CD1D |
| PRX | CHST15 |
| GJA8 | GPR183 |
| MAN2A2 | IFNGR2 |
| UMOD | LPAR1 |
| SLMO1 | MSN |
| NDUFC1 | AHNAK2 |
| TMEM214 | ITM2C |
| PHLPP1 | DERL1 |
| ZNF385D | HPGDS |
| WWOX | TNFSF15 |
| BRIX1 | SLC1A3 |
| CTC-338M12.4 | FOLR2 |
| NXF3 | DCLK1 |
| FAM127A | PCID2 |
| TCL6 | PLAC8 |
| MUT | SVEP1 |
| SOX15 | PDIA4 |
| ADCY8 | BAZ1A |
| MYLPF | HNRNPAB |
| CA10 | COMP |
| MPC1 | IGKC |
| MMP28 | GALNT1 |
| TCP10 | HOPX |
| TNNI1 | CAPG |
| LRPAP1 | IRF9 |
| SYNJ2 | TMEM248 |
| DIP2C | TPD52L1 |
| G6PC | CAPN1 |
| IGFBP1 | RAC2 |
| LOC399491 | FMO3 |
| PIK3CB | MPZL1 |
| SNTA1 | HCLS1 |
| SPRR3 | RUNX3 |
| TACC2 | C2 |
| PCGF2 | LYN |
| ATP1B2 | POTEKP |
| GADD45G | TRAT1 |
| DLG2 | PRKX |
| ZNF124 | KRT19 |
| SLC6A16 | IGHM |
| DLEC1 | TPM1 |
| TRIM17 | SLAMF1 |
| CHST8 | CYB5R4 |
| PTPN14 | KIF20A |
| RBP4 | LASP1 |
| POFUT2 | GBP2 |
| PIGK | ADH1B |
| COL9A1 | FCGR1B |
| NPHS1 | ALDH18A1 |
| MAPK8 | HS3ST1 |
| SOX13 | INPP5D |
| HIF3A | FJX1 |
| PBOV1 | M6PR |
| APLP1 | GDI2 |
| ALS2CL | TKT |
| SYDE1 | ZWILCH |
| FAM110D | LPXN |
| ANKRD28 | PRKCB |
| FAM134B | HDAC9 |
| GGTLC2 | CXCR4 |
| HSPA1L | LAT |
| LOC101927051 | CASP3 |
| GOLGB1 | FZD1 |
| DCBLD2 | TRAF3IP2 |
| GML | CLEC7A |
| CGGBP1 | CX3CR1 |
| PBX2 | CD86 |
| MKRN3 | XPNPEP1 |
| GAD1 | KRT7 |
| SGSM2 | NBEAL2 |
| NTNG1 | NAA40 |
| ARSF | TSPAN13 |
| HIST1H4E | TNFRSF1B |
| CCDC6 | RBBP4 |
| VPRBP | ITK |
| RBFOX1 | SLA |
| SALL1 | BIRC3 |
| NLK | NRP1 |
| DYNLT3 | CASP8 |
| SPINT3 | LAMP3 |
| SYT2 | NCF4 |
| BACE1 | MID1 |
| TET3 | COL3A1 |
| KCNN2 | RAP2B |
| CTSV | LEF1 |
| DUSP1 | PLEK |
| GPR144 | CCNB1 |
| MAGEL2 | DTL |
| WDTC1 | PID1 |
| SST | IDO1 |
| DYRK3 | KIF18B |
| FOSB | MPZL2 |
| TNPO2 | PRR5L |
| MTHFD2L | GUCY1A3 |
| POLR2C | IGLV1-44 |
| F11 | FUT4 |
| RALYL | CLDN4 |
| GLI1 | NCK1 |
| CNNM2 | TMEM158 |
| CGRRF1 | CKS1B |
| CDH10 | IMPDH2 |
| GYG2 | CD27 |
| MRPL46 | LY96 |
| ADAM11 | NEU1 |
| SMAD6 | RASSF2 |
| C21orf2 | TUBA1B |
| PLEKHB1 | ADAM10 |
| VPS37C | TINF2 |
| CENPJ | TYMP |
| FBXL7 | DOCK10 |
| APOD | THOC6 |
| TRIM8 | VCAM1 |
| MLLT4-AS1 | SELL |
| MLX | HAVCR1 |
| CA9 | STK39 |
| TAS2R16 | FKBP11 |
| NPRL2 | MICAL1 |
| ZC2HC1A | P2RY13 |
| RBM41 | UPP1 |
| CYP4F12 | RBM15B |
| MYOZ2 | SPP1 |
| ELK2AP | PCOLCE |
| SLC1A6 | HLA-DQB2 |
| CCDC19 | CASP6 |
| LSM14B | LCP1 |
| HAAO | MAP3K7CL |
| GS1-164F24.1 | GGT5 |
| RP3-406C18.2 | RPS3 |
| NDRG1 | C19orf10 |
| ARF3 | NMI |
| U2AF2 | IGLL3P |
| NAB2 | TNFSF10 |
| AOC1 | VWA5A |
| B3GALT2 | GPX1 |
| CDC14A | MAP7D3 |
| SEMA4G | ENO1 |
| SERPINI2 | CORO1C |
| PCDHB12 | MMD |
| MPP5 | ITGA2 |
| PEG3 | CTSG |
| CYP3A4 | P2RX4 |
| KLK8 | TRAF3IP3 |
| TBXA2R | ARF5 |
| MRPL49 | IL7 |
| CYP27B1 | TRIL |
| SH3BP1 | NINJ2 |
| ZNF358 | CYTIP |
| MID2 | CASK |
| BAZ1B | NT5DC2 |
| HEY1 | SLC35F2 |
| GAS2L1 | CAPZA1 |
| IKZF4 | SFRP1 |
| PTCH1 | RTN1 |
| SEC62 | RASA2 |
| SIRT2 | ZNF217 |
| TNNT2 | MFAP2 |
| FZD9 | KDELC1 |
| FLG | SSR2 |
| EZH1 | MAP4K1 |
| CPEB1 | LIG1 |
| MROH7 | SNW1 |
| CLCA1 | IRF2 |
| RND1 | TOR4A |
| TYRO3 | CYP24A1 |
| CDIPT | PDGFRA |
| LY6G5C | SMARCC1 |
| LIM2 | THEMIS2 |
| LGSN | NCF1 |
| GADD45A | FCGR2A |
| PLXNA3 | SRPK1 |
| SUN2 | MAPK13 |
| HEXIM1 | OTUB2 |
| APOC3 | NUP93 |
| DSCAM | MON1B |
| CAMK2G | CD28 |
| WISP3 | HLA-DMB |
| MARK4 | YWHAH |
| LDB1 | LAMA2 |
| TSC22D3 | HABP2 |
| MAPK10 | AOAH |
| ZFYVE9 | RHOH |
| BFSP1 | ISG20 |
| RING1 | TRIM22 |
| EPS15L1 | PTEN |
| GRIK4 | NMU |
| TMEM80 | RCN1 |
| UPK1A | CDH3 |
| PEX14 | BZW2 |
| IRS2 | HLA-DPB1 |
| ENDOU | RP5-1118D24.2 |
| GRAMD1B | TRIM38 |
| CTB-102L5.7 | TACC3 |
| ANXA2P1 | RP11-116O18.1 |
| ZFP2 | PPIB |
| IVD | SORBS2 |
| CYP11B2 | ACTB |
| HSPE1 | CD24 |
| METTL1 | FBLN5 |
| ALOX12B | NFKBIE |
| DPP6 | TNMD |
| THSD7A | SIDT1 |
| RRNAD1 | TSPAN1 |
| CD83 | VAV1 |
| THY1 | EPB41L2 |
| ZNF580 | PLXDC1 |
| UQCRQ | RPA2 |
| ZNF562 | SLC35D2 |
| MAP6D1 | ECT2 |
| PPIG | GPR64 |
| FEV | MDK |
| ERF | CRTAM |
| HSPB3 | ANXA3 |
| SNED1 | TTC39A |
| ELK1 | SIGLEC1 |
| OCA2 | MUC1 |
| HGH1 | ARNTL2 |
| SEMA6A | FBN1 |
| PRDM4 | CXCL1 |
| PRKG1 | RGS13 |
| LOC730101 | CENPU |
| HOXC8 | BORA |
| ERC2-IT1 | SASH3 |
| ANKRD26 | STT3A |
| BAG3 | HNRNPL |
| KIR2DS5 | SAMSN1 |
| OCEL1 | KDELR2 |
| ZNF35 | AHR |
| BPY2 | LDLRAP1 |
| UNC93A | SETBP1 |
| TPPP3 | RFC2 |
| MAPK1IP1L | IRGQ |
| NPR3 | AEBP1 |
| FOXC1 | APBA2 |
| ZNF614 | ACSL4 |
| MRPS22 | SLC44A4 |
| SSX3 | NCF2 |
| RIMS1 | SIAH2 |
| HMCES | KRT8 |
| ANAPC13 | SYT11 |
| SCUBE3 | ADAMDEC1 |
| CTDSPL | GINS1 |
| EFNA1 | C10orf2 |
| LRRC6 | FCN1 |
| PMF1 | SUPT16H |
| TFE3 | RAB5B |
| PPP1R3D | EHF |
| GPC5 | TUBA1C |
| KCND3 | ARSJ |
| PCDHB3 | LCN2 |
| TRIP10 | R3HDM4 |
| HSPA12A | TUBB |
| PXMP4 | RAB38 |
| FERMT2 | ZNF227 |
| MYL10 | CCR5 |
| TIMM9 | FANCG |
| FNDC8 | GYPC |
| PPP1R2P9 | CFLAR |
| FKBP8 | MFAP4 |
| NIN | SPON1 |
| CDX2 | TNIK |
| LSM3 | CCR6 |
| GALC | STK19 |
| NGFRAP1 | PTPRF |
| TRMT44 | GALNT2 |
| C21orf62 | CRIP1 |
| DMRT1 | CTSK |
| TCL1B | DOK5 |
| GABARAPL3 | SRPX |
| SIRPB1 | TAGLN2 |
| HBEGF | ZKSCAN4 |
| LOC100289473 | SNF8 |
| EGFL7 | HGF |
| IKBKG | HLA-J |
| KIAA1467 | SELPLG |
| TAF1C | CKLF |
| LOC100506469 | PARP1 |
| FOXE3 | IL32 |
| ADCY1 | CEMIP |
| PRRC2A | SSPN |
| ARC | LAMC1 |
| PLCG2 | ENTPD1 |
| AMOT | RPS6KA3 |
| GRIK2 | GSPT1 |
| NUDT7 | CXCR6 |
| NUP88 | SPTLC2 |
| C1orf50 | ZW10 |
| ADD3-AS1 | NAA15 |
| FAT2 | CEP55 |
| ZNF274 | PUS1 |
| CNTN1 | TESC |
| TGFBR3 | BCR |
| CCDC25 | CD63 |
| MYOZ3 | NXT1 |
| PRSS3P2 | C1RL |
| C12orf29 | TNFRSF17 |
| CAMK2A | VAT1 |
| PDE4DIP | STX18 |
| RUSC1 | XRCC5 |
| STEAP3 | MILR1 |
| PTPRD | ST3GAL5 |
| KIAA0040 | FAM57A |
| DDIT3 | HARS2 |
| CYP11A1 | ARL6IP5 |
| MYF5 | NPC2 |
| SLC26A4 | CCND2 |
| LMNA | CPVL |
| ST3GAL6 | LST1 |
| MYO9A | GNA15 |
| SORT1 | TFPI2 |
| SPHK2 | PF4V1 |
| OR1A2 | TXLNA |
| RITA1 | RNF34 |
| CREM | XBP1 |
| STAC | SFRP4 |
| OTOR | FHL2 |
| SMARCD1 | MCM3 |
| ARHGAP28 | CRISPLD2 |
| PAK4 | COL5A1 |
| MNAT1 | MRPL11 |
| SNRNP35 | TMSB10 |
| CBX8 | GMIP |
| SOCS2 | LPGAT1 |
| SALL2 | PLEKHA1 |
| PLCL1 | DNMT1 |
| ATOX1 | PFKP |
| PCNT | COL16A1 |
| SERPIND1 | LAPTM4A |
| ST3GAL1 | WDR1 |
| FAM184A | BCL11A |
| PROC | PPL |
| ADM | NARF |
| PEX26 | LBR |
| PDZD2 | PREB |
| SPSB1 | C7orf49 |
| SMEK2 | C1S |
| KANK3 | SNX10 |
| ELOVL4 | GPR56 |
| MECP2 | PAFAH1B3 |
| HS3ST3A1 | RPAP3 |
| GUCA2A | MCM2 |
| PART1 | EDNRB |
| HSF2BP | SAYSD1 |
| ZCWPW1 | FANCI |
| MARK1 | C4orf19 |
| ATP9B | OLFM4 |
| APBB2 | DRAM1 |
| MPPE1 | ADA |
| WDR83OS | CD8A |
| PDLIM2 | SPINT1 |
| NOL3 | JAK1 |
| MOB4 | NIP7 |
| NDUFB8 | SELT |
| CCSER2 | MYC |
| RNF32 | MNS1 |
| C6orf123 | KCTD5 |
| STX8 | CHST7 |
| TAL1 | IGFLR1 |
| ESM1 | KLF8 |
| RDX | HCAR3 |
| LRRC75B | PVRIG |
| ZNF226 | PYGL |
| CRYGB | ITPA |
| LRFN4 | ABCC3 |
| FBXO22 | COL4A1 |
| AGPAT3 | TRRAP |
| OR51E2 | CLC |
| MME | ANXA4 |
| RBKS | PREP |
| SLC6A4 | PLA2G7 |
| EXOC3 | PCGF3 |
| STARD8 | RBL1 |
| LRRC2 | CFD |
| SPTAN1 | MCAM |
| PURA | FXYD5 |
| BEST1 | DLGAP5 |
| DNAH9 | EEF1A2 |
| COX7A1 | PTPRCAP |
| CTNS | TNFRSF1A |
| MOSPD3 | RUVBL1 |
| BST1 | LINC01140 |
| DKKL1 | LIPG |
| ROM1 | KIF11 |
| KNG1 | ITGB7 |
| ZNF428 | SMPD2 |
| CHPF | OLFML3 |
| ZNF16 | LRRC16A |
| C14orf132 | HMBS |
| CUX1 | CD209 |
| GTF3C4 | CARS2 |
| SNAPC2 | PPIF |
| NLGN3 | HCK |
| TRPM6 | AF070581 |
| NSFL1C | B2M |
| GRIA1 | THG1L |
| WNT5B | MICALL2 |
| FBXL2 | CD14 |
| AP4S1 | PAPPA2 |
| PVRL2 | NUS1P3 |
| RUSC2 | SFN |
| NHLH1 | CCNB2 |
| DACH1 | PRAME |
| GSTA3 | PITPNM1 |
| PSG6 | AMPD1 |
| TCEB3-AS1 | HMGB3 |
| KPNA6 | ANKLE2 |
| PRORY | NUP85 |
| SNAI1 | XCL1 |
| KCNA2 | RAD51AP1 |
| DNAJC6 | CLNS1A |
| CEP63 | IL18 |
| ATP6V1G2 | GPR143 |
| GLP2R | SCGN |
| CYP3A43 | MYO1F |
| KCNJ13 | TUBGCP5 |
| MGAT5 | AC004692.5 |
| KIR2DL5A | EXT1 |
| EPB41L5 | BMP2K |
| ZNF195 | BASP1 |
| ALOX15B | TSR1 |
| VWA7 | RPS5 |
| ATF7 | TAF12 |
| CLIP3 | HELLS |
| GPX5 | UCP2 |
| HSD17B1 | KNTC1 |
| PIGL | PILRA |
| CDK5RAP1 | EFNA4 |
| CCDC186 | RNPEP |
| MIER2 | MYBL1 |
| CRYGA | PTP4A2 |
| TSKU | SH2D1A |
| PCYOX1L | ICOS |
| TPPP | TRMT2B |
| DCAF8 | ADCY6 |
| FTSJ3 | ICAM3 |
| REXO2 | GFRA1 |
| KLF7 | IGLL5 |
| C11orf95 | TUBA1A |
| CYB5R3 | CD74 |
| CELF3 | MVP |
| EIF2D | CENPM |
| HSD17B12 | IRAK1 |
| ALDH1A2 | GAL3ST4 |
| GRM1 | BCL11B |
| NOP2 | PBK |
| LIMS2 | C4BPA |
| SEC31A | LOC101929272 |
| COX6C | BHLHE41 |
| RALGAPA1 | DIEXF |
| ZNF444 | CHEK1 |
| FLJ10038 | SET |
| GP2 | HNRNPF |
| SLC12A5 | BMP5 |
| SCAPER | COL14A1 |
| ZNF415 | ZNF207 |
| SARM1 | TTF2 |
| LMF1 | CEBPA |
| GTPBP1 | MET |
| LRP4 | STAT4 |
| MAPK8IP2 | EIF4A3 |
| GTF3C2 | BTK |
| DNAJC8 | FGF7 |
| EFNB1 | HNRNPU |
| YIPF4 | SNN |
| DDN | SSR1 |
| FXR1 | UBE2J1 |
| CAPN9 | BCL10 |
| TRPC6 | DESI1 |
| USP49 | NELL2 |
| KIR2DL2 | AGPS |
| SH3YL1 | CCNE1 |
| IL37 | UAP1L1 |
| KCNQ1DN | HOMER3 |
| QPCTL | RPS6KA1 |
| KRT2 | DKK3 |
| ADCYAP1R1 | IFI35 |
| RPGR | LAMB3 |
| SOCS7 | SSH1 |
| FBXO42 | PRKAB1 |
| TENC1 | GPX7 |
| FGF4 | FAM69A |
|  | TNFRSF11B |
|  | ZWINT |
|  | ADAM12 |
|  | ALDH1A1 |
|  | GALNT7 |
|  | LCP2 |
|  | SLAMF7 |
|  | ZNF107 |
|  | C9orf91 |
|  | ITGAX |
|  | SP3 |
|  | RYK |
|  | NADK |
|  | ITGAL |
|  | ACTR1A |
|  | FAIM3 |
|  | GPR126 |
|  | TRAM1 |
|  | MSL1 |
|  | FAM49A |
|  | ST5 |
|  | DONSON |
|  | ATM |
|  | OLFML2B |
|  | WNT2B |
|  | CACNA2D3 |
|  | TRAC |
|  | EDN1 |
|  | GATC |
|  | C10orf10 |
|  | ZAP70 |
|  | SOAT1 |
|  | PDIA6 |
|  | HIF1A |
|  | REC8 |
|  | PXDN |
|  | MMP2 |
|  | GCN1L1 |
|  | OCLN |
|  | NCOA3 |
|  | EHBP1L1 |
|  | FGG |
|  | MPG |
|  | MEOX1 |
|  | TAGLN |
|  | PALB2 |
|  | ARPC3 |
|  | FLVCR2 |
|  | CD3G |
|  | NUP210 |
|  | MYO5A |
|  | DFFB |
|  | GREM1 |
|  | MNDA |
|  | LRIG2 |
|  | COL8A2 |
|  | CXCL12 |
|  | KAL1 |
|  | TRO |
|  | TMEM62 |
|  | LSM2 |
|  | C1QTNF3 |
|  | CARHSP1 |
|  | LIMA1 |
|  | TMSB4Y |
|  | GIT2 |
|  | TOMM34 |
|  | SOX9 |
|  | SLPI |
|  | ABCA8 |
|  | HLA-B |
|  | ARL4A |
|  | KIAA0753 |
|  | SEC24C |
|  | CLN3 |
|  | CD300A |
|  | MALT1 |
|  | ABHD2 |
|  | THADA |
|  | PSMB10 |
|  | BICC1 |
|  | FAM155A |
|  | CD38 |
|  | ARMC7 |
|  | FAM129A |
|  | IREB2 |
|  | SLC34A2 |
|  | SLC22A3 |
|  | ADAM28 |
|  | IL21R |
|  | PLCB4 |
|  | WDR77 |
|  | MDN1 |
|  | AKR1B1 |
|  | SUPT20H |
|  | GPC4 |
|  | IFNAR2 |
|  | TRIM21 |
|  | MRPL18 |
|  | SPHK1 |
|  | CLDN7 |
|  | BUB1B |
|  | CASP5 |
|  | GFPT1 |
|  | ELOVL6 |
|  | PAK6 |
|  | THYN1 |
|  | SRGN |
|  | DENND5A |
|  | MYRF |
|  | AKR1B10 |
|  | HLA-F |
|  | ARHGAP26 |
|  | PRKAG1 |
|  | MREG |
|  | LAMB1 |
|  | HLA-G |
|  | RNFT2 |
|  | EDC4 |
|  | HTR2B |
|  | INPP4B |
|  | SLC38A1 |
|  | SRBD1 |
|  | LHX6 |
|  | GINS2 |
|  | FLT3LG |
|  | SEC11A |
|  | RPS6 |
|  | PAK2 |
|  | BCL2A1 |
|  | LDLRAD4 |
|  | ZNF652 |
|  | PHLDA2 |
|  | LHFPL2 |
|  | MAN2B1 |
|  | NUP155 |
|  | C11orf80 |
|  | MITF |
|  | ABLIM1 |
|  | TMEM123 |
|  | CSTA |
|  | VDAC1 |
|  | RPL39 |
|  | TSPO |
|  | INTS5 |
|  | IFI16 |
|  | PYGB |
|  | HLA-DOB |
|  | INHBA |
|  | TTK |
|  | HLA-DQA1 |
|  | CEP57 |
|  | RBM5 |
|  | ACTR3 |
|  | SETX |
|  | UBAC1 |
|  | RPP30 |
|  | CTBP1 |
|  | UBASH3A |
|  | ARHGDIB |
|  | CHEK2 |
|  | ACVR2A |
|  | FGL2 |
|  | NPY5R |
|  | CDC25B |
|  | MSR1 |
|  | MCM5 |
|  | NFYC |
|  | CAPN6 |
|  | PEX6 |
|  | CD69 |
|  | SPATS2 |
|  | RALGPS2 |
|  | CPE |
|  | FADS3 |
|  | INTS9 |
|  | LMCD1 |
|  | Sep-06 |
|  | E2F5 |
|  | STAT3 |
|  | CTSD |
|  | IL2RB |
|  | OGFRL1 |
|  | VASH2 |
|  | CD160 |
|  | EMR2 |
|  | CDK4 |
|  | PLS1 |
|  | CD40 |
|  | DHX38 |
|  | GLOD4 |
|  | LINC00963 |
|  | IGLJ3 |
|  | MORF4L2 |
|  | SPAG4 |
|  | LPIN2 |
|  | APMAP |
|  | CARD8 |
|  | STIL |
|  | EGLN3 |
|  | GANAB |
|  | PPP1R14B |
|  | NBL1 |
|  | MYL12B |
|  | FAM169A |
|  | ATIC |
|  | RTN3 |
|  | PSTPIP1 |
|  | PSMB8 |
|  | PASK |
|  | PRCC |
|  | WBSCR16 |
|  | LAX1 |
|  | SCIN |
|  | ARFGAP1 |
|  | CH25H |
|  | SH2D3A |
|  | EFHD2 |
|  | PTPN3 |
|  | LLGL2 |
|  | CYTH4 |
|  | IRF1 |
|  | PRKD2 |
|  | COL6A2 |
|  | PTTG1 |
|  | MCM4 |
|  | CXCL8 |
|  | CTNNB1 |
|  | CSTF1 |
|  | MFSD10 |
|  | BID |
|  | NR2F1 |
|  | KIAA0930 |
|  | RGS19 |
|  | TOX |
|  | HLA-C |
|  | NAP1L4 |
|  | CCL18 |
|  | RPS19 |
|  | USP20 |
|  | CBFA2T3 |
|  | P2RY10 |
|  | LSM12 |
|  | SDF2L1 |
|  | SPAG1 |
|  | IFIT2 |
|  | B4GALT5 |
|  | CD96 |
|  | HTRA2 |
|  | IFNGR1 |
|  | TRIB3 |
|  | B3GNTL1 |
|  | FLOT2 |
|  | TPP2 |
|  | ZNF22 |
|  | F2RL1 |
|  | SAE1 |
|  | LMAN2 |
|  | RAB25 |
|  | RPS12 |
|  | PPP1R8 |
|  | RWDD2A |
|  | TBCCD1 |
|  | C6 |
|  | NOD2 |
|  | LPCAT3 |
|  | HCP5 |
|  | MYO6 |
|  | IL16 |
|  | SMC6 |
|  | MZB1 |
|  | BCLAF1 |
|  | ZNF318 |
|  | PGAP2 |
|  | CHL1 |
|  | H2AFY |
|  | CFI |
|  | CLDN3 |
|  | ZFYVE21 |
|  | UBR4 |
|  | ZFP69B |
|  | PRC1 |
|  | GRB7 |
|  | CCL21 |
|  | PRUNE |
|  | LAPTM4B |
|  | GZMB |
|  | LRMP |
|  | FLII |
|  | C1orf116 |
|  | CACNA1D |
|  | PUS7 |
|  | RAE1 |
|  | HNRNPK |
|  | STK24 |
|  | OGDH |
|  | RNF125 |
|  | ILF3 |
|  | HLA-DRB6 |
|  | S100A4 |
