# Supplementary material for: Gene regulatory networks involved in activation of Notch signaling by AGEs in the pathogenesis of diabetic kidney disease
Source: PLoS One. 2026 Jan 2;21(1):e0335768. doi: 10.1371/journal.pone.0335768 (PMC12758727; doi:10.1371/journal.pone.0335768)
Supplement: S1 File — S1 Table. List of up and down regulated genes from GSE 30122. S2 Table. KEGG Pathways associated with DEGs. S3 Table. Hallmarks in the dataset obtained from GSE analysis. S4 Table. Trend for logFC within the existing data and independent data from KPMP Database. (ZIP) [file pone.0335768.s001.zip › S2 Table .docx]

| **Description** | **GeneID** |
| --- | --- |
| PI3K-Akt signaling pathway | COL1A2/TNC/COL6A3/THBS2/FN1/FGF9/IL7R/CSF1R/LAMC2/TLR2/SYK/VWF/ITGA4/LPAR1/EGF/COMP/NGF/GHR/SPP1/ITGA2/IL7/PDGFRA/YWHAH/LAMA2/PTEN/RXRA/MAGI2/VEGFA/HGF/LAMC1/CCND2/FLT1/JAK1/MYC/PHLPP1/COL4A1/PIK3CB/ITGB7/COL9A1/EFNA4/MET/FGF7/CCNE1/LAMB3/IFNAR2/EFNA1/LAMB1/FLT3LG/RPS6/TCL1B/IKBKG/IL2RB/CDK4/COL6A2/FGF4 |
| Human papillomavirus infection | COL1A2/TNC/COL6A3/THBS2/FN1/FAS/LAMC2/VWF/ITGA4/EGF/COMP/IRF9/CASP3/FZD1/CASP8/SPP1/ITGA2/WNT10B/LAMA2/PTEN/VEGFA/LAMC1/CCND2/JAK1/COL4A1/RBL1/TNFRSF1A/PIK3CB/ITGB7/DLG2/COL9A1/CCNE1/LAMB3/ATM/WNT2B/HEY1/FZD9/HLA-B/IFNAR2/HLA-F/LAMB1/HLA-G/IKBKG/CDK4/LLGL2/IRF1/COL6A2/CTNNB1/HLA-C/WNT5B/ATP6V1G2/UBR4 |
| Epstein-Barr virus infection | FAS/CD247/HLA-DPA1/MYD88/CD3D/CD44/TLR2/HLA-DMA/GADD45B/SYK/BLNK/HLA-DRA/IRF9/RUNX3/LYN/CASP3/CASP8/SNW1/MAPK13/HLA-DMB/HLA-DPB1/NFKBIE/ENTPD1/CCND2/JAK1/MYC/PIK3CB/GADD45G/B2M/MAPK8/IRAK1/BTK/CCNE1/ITGAL/CD3G/GADD45A/MAPK10/HLA-B/IFNAR2/HLA-F/HLA-G/IKBKG/HLA-DOB/PLCG2/HLA-DQA1/STAT3/CDK4/CD40/BID/HLA-C |
| Cytokine-cytokine receptor interaction | CXCL6/IL10RA/CCR2/ACKR4/FAS/IL7R/CSF1R/CCL19/CCR7/IL33/CCL5/CCL2/LTB/IFNGR2/TNFSF15/BMP7/CXCR4/CX3CR1/TNFRSF1B/NGF/CD27/GHR/TNFSF10/IL7/CXCL1/CCR5/CCR6/IL32/CXCR6/TNFRSF17/PF4V1/CTF1/TNFRSF1A/XCL1/IL18/BMP5/TNFRSF11B/CXCL12/IL21R/IFNAR2/INHBA/ACVR2A/IL2RB/CD40/CXCL8/CCL18/IFNGR1/IL16/CCL21/IL37 |
| Tuberculosis | ITGB2/C3/IL10RA/CTSS/TLR1/MRC1/CORO1A/FCGR2B/HLA-DPA1/MYD88/ITGAM/TLR2/HLA-DMA/SYK/HLA-DRA/FCER1G/IFNGR2/CASP3/CLEC7A/CASP8/FCGR2A/MAPK13/HLA-DMB/HLA-DPB1/RAB5B/JAK1/TNFRSF1A/CD209/CD14/MAPK8/IL18/CD74/IRAK1/BCL10/ITGAX/CYP27B1/CAMK2G/MAPK10/MALT1/SPHK1/HLA-DOB/HLA-DQA1/CAMK2A/NFYC/CTSD/SPHK2/BID/IFNGR1/NOD2 |
| Human T-cell leukemia virus 1 infection | MMP7/ITGB2/CDC20/LCK/HLA-DPA1/CD3D/HLA-DMA/HLA-DRA/NRP1/HLA-DMB/PTEN/HLA-DPB1/CCND2/JAK1/MYC/ADCY8/TRRAP/TNFRSF1A/PIK3CB/B2M/MAPK8/CCNB2/ADCY6/CHEK1/CCNE1/ITGAL/ATM/CD3G/MAPK10/HLA-B/ELK1/BUB1B/ANAPC13/HLA-F/HLA-G/IKBKG/VDAC1/TSPO/ADCY1/HLA-DOB/HLA-DQA1/CHEK2/IL2RB/CDK4/CD40/PTTG1/HLA-C |
| MAPK signaling pathway | FGF9/FAS/CSF1R/MYD88/PLA2G4A/GADD45B/EGF/RAC2/PRKCB/CASP3/NGF/RASA2/MAP4K1/PDGFRA/MAPK13/CACNB2/VEGFA/HGF/RPS6KA3/GNA12/FLT1/MYC/TNFRSF1A/GADD45G/CD14/MAPK8/HSPA1L/EFNA4/IRAK1/NLK/DUSP1/MET/FGF7/RPS6KA1/CACNA2D3/GADD45A/MAPK10/ELK1/EFNA1/FLT3LG/PAK2/IKBKG/CDC25B/DDIT3/MAPK8IP2/CACNA1D/FGF4 |
| Salmonella infection | CASP1/MYD88/TLR2/PFN1/PTPRC/PYCARD/ARPC1B/AHNAK2/M6PR/CASP3/BIRC3/CASP8/LEF1/LY96/TUBA1B/RPS3/TNFSF10/MAPK13/RHOH/ACTB/RAB5B/TUBA1C/TUBB/MYC/TNFRSF1A/PIK3CB/CD14/MAPK8/IL18/TUBA1A/IRAK1/DYNLT3/ACTR1A/ARPC3/MAPK10/CASP5/MYL10/IKBKG/ACTR3/MYL12B/CYTH4/FBXO22/CXCL8/CTNNB1/MYO6 |
| Pathways of neurodegeneration - multiple diseases | FAS/CYBB/DERL1/CAPN1/PRKCB/CASP3/FZD1/TNFRSF1B/CASP8/TUBA1B/GPX1/WNT10B/MAPK13/TUBA1C/TUBB/XBP1/NDUFC1/TNFRSF1A/PPIF/MAPK8/TUBA1A/UBE2J1/GPX7/ACTR1A/WNT2B/FZD9/CAMK2G/MAPK10/UQCRQ/PLCB4/VDAC1/CAMK2A/DDIT3/NDUFB8/CTNNB1/BID/DNAH9/COX7A1/GRIA1/WNT5B/HTRA2/GPX5/GRM1/COX6C/CACNA1D |
| Focal adhesion | COL1A2/TNC/COL6A3/THBS2/FN1/ACTN1/LAMC2/VWF/ITGA4/EGF/COMP/RAC2/PRKCB/BIRC3/SPP1/ITGA2/PDGFRA/PIP5K1C/LAMA2/PTEN/ACTB/VAV1/VEGFA/HGF/LAMC1/CCND2/FLT1/COL4A1/PIK3CB/ITGB7/COL9A1/MAPK8/MET/LAMB3/MAPK10/ELK1/PAK6/LAMB1/MYL10/PAK2/PAK4/MYL12B/COL6A2/CTNNB1 |
| Regulation of actin cytoskeleton | ITGB2/FN1/FGF9/ACTN1/ITGAM/BAIAP2/C7/PFN1/ENAH/ARPC1B/ITGA4/LPAR1/MSN/EGF/RAC2/CXCR4/GNA13/ITGA2/PDGFRA/PIP5K1C/ACTB/VAV1/GNA12/PIK3CB/ITGB7/FGF7/SSH1/ITGAX/ITGAL/ARPC3/CXCL12/TMSB4Y/PAK6/MYL10/PAK2/ACTR3/PAK4/MYL12B/SCIN/RDX/KNG1/C6/FGF4 |
| Cell adhesion molecules | ITGB2/VCAN/NLGN4X/CD2/HLA-DPA1/ITGAM/PTPRC/HLA-DMA/VTCN1/ITGA4/HLA-DRA/CD86/CLDN4/VCAM1/SELL/CD28/HLA-DMB/CDH3/HLA-DPB1/SIGLEC1/PTPRF/SELPLG/CNTNAP2/CD8A/ITGB7/ICOS/ICAM3/NTNG1/ITGAL/OCLN/HLA-B/CLDN7/HLA-F/HLA-G/HLA-DOB/HLA-DQA1/CNTN1/PTPRD/CD40/HLA-C/NLGN3/CLDN3 |
| Pathogenic Escherichia coli infection | CASP1/WIPF1/FAS/MYD88/BAIAP2/PYCARD/ARPC1B/LPAR1/HCLS1/CASP3/GNA13/CASP8/CLDN4/NCK1/TUBA1B/RPS3/TNFSF10/FCGR2A/MAPK13/ACTB/TUBA1C/TUBB/GNA12/TNFRSF1A/MAPK8/IL18/MYO1F/TUBA1A/IRAK1/OCLN/ARPC3/MYO5A/MAPK10/SEC24C/CLDN7/PAK2/IKBKG/ACTR3/CYTH4/CXCL8/MYO6/CLDN3 |
| Coronavirus disease - COVID-19 | C1QB/CASP1/C1QA/C3/C3AR1/TLR7/MYD88/CYBB/TLR2/C7/CFB/CCL2/SYK/VWF/IRF9/C2/PRKCB/NRP1/RPS3/FCGR2A/MAPK13/C1S/JAK1/CFD/TNFRSF1A/PIK3CB/MAPK8/RPS5/IRAK1/FGG/MAPK10/IFNAR2/RPS6/HBEGF/IKBKG/RPL39/PLCG2/STAT3/CXCL8/RPS19/RPS12/C6 |
| Phagosome | ITGB2/C3/THBS2/CTSS/MRC1/CORO1A/FCGR2B/HLA-DPA1/ITGAM/CYBB/TLR2/HLA-DMA/CD36/HLA-DRA/COMP/M6PR/CLEC7A/NCF4/TUBA1B/ITGA2/NCF1/FCGR2A/HLA-DMB/HLA-DPB1/ACTB/NCF2/RAB5B/TUBA1C/TUBB/STX18/CD209/CD14/TUBA1A/HLA-B/HLA-F/HLA-G/HLA-DOB/HLA-DQA1/MSR1/HLA-C/ATP6V1G2 |
| Lipid and atherosclerosis | CASP1/FAS/MYD88/CYBB/TLR2/CCL5/CCL2/PYCARD/CD36/LYN/CASP3/CASP8/NCF4/LY96/VCAM1/CASP6/TNFSF10/NCF1/MAPK13/VAV1/CXCL1/NCF2/RXRA/XBP1/TNFRSF1A/PIK3CB/CD14/MAPK8/HSPA1L/IL18/IRAK1/CAMK2G/MAPK10/PLCB4/IKBKG/CAMK2A/DDIT3/STAT3/CD40/CXCL8/BID |
| Cytoskeleton in muscle cells | COL1A2/VCAN/COL6A3/THBS2/FN1/PDLIM1/LMNB2/COL5A2/ITGA4/COMP/TPM1/COL3A1/ENO3/ENO1/ITGA2/MYOZ1/CAPZA1/LAMA2/ACTB/FBN1/TNNC2/SSPN/FHL2/COL5A1/LBR/NEBL/COL4A1/TNNI1/SNTA1/ITGB7/ATP1B2/COL9A1/AMPD1/MYOZ2/TNNT2/SUN2/MYOZ3/LMNA/PDLIM2/COL6A2/SPTAN1 |
| Influenza A | CASP1/FAS/TLR7/HLA-DPA1/MYD88/TMPRSS4/IL33/HLA-DMA/CCL5/CCL2/PYCARD/HLA-DRA/IFNGR2/IRF9/PRKCB/CASP3/CASP8/TNFSF10/HLA-DMB/HLA-DPB1/ACTB/NXT1/JAK1/NXF3/TNFRSF1A/PIK3CB/IL18/IFNAR2/IKBKG/VDAC1/HLA-DOB/HLA-DQA1/CDK4/CXCL8/BID/IFNGR1/KPNA6/RAE1 |
| Alzheimer disease | FAS/LPL/CYBB/CAPN1/CASP3/FZD1/CASP8/TUBA1B/ADAM10/WNT10B/TUBA1C/TUBB/XBP1/NDUFC1/TNFRSF1A/PIK3CB/PPIF/MAPK8/TUBA1A/BACE1/WNT2B/FZD9/MAPK10/IRS2/UQCRQ/PLCB4/IKBKG/VDAC1/DDIT3/RTN3/NDUFB8/MME/CTNNB1/BID/COX7A1/WNT5B/COX6C/CACNA1D |
| Proteoglycans in cancer | COL1A2/FN1/LUM/FAS/CD44/TLR2/MSN/HCLS1/PRKCB/CASP3/FZD1/ITGA2/WNT10B/MAPK13/ACTB/VAV1/VEGFA/HGF/CD63/MYC/PIK3CB/MET/WNT2B/HIF1A/MMP2/PTCH1/FZD9/CAMK2G/ELK1/RPS6/HBEGF/PLCG2/CAMK2A/STAT3/RDX/CTNNB1/WNT5B |
| Human immunodeficiency virus 1 infection | FAS/CD247/APOBEC3B/MYD88/CD3D/TLR2/SAMHD1/RAC2/PRKCB/CXCR4/CASP3/TNFRSF1B/CASP8/CCNB1/GNAO1/MAPK13/CCR5/TNFRSF1A/PIK3CB/B2M/MAPK8/CCNB2/IRAK1/CHEK1/ATM/CD3G/MAPK10/HLA-B/PAK6/HLA-F/HLA-G/PAK2/IKBKG/PLCG2/PAK4/BID/HLA-C |
| Human cytomegalovirus infection | IL10RA/FAS/CCL5/CCL2/RAC2/PRKCB/CXCR4/CASP3/GNA13/CASP8/GNAO1/PDGFRA/MAPK13/CCR5/VEGFA/GNA12/JAK1/MYC/ADCY8/TNFRSF1A/PIK3CB/B2M/ADCY6/CXCL12/HLA-B/PLCB4/ELK1/HLA-F/HLA-G/IKBKG/ADCY1/STAT3/CDK4/CXCL8/CTNNB1/BID/HLA-C |
| Apoptosis | CTSS/FAS/LMNB2/GADD45B/CAPN1/CASP3/BIRC3/CASP8/NGF/TUBA1B/CASP6/TNFSF10/ACTB/TUBA1C/CFLAR/CTSK/PARP1/TNFRSF1A/PIK3CB/GADD45G/MAPK8/TUBA1A/CTSV/ATM/DFFB/GADD45A/MAPK10/BCL2A1/IKBKG/DDIT3/LMNA/CTSD/SPTAN1/BID/HTRA2/GZMB |
| Amyotrophic lateral sclerosis | CASP1/PFN1/DERL1/CASP3/TNFRSF1B/TUBA1B/GPX1/MAPK13/NUP93/ACTB/TUBA1C/TUBB/NXT1/XBP1/NDUFC1/NXF3/TNFRSF1A/NUP85/TUBA1A/GPX7/ACTR1A/NUP210/UQCRQ/NUP155/VDAC1/NUP88/SETX/DDIT3/NDUFB8/BID/DNAH9/COX7A1/GRIA1/GPX5/COX6C/RAE1 |
| Toxoplasmosis | IL10RA/ALOX5/HLA-DPA1/MYD88/LAMC2/TLR2/HLA-DMA/HLA-DRA/IFNGR2/CASP3/BIRC3/CASP8/LY96/GNAO1/MAPK13/HLA-DMB/LAMA2/HLA-DPB1/CCR5/LAMC1/JAK1/TNFRSF1A/PPIF/MAPK8/HSPA1L/IRAK1/LAMB3/MAPK10/LAMB1/IKBKG/HLA-DOB/HLA-DQA1/STAT3/CD40/IFNGR1 |
| Transcriptional misregulation in cancer | CSF1R/ITGAM/PROM1/GADD45B/CD86/BIRC3/TAF15/NR4A3/RXRA/ZBTB16/CCND2/FLT1/HPGD/SPINT1/MYC/ITGB7/GADD45G/CD14/BMP2K/BCL11B/CEBPA/MET/ATM/GADD45A/LDB1/FEV/TFE3/BCL2A1/MITF/DDIT3/IL2RB/CD40/PRCC/CXCL8/GZMB |
| Rap1 signaling pathway | ITGB2/FGF9/CSF1R/ITGAM/PFN1/ENAH/LPAR1/EGF/RAC2/PRKCB/LAT/NGF/GNAO1/PDGFRA/MAPK13/ACTB/VAV1/MAGI2/VEGFA/HGF/FLT1/ADCY8/PIK3CB/EFNA4/ADCY6/MET/FGF7/LCP2/ITGAL/PLCB4/EFNA1/ADCY1/PRKD2/CTNNB1/FGF4 |
| Chemokine signaling pathway | CXCL6/CCR2/DOCK2/CCL19/CCR7/CCL5/CCL2/RAC2/LYN/PRKCB/CXCR4/CX3CR1/ITK/NCF1/VAV1/CXCL1/CCR5/CCR6/CXCR6/PF4V1/ADCY8/PIK3CB/HCK/XCL1/ADCY6/CXCL12/PLCB4/IKBKG/ADCY1/PLCG2/STAT3/CXCL8/CCL18/CCL21 |
| Kaposi sarcoma-associated herpesvirus infection | C3/FAS/SYK/IRF9/LYN/CASP3/CD86/CASP8/LEF1/MAPK13/CXCL1/CCR5/VEGFA/JAK1/MYC/TNFRSF1A/PIK3CB/HCK/MAPK8/HIF1A/MAPK10/HLA-B/IFNAR2/HLA-F/HLA-G/IKBKG/PLCG2/STAT3/CDK4/CXCL8/CTNNB1/BID/HLA-C/IFNGR1 |
| Calcium signaling pathway | FGF9/ADRB2/EGF/PRKCB/CXCR4/NGF/P2RX4/PDGFRA/PDE1B/TNNC2/VEGFA/HGF/TACR1/GNA15/FLT1/EDNRB/ADCY8/PPIF/MET/FGF7/TBXA2R/CAMK2G/CD38/PLCB4/SPHK1/HTR2B/VDAC1/ADCY1/PLCG2/CAMK2A/SPHK2/GRM1/CACNA1D/FGF4 |
| NF-kappa B signaling pathway | LCK/CCL19/MYD88/GADD45B/SYK/LTB/BLNK/LYN/PRKCB/LAT/BIRC3/LY96/VCAM1/CXCL1/CFLAR/PARP1/TNFRSF1A/GADD45G/CD14/IRAK1/BTK/BCL10/ATM/ZAP70/CXCL12/GADD45A/MALT1/BCL2A1/IKBKG/PLCG2/CD40/CXCL8/CCL21 |
| Shigellosis | CASP1/C3/ACTN1/MYD88/CD44/PFN1/CCL5/PYCARD/ARPC1B/CAPN1/HCLS1/FOXO4/MAPK13/ACTB/TNFRSF1A/PIK3CB/CD14/MAPK8/IL18/BCL10/ATM/ARPC3/MAPK10/MALT1/PLCB4/MYL10/IKBKG/VDAC1/PLCG2/ACTR3/MYL12B/CYTH4/CXCL8 |
| Neuroactive ligand-receptor interaction | C3/GZMA/GABRP/TAC1/C3AR1/ADRA2A/P2RY14/ADRB2/LPAR1/THRA/GHR/P2RY13/CTSG/P2RX4/NMU/TACR1/EDNRB/SST/TBXA2R/EDN1/GRIK4/HTR2B/TSPO/GRIK2/NPY5R/ADM/KNG1/P2RY10/GRIA1/GLP2R/F2RL1/GRM1/ADCYAP1R1 |
| Yersinia infection | CASP1/WIPF1/FN1/LCK/MYD88/BAIAP2/CCL2/PYCARD/ARPC1B/ITGA4/RAC2/LAT/FCGR2A/MAPK13/PIP5K1C/ACTB/VAV1/RPS6KA3/CD8A/PIK3CB/MAPK8/IL18/IRAK1/RPS6KA1/LCP2/ZAP70/ARPC3/GIT2/MAPK10/IKBKG/ACTR3/CXCL8 |
| Axon guidance | ENAH/RAC2/BMP7/CXCR4/NRP1/NCK1/PLXNA2/PIK3CB/EFNA4/NTNG1/MET/SSH1/RYK/SEMA4G/PTCH1/RND1/LRIG2/CXCL12/PLXNA3/CAMK2G/SEMA6A/PAK6/EFNA1/PAK2/ABLIM1/PLCG2/CAMK2A/PAK4/MYL12B/WNT5B/EFNB1/TRPC6 |
| Ras signaling pathway | FGF9/CSF1R/PLA2G4A/EGF/RAC2/PRKCB/LAT/NGF/FOXO4/RASA2/PDGFRA/RAB5B/VEGFA/HGF/FLT1/PIK3CB/MAPK8/EFNA4/MET/FGF7/ZAP70/MAPK10/ELK1/PAK6/EFNA1/FLT3LG/PAK2/IKBKG/PLCG2/PAK4/RALGAPA1/FGF4 |
| Leukocyte transendothelial migration | ITGB2/ACTN1/ITGAM/CYBB/ITGA4/MSN/RAC2/PRKCB/CXCR4/ITK/NCF4/CLDN4/VCAM1/NCF1/MAPK13/RHOH/ACTB/VAV1/NCF2/PIK3CB/ITGAL/MMP2/OCLN/CXCL12/THY1/CLDN7/MYL10/PLCG2/MYL12B/CTNNB1/CLDN3 |
| Natural killer cell mediated cytotoxicity | CD48/ITGB2/LCK/FAS/CD247/TYROBP/SYK/FCER1G/IFNGR2/RAC2/PRKCB/LAT/CASP3/TNFSF10/VAV1/PIK3CB/SH2D1A/LCP2/ITGAL/ZAP70/HLA-B/IFNAR2/KIR2DS5/HLA-G/PLCG2/BID/HLA-C/IFNGR1/KIR2DL5A/GZMB/KIR2DL2 |
| Endocytosis | WIPF1/ARPC1B/FOLR2/CXCR4/IQSEC2/ARF5/CAPZA1/PDGFRA/PIP5K1C/EPN3/LDLRAP1/RAB5B/CCR5/CLTB/SNF8/HSPA1L/VPS37C/ARF3/ARPC3/GIT2/ZFYVE9/HLA-B/EPS15L1/HLA-F/HLA-G/ACTR3/IL2RB/ARFGAP1/CYTH4/HLA-C/DNAJC6 |
| Osteoclast differentiation | LCK/FCGR2B/CSF1R/TYROBP/SYK/BLNK/IFNGR2/IRF9/NCF4/NCF1/FCGR2A/MAPK13/NCF2/CTSK/FHL2/JAK1/TNFRSF1A/PIK3CB/MAPK8/FOSB/BTK/TNFRSF11B/LCP2/MAPK10/IFNAR2/SIRPB1/MITF/IKBKG/PLCG2/IFNGR1 |
| Prion disease | C1QB/C1QA/CYBB/C7/CCL5/RAC2/CASP3/NCF4/TUBA1B/NCF1/MAPK13/NCF2/TUBA1C/TUBB/LAMC1/NDUFC1/PIK3CB/PPIF/MAPK8/HSPA1L/TUBA1A/MAPK10/UQCRQ/VDAC1/DDIT3/NDUFB8/COX7A1/C6/COX6C/CACNA1D |
| Tight junction | CD1C/ACTN1/ARPC1B/CD1D/MSN/HCLS1/CLDN4/TUBA1B/ACTB/TUBA1C/MPDZ/DLG2/MICALL2/MAPK8/TUBA1A/PRKAB1/OCLN/ARPC3/MAPK10/CLDN7/PRKAG1/AMOT/ACTR3/CDK4/MYL12B/LLGL2/RDX/CLDN3/CACNA1D |
| Viral carcinogenesis | C3/CDC20/ACTN1/SYK/IRF9/LYN/HDAC9/CASP3/CASP8/RASA2/SNW1/YWHAH/CCR5/CCND2/JAK1/RBL1/PIK3CB/CHEK1/CCNE1/HLA-B/HLA-F/HLA-G/IKBKG/STAT3/CDK4/SCIN/HLA-C/UBR4/HNRNPK |
| Diabetic cardiomyopathy | COL1A2/CYBB/CD36/RAC2/PRKCB/NCF4/COL3A1/NCF1/MAPK13/PTEN/NCF2/PARP1/NDUFC1/MPC1/PIK3CB/PPIF/MAPK8/MMP2/CAMK2G/MAPK10/UQCRQ/PLCB4/GFPT1/VDAC1/CAMK2A/CTSD/NDUFB8/COX7A1/COX6C |
| MicroRNAs in cancer | TNC/MARCKS/EZH2/CD44/SOX4/ST14/TPM1/PRKCB/CASP3/CYP24A1/PDGFRA/PTEN/VEGFA/CCND2/DNMT1/MYC/PIK3CB/EFNA4/MET/CCNE1/ATM/IRS2/EFNA1/PLCG2/CDC25B/STAT3/PAK4/RDX/HNRNPK |
| Chagas disease | C1QB/C1QA/C3/FAS/CD247/MYD88/CD3D/TLR2/CCL5/CCL2/IFNGR2/CASP8/GNAO1/MAPK13/CFLAR/GNA15/TNFRSF1A/PIK3CB/MAPK8/IRAK1/CD3G/MAPK10/PLCB4/IKBKG/ADCY1/CXCL8/KNG1/IFNGR1 |
| Measles | FAS/FCGR2B/TLR7/MYD88/CD3D/TLR2/MSN/IRF9/SLAMF1/CASP3/CASP8/CD28/CCND2/JAK1/PIK3CB/CD209/MAPK8/HSPA1L/IRAK1/CCNE1/CD3G/MAPK10/IFNAR2/IKBKG/STAT3/IL2RB/CDK4/BID |
| Cellular senescence | GADD45B/CAPN1/TRAF3IP2/RBBP4/CCNB1/MAPK13/PTEN/HIPK2/CCND2/MYC/RBL1/PIK3CB/GADD45G/CCNB2/CHEK1/CCNE1/ATM/GADD45A/HLA-B/HLA-F/HLA-G/VDAC1/CHEK2/E2F5/CDK4/CXCL8/HLA-C/CACNA1D |
| Necroptosis | CASP1/FAS/CYBB/PLA2G4A/IL33/PYCARD/IFNGR2/IRF9/CAPN1/BIRC3/CASP8/TNFSF10/CFLAR/PARP1/JAK1/PYGL/TNFRSF1A/MAPK8/STAT4/CAMK2G/MAPK10/IFNAR2/VDAC1/PYGB/CAMK2A/STAT3/BID/IFNGR1 |
| Neutrophil extracellular trap formation | CASP1/ITGB2/C3/TLR7/ITGAM/CYBB/TLR2/SYK/VWF/RAC2/PRKCB/HDAC9/CLEC7A/NCF4/CTSG/NCF1/FCGR2A/MAPK13/ACTB/NCF2/SELPLG/PIK3CB/PPIF/ITGAL/FGG/PLCB4/VDAC1/PLCG2 |
| Leishmaniasis | ITGB2/C3/MARCKSL1/HLA-DPA1/MYD88/ITGAM/CYBB/TLR2/HLA-DMA/ITGA4/HLA-DRA/IFNGR2/PRKCB/NCF4/NCF1/FCGR2A/MAPK13/HLA-DMB/HLA-DPB1/NCF2/JAK1/EEF1A2/IRAK1/ELK1/HLA-DOB/HLA-DQA1/IFNGR1 |
| Th17 cell differentiation | LCK/CD247/HLA-DPA1/CD3D/HLA-DMA/HLA-DRA/IFNGR2/LAT/MAPK13/HLA-DMB/HLA-DPB1/NFKBIE/AHR/RXRA/JAK1/MAPK8/ZAP70/HIF1A/CD3G/MAPK10/IL21R/IKBKG/HLA-DOB/HLA-DQA1/STAT3/IL2RB/IFNGR1 |
| Cell cycle | CDC20/GADD45B/CCNB1/YWHAH/CCND2/MCM3/MCM2/MYC/RBL1/GADD45G/SFN/CCNB2/CHEK1/CCNE1/CDC14A/ATM/GADD45A/BUB1B/ANAPC13/TTK/CHEK2/CDC25B/MCM5/E2F5/CDK4/PTTG1/MCM4 |
| Wnt signaling pathway | MMP7/SERPINF1/RAC2/PRKCB/FZD1/LEF1/WNT10B/SFRP1/CXXC4/CCND2/SFRP4/CTNNBIP1/MYC/RUVBL1/MAPK8/NLK/RYK/WNT2B/FZD9/CAMK2G/MAPK10/PLCB4/GPC4/CTBP1/CAMK2A/CTNNB1/WNT5B |
| Hematopoietic cell lineage | CD1C/CD2/IL7R/CSF1R/HLA-DPA1/ITGAM/CD3D/CD44/HLA-DMA/CD36/ITGA4/HLA-DRA/CD1D/ITGA2/IL7/HLA-DMB/HLA-DPB1/CD24/CD8A/CD14/CD3G/CD38/FLT3LG/HLA-DOB/HLA-DQA1/MME |
| Viral protein interaction with cytokine and cytokine receptor | CXCL6/IL10RA/CCR2/ACKR4/CSF1R/CCL19/CCR7/CCL5/CCL2/CXCR4/CX3CR1/TNFRSF1B/TNFSF10/CXCL1/CCR5/CCR6/PF4V1/TNFRSF1A/XCL1/IL18/CXCL12/IL2RB/CXCL8/CCL18/CCL21/IL37 |
| Amoebiasis | CD1C/COL1A2/ITGB2/FN1/ACTN1/ITGAM/LAMC2/TLR2/CD1D/PRKCB/CASP3/COL3A1/CTSG/LAMA2/CXCL1/RAB5B/LAMC1/GNA15/COL4A1/PIK3CB/CD14/LAMB3/PLCB4/LAMB1/ADCY1/CXCL8 |
| T cell receptor signaling pathway | LCK/CD247/CD3D/PTPRC/LAT/ITK/NCK1/MAPK13/CD28/NFKBIE/VAV1/CD8A/PIK3CB/MAPK8/ICOS/BCL10/LCP2/ZAP70/CD3G/MAPK10/MALT1/PAK6/PAK2/IKBKG/CDK4/PAK4 |
| Alcoholic liver disease | C1QB/C1QA/C3/C3AR1/FAS/MYD88/ACACB/C2/ADH1B/CASP3/CASP8/LEF1/LY96/MAPK13/CXCL1/TNFRSF1A/CD14/MAPK8/IRAK1/PRKAB1/MAPK10/PRKAG1/IKBKG/LPIN2/CXCL8/CTNNB1 |
| NOD-like receptor signaling pathway | CASP1/MYD88/CYBB/CCL5/CCL2/PYCARD/IRF9/GBP2/BIRC3/CASP8/MAPK13/CXCL1/JAK1/MAPK8/IL18/MAPK10/PLCB4/IFNAR2/CASP5/IKBKG/VDAC1/IFI16/CARD8/PSTPIP1/CXCL8/NOD2 |
| Complement and coagulation cascades | C1QB/C1QA/ITGB2/C3/VSIG4/CLU/C3AR1/ITGAM/SERPINE2/C7/CFB/VWF/PROCR/C2/C1S/CFD/C4BPA/F11/ITGAX/FGG/SERPIND1/PROC/KNG1/C6/CFI |
| Huntington disease | SLC1A3/CASP3/CASP8/TUBA1B/GPX1/TUBA1C/TUBB/CLTB/NDUFC1/PPIF/MAPK8/TUBA1A/POLR2C/GPX7/ACTR1A/MAPK10/UQCRQ/PLCB4/VDAC1/NDUFB8/DNAH9/COX7A1/GRIA1/GPX5/COX6C |
| Viral myocarditis | ITGB2/HLA-DPA1/HLA-DMA/HLA-DRA/RAC2/CASP3/CD86/CASP8/CD28/HLA-DMB/LAMA2/HLA-DPB1/ACTB/SSPN/SNTA1/ITGAL/HLA-B/HLA-F/HLA-G/HLA-DOB/HLA-DQA1/CD40/BID/HLA-C |
| Th1 and Th2 cell differentiation | LCK/CD247/HLA-DPA1/CD3D/HLA-DMA/HLA-DRA/IFNGR2/RUNX3/LAT/MAPK13/HLA-DMB/HLA-DPB1/NFKBIE/JAK1/MAPK8/STAT4/ZAP70/CD3G/MAPK10/IKBKG/HLA-DOB/HLA-DQA1/IL2RB/IFNGR1 |
| Rheumatoid arthritis | CXCL6/ITGB2/HLA-DPA1/TLR2/HLA-DMA/CCL5/CCL2/LTB/HLA-DRA/CD86/CD28/HLA-DMB/HLA-DPB1/CXCL1/CTSK/VEGFA/FLT1/IL18/ITGAL/CXCL12/HLA-DOB/HLA-DQA1/CXCL8/ATP6V1G2 |
| Fc gamma R-mediated phagocytosis | MARCKS/MARCKSL1/FCGR2B/PLA2G4A/PTPRC/SYK/ARPC1B/RAC2/LYN/INPP5D/PRKCB/LAT/NCF1/FCGR2A/PIP5K1C/VAV1/PIK3CB/HCK/ARPC3/SPHK1/PLCG2/ACTR3/SPHK2/SCIN |
| Staphylococcus aureus infection | C1QB/C1QA/ITGB2/C3/C3AR1/FCGR2B/HLA-DPA1/ITGAM/CFB/HLA-DMA/HLA-DRA/C2/KRT19/FCGR2A/HLA-DMB/HLA-DPB1/SELPLG/C1S/CFD/ITGAL/FGG/HLA-DOB/HLA-DQA1/CFI |
| Platelet activation | COL1A2/PLA2G4A/SYK/VWF/FCER1G/LYN/GNA13/COL3A1/ITGA2/FCGR2A/MAPK13/ACTB/ADCY8/PIK3CB/ADCY6/BTK/LCP2/TBXA2R/FGG/PLCB4/PRKG1/ADCY1/PLCG2/MYL12B |
| FoxO signaling pathway | PLK2/IL7R/GADD45B/EGF/CCNB1/TNFSF10/FOXO4/MAPK13/PTEN/CCND2/PIK3CB/GADD45G/MAPK8/CCNB2/NLK/HOMER3/PRKAB1/ATM/GADD45A/MAPK10/IRS2/PRKAG1/STAT3/GRM1 |
| Hepatocellular carcinoma | GADD45B/PRKCB/FZD1/LEF1/WNT10B/SMARCC1/PTEN/ACTB/TXNRD2/HGF/MYC/PIK3CB/GADD45G/MET/WNT2B/FZD9/GADD45A/ELK1/PLCG2/CDK4/SMARCD1/CTNNB1/WNT5B/GSTA3 |
| Non-alcoholic fatty liver disease | FAS/CASP3/CASP8/MAPK13/RXRA/XBP1/NDUFC1/TNFRSF1A/PIK3CB/MAPK8/CEBPA/PRKAB1/MLX/MAPK10/IRS2/UQCRQ/PRKAG1/DDIT3/NDUFB8/CXCL8/BID/COX7A1/COX6C |
| Protein processing in endoplasmic reticulum | DERL1/PDIA4/CAPN1/DNAJB12/SSR2/STT3A/PPP1R15A/XBP1/PREB/MAPK8/HSPA1L/SSR1/UBE2J1/TRAM1/PDIA6/SEC62/MAPK10/SEC24C/DDIT3/GANAB/NSFL1C/LMAN2/SEC31A |
| Chemical carcinogenesis - reactive oxygen species | EGF/NCF1/MAPK13/PTEN/AHR/NCF2/VEGFA/HGF/NDUFC1/PIK3CB/PPIF/MAPK8/MET/HIF1A/MAPK10/UQCRQ/IKBKG/VDAC1/NDUFB8/PRKD2/COX7A1/GSTA3/COX6C |
| Pertussis | CXCL6/C1QB/CASP1/C1QA/ITGB2/C3/IRF8/MYD88/ITGAM/PYCARD/C2/CASP3/LY96/MAPK13/C1S/CD14/MAPK8/IRAK1/C4BPA/MAPK10/IRF1/CXCL8 |
| Toll-like receptor signaling pathway | TLR1/TLR7/MYD88/TLR2/CCL5/IRF9/CD86/CASP8/LY96/SPP1/MAPK13/CTSK/JAK1/PIK3CB/CD14/MAPK8/IRAK1/MAPK10/IFNAR2/IKBKG/CD40/CXCL8 |
| Phospholipase D signaling pathway | FCER1A/PLA2G4A/SYK/FCER1G/LPAR1/EGF/GNA13/PDGFRA/PIP5K1C/GNA12/ADCY8/PIK3CB/ADCY6/PLCB4/SPHK1/ADCY1/PLCG2/SPHK2/CYTH4/AGPAT3/CXCL8/GRM1 |
| Efferocytosis | C1QB/CASP1/C1QA/ALOX5/STAB1/CD36/CASP3/CX3CR1/ADAM10/HAVCR1/MAPK13/CD24/RXRA/RAB5B/HIF1A/TYRO3/CAMK2G/SPHK1/SIRPB1/CAMK2A/SPHK2/CH25H |
| Hepatitis C | FAS/EGF/IRF9/CASP3/CASP8/CLDN4/YWHAH/RXRA/CFLAR/JAK1/MYC/TNFRSF1A/PIK3CB/OCLN/IFNAR2/CLDN7/IKBKG/STAT3/CDK4/CTNNB1/BID/CLDN3 |
| Motor proteins | TPM1/KIF20A/KIF18B/TUBA1B/CAPZA1/ACTB/TNNC2/TUBA1C/TUBB/TNNI1/KIF11/MYO1F/TUBA1A/DYNLT3/ACTR1A/TNNT2/MYO5A/MYL10/MYO9A/MYL12B/DNAH9/MYO6 |
| ECM-receptor interaction | COL1A2/TNC/COL6A3/THBS2/FN1/CD44/LAMC2/CD36/VWF/ITGA4/COMP/SPP1/ITGA2/LAMA2/LAMC1/COL4A1/ITGB7/COL9A1/LAMB3/LAMB1/COL6A2 |
| Small cell lung cancer | FN1/LAMC2/GADD45B/CASP3/BIRC3/CKS1B/ITGA2/LAMA2/PTEN/RXRA/LAMC1/MYC/COL4A1/PIK3CB/GADD45G/CCNE1/LAMB3/GADD45A/LAMB1/IKBKG/CDK4 |
| AGE-RAGE signaling pathway in diabetic complications | COL1A2/FN1/CYBB/CCL2/PRKCB/CASP3/COL3A1/VCAM1/MAPK13/VEGFA/COL4A1/PIK3CB/MAPK8/EDN1/MMP2/MAPK10/PLCB4/PLCG2/STAT3/CDK4/CXCL8 |
| Insulin resistance | CD36/ACACB/PRKCB/PTEN/PTPRF/RPS6KA3/PYGL/TNFRSF1A/PIK3CB/MAPK8/RPS6KA1/PRKAB1/MLX/MAPK10/IRS2/GFPT1/PRKAG1/PPP1R3D/PYGB/STAT3/TRIB3 |
| Systemic lupus erythematosus | C1QB/C1QA/C3/ACTN1/HLA-DPA1/C7/HLA-DMA/HLA-DRA/C2/CD86/CTSG/FCGR2A/CD28/HLA-DMB/HLA-DPB1/C1S/TRIM21/HLA-DOB/HLA-DQA1/CD40/C6 |
| Gastric cancer | FGF9/GADD45B/EGF/FZD1/LEF1/WNT10B/RXRA/HGF/MYC/PIK3CB/GADD45G/MET/FGF7/CCNE1/WNT2B/FZD9/GADD45A/CDX2/CTNNB1/WNT5B/FGF4 |
| JAK-STAT signaling pathway | IL10RA/IL7R/IFNGR2/EGF/IRF9/GHR/IL7/PDGFRA/CCND2/CTF1/JAK1/MYC/PIK3CB/STAT4/IL21R/IFNAR2/STAT3/IL2RB/SOCS2/IFNGR1/SOCS7 |
| Chemical carcinogenesis - receptor activation | FGF9/ADRB2/EGF/PRKCB/AHR/RXRA/VEGFA/RPS6KA3/MYC/ADCY8/PIK3CB/ADCY6/FGF7/RPS6KA1/CYP3A4/ADCY1/STAT3/GSTA3/KPNA6/CACNA1D/FGF4 |
| cAMP signaling pathway | ADRB2/RAC2/VAV1/HCAR3/ADCY8/PIK3CB/ATP1B2/MAPK8/ADCY6/SST/GLI1/EDN1/PTCH1/CAMK2G/MAPK10/SOX9/ADCY1/CAMK2A/GRIA1/CACNA1D/ADCYAP1R1 |
| Parkinson disease | CASP3/TUBA1B/TUBA1C/TUBB/XBP1/NDUFC1/PPIF/MAPK8/TUBA1A/DUSP1/UBE2J1/CAMK2G/MAPK10/UQCRQ/VDAC1/CAMK2A/DDIT3/NDUFB8/COX7A1/HTRA2/COX6C |
| Antigen processing and presentation | CTSS/HLA-DPA1/HLA-DMA/HLA-DRA/HLA-DMB/HLA-DPB1/CD8A/B2M/HSPA1L/CD74/HLA-B/KIR2DS5/HLA-F/HLA-G/HLA-DOB/HLA-DQA1/NFYC/HLA-C/KIR2DL5A/KIR2DL2 |
| PD-L1 expression and PD-1 checkpoint pathway in cancer | LCK/CD247/MYD88/CD3D/TLR2/IFNGR2/EGF/LAT/MAPK13/CD28/PTEN/NFKBIE/JAK1/PIK3CB/ZAP70/HIF1A/CD3G/IKBKG/STAT3/IFNGR1 |
| TNF signaling pathway | CXCL6/FAS/CCL5/CCL2/CASP3/TNFRSF1B/BIRC3/CASP8/VCAM1/MAPK13/CXCL1/CFLAR/TNFRSF1A/PIK3CB/MAPK8/EDN1/MAPK10/IKBKG/IRF1/NOD2 |
| Sphingolipid signaling pathway | FCER1A/FCER1G/RAC2/PRKCB/GNA13/MAPK13/PTEN/SPTLC2/GNA12/TNFRSF1A/PIK3CB/SMPD2/MAPK8/MAPK10/PLCB4/SPHK1/CTSD/SPHK2/BID/KNG1 |
| Lysosome | LAPTM5/CTSS/M6PR/LAMP3/NEU1/CTSG/CLTB/CTSK/CD63/NPC2/LAPTM4A/CTSV/CLN3/GALC/MAN2B1/CTSD/SORT1/CTNS/AP4S1/LAPTM4B |
| Breast cancer | FGF9/GADD45B/EGF/FZD1/LEF1/WNT10B/PTEN/MYC/PIK3CB/GADD45G/FGF7/WNT2B/HEY1/NCOA3/FZD9/GADD45A/CDK4/CTNNB1/WNT5B/FGF4 |
| Hippo signaling pathway | ITGB2/BMP7/FZD1/BIRC3/LEF1/WNT10B/FRMD1/YWHAH/ACTB/TEAD3/CCND2/MYC/DLG2/BMP5/WNT2B/FZD9/AMOT/LLGL2/CTNNB1/WNT5B |
| Hepatitis B | FAS/MYD88/TLR2/PRKCB/CASP3/CASP8/MAPK13/JAK1/MYC/PIK3CB/MAPK8/IRAK1/STAT4/CCNE1/MAPK10/ELK1/IKBKG/STAT3/CXCL8/BID |
| cGMP-PKG signaling pathway | ADRA2A/ADRB2/KCNJ8/GNA13/NPR2/GNA12/KCNMB2/EDNRB/ADCY8/ATP1B2/PPIF/ADCY6/IRS2/PLCB4/PRKG1/VDAC1/ADCY1/KNG1/TRPC6/CACNA1D |
| p53 signaling pathway | RRM2/FAS/GADD45B/CASP3/CASP8/CCNB1/PTEN/CCND2/GADD45G/SFN/CCNB2/CHEK1/CCNE1/ATM/GADD45A/CHEK2/STEAP3/CDK4/BID |
| Melanogenesis | PRKCB/FZD1/TYRP1/LEF1/GNAO1/WNT10B/EDNRB/ADCY8/ADCY6/WNT2B/EDN1/FZD9/CAMK2G/PLCB4/MITF/ADCY1/CAMK2A/CTNNB1/WNT5B |
| HIF-1 signaling pathway | CYBB/IFNGR2/EGF/PRKCB/ENO3/ENO1/VEGFA/FLT1/PFKP/PIK3CB/EDN1/HIF1A/CAMK2G/RPS6/PLCG2/CAMK2A/STAT3/EGLN3/IFNGR1 |
| Fluid shear stress and atherosclerosis | CCL2/RAC2/VCAM1/NCF1/MAPK13/ACTB/NCF2/VEGFA/TNFRSF1A/PIK3CB/MAPK8/DUSP1/EDN1/MMP2/MAPK10/IKBKG/ACVR2A/CTNNB1/GSTA3 |
| Thermogenesis | SMARCC1/MAPK13/ACTB/ACSL4/RPS6KA3/NDUFC1/ADCY8/ADCY6/RPS6KA1/PRKAB1/UQCRQ/PRKG1/PRKAG1/RPS6/ADCY1/SMARCD1/NDUFB8/COX7A1/COX6C |
| Fc epsilon RI signaling pathway | FCER1A/ALOX5/ALOX5AP/PLA2G4A/SYK/FCER1G/RAC2/LYN/INPP5D/LAT/MAPK13/VAV1/PIK3CB/MAPK8/BTK/LCP2/MAPK10/PLCG2 |
| Inflammatory mediator regulation of TRP channels | PLA2G4A/PRKCB/NGF/ALOX12/MAPK13/ADCY8/PIK3CB/MAPK8/ADCY6/CAMK2G/MAPK10/PLCB4/HTR2B/ADCY1/PLCG2/CAMK2A/KNG1/F2RL1 |
| Oxytocin signaling pathway | PLA2G4A/PRKCB/NPR2/GNAO1/ACTB/CACNB2/ADCY8/ADCY6/PRKAB1/CACNA2D3/CAMK2G/CD38/PLCB4/ELK1/PRKAG1/ADCY1/CAMK2A/CACNA1D |
| Cushing syndrome | FZD1/LEF1/WNT10B/AHR/ADCY8/ADCY6/CCNE1/WNT2B/FZD9/CAMK2G/PLCB4/ADCY1/CAMK2A/CYP11A1/CDK4/CTNNB1/WNT5B/CACNA1D |
| Type I diabetes mellitus | FAS/HLA-DPA1/HLA-DMA/HLA-DRA/CD86/CD28/HLA-DMB/HLA-DPB1/GAD1/HLA-B/HLA-F/HLA-G/HLA-DOB/HLA-DQA1/CPE/HLA-C/GZMB |
| Graft-versus-host disease | FAS/HLA-DPA1/HLA-DMA/HLA-DRA/CD86/CD28/HLA-DMB/HLA-DPB1/HLA-B/HLA-F/HLA-G/HLA-DOB/HLA-DQA1/HLA-C/KIR2DL5A/GZMB/KIR2DL2 |
| Malaria | ITGB2/THBS2/KLRB1/ACKR1/MYD88/TLR2/CCL2/CD36/COMP/VCAM1/GYPC/HGF/IL18/MET/ITGAL/CD40/CXCL8 |
| C-type lectin receptor signaling pathway | CASP1/SYK/PYCARD/FCER1G/IRF9/CLEC7A/CASP8/MAPK13/PIK3CB/CD209/MAPK8/BCL10/MAPK10/MALT1/IKBKG/PLCG2/IRF1 |
| Growth hormone synthesis, secretion and action | PRKCB/GHR/MAPK13/IGFALS/ADCY8/PIK3CB/MAPK8/ADCY6/SST/MAPK10/IRS2/PLCB4/ADCY1/PLCG2/STAT3/SOCS2/CACNA1D |
| Relaxin signaling pathway | COL1A2/COL3A1/GNAO1/MAPK13/VEGFA/GNA15/EDNRB/ADCY8/COL4A1/PIK3CB/MAPK8/ADCY6/EDN1/MMP2/MAPK10/PLCB4/ADCY1 |
| Oocyte meiosis | CDC20/CCNB1/MAPK13/YWHAH/RPS6KA3/ADCY8/CCNB2/ADCY6/CCNE1/RPS6KA1/REC8/CPEB1/CAMK2G/ANAPC13/ADCY1/CAMK2A/PTTG1 |
| Signaling pathways regulating pluripotency of stem cells | FZD1/WNT10B/PCGF1/MAPK13/JAK1/MYC/PCGF3/PIK3CB/PCGF2/WNT2B/FZD9/INHBA/ACVR2A/MYF5/STAT3/CTNNB1/WNT5B |
| Allograft rejection | FAS/HLA-DPA1/HLA-DMA/HLA-DRA/CD86/CD28/HLA-DMB/HLA-DPB1/HLA-B/HLA-F/HLA-G/HLA-DOB/HLA-DQA1/CD40/HLA-C/GZMB |
| Intestinal immune network for IgA production | HLA-DPA1/HLA-DMA/ITGA4/HLA-DRA/CXCR4/CD86/CD28/HLA-DMB/HLA-DPB1/TNFRSF17/ITGB7/ICOS/CXCL12/HLA-DOB/HLA-DQA1/CD40 |
| Autoimmune thyroid disease | FAS/HLA-DPA1/HLA-DMA/HLA-DRA/CD86/CD28/HLA-DMB/HLA-DPB1/HLA-B/HLA-F/HLA-G/HLA-DOB/HLA-DQA1/CD40/HLA-C/GZMB |
| Hypertrophic cardiomyopathy | ITGA4/TPM1/ITGA2/LAMA2/ACTB/CACNB2/SSPN/SNTA1/ITGB7/PRKAB1/CACNA2D3/EDN1/TNNT2/PRKAG1/LMNA/CACNA1D |
| Protein digestion and absorption | CPA3/COL1A2/COL6A3/COL15A1/COL5A2/COL3A1/COL5A1/COL16A1/COL4A1/ATP1B2/COL9A1/COL14A1/COL8A2/COL6A2/MME/KCNJ13 |
| Dilated cardiomyopathy | ITGA4/TPM1/ITGA2/LAMA2/ACTB/CACNB2/SSPN/ADCY8/SNTA1/ITGB7/ADCY6/CACNA2D3/TNNT2/ADCY1/LMNA/CACNA1D |
| Glutamatergic synapse | PLA2G4A/SLC1A3/PRKCB/GNAO1/ADCY8/ADCY6/HOMER3/SLC1A6/GRIK4/PLCB4/SLC38A1/ADCY1/GRIK2/GRIA1/GRM1/CACNA1D |
| Neurotrophin signaling pathway | NGF/MAPK13/NFKBIE/RPS6KA3/PIK3CB/MAPK8/IRAK1/RPS6KA1/CAMK2G/MAPK10/PRDM4/PLCG2/ZNF274/ARHGDIB/CAMK2A/SORT1 |
| Insulin signaling pathway | ACACB/PTPRF/PYGL/PIK3CB/MAPK8/PRKAB1/MAPK10/IRS2/ELK1/PRKAG1/PPP1R3D/TRIP10/RPS6/PYGB/SOCS2/FLOT2 |
| Retrograde endocannabinoid signaling | GABRP/PRKCB/GNAO1/MAPK13/NDUFC1/ADCY8/MAPK8/ADCY6/MAPK10/PLCB4/RIMS1/ADCY1/NDUFB8/GRIA1/GRM1/CACNA1D |
| mTOR signaling pathway | PRKCB/FZD1/PRR5L/WNT10B/PTEN/RPS6KA3/TNFRSF1A/PIK3CB/RPS6KA1/NPRL2/WNT2B/FZD9/RPS6/LPIN2/WNT5B/ATP6V1G2 |
| Legionellosis | CASP1/ITGB2/C3/MYD88/ITGAM/TLR2/PYCARD/CASP3/CASP8/CXCL1/EEF1A2/CD14/HSPA1L/IL18/CXCL8 |
| Inflammatory bowel disease | HLA-DPA1/TLR2/HLA-DMA/HLA-DRA/IFNGR2/HLA-DMB/HLA-DPB1/IL18/STAT4/IL21R/HLA-DOB/HLA-DQA1/STAT3/IFNGR1/NOD2 |
| ErbB signaling pathway | EGF/PRKCB/NCK1/MYC/PIK3CB/MAPK8/CAMK2G/MAPK10/ELK1/PAK6/PAK2/HBEGF/PLCG2/CAMK2A/PAK4 |
| B cell receptor signaling pathway | FCGR2B/SYK/BLNK/RAC2/LYN/INPP5D/PRKCB/NFKBIE/VAV1/PIK3CB/BTK/BCL10/MALT1/IKBKG/PLCG2 |
| Gap junction | LPAR1/EGF/PRKCB/TUBA1B/PDGFRA/TUBA1C/TUBB/ADCY8/ADCY6/TUBA1A/PLCB4/PRKG1/HTR2B/ADCY1/GRM1 |
| GnRH signaling pathway | PLA2G4A/PRKCB/MAPK13/ADCY8/MAPK8/ADCY6/MMP2/CAMK2G/MAPK10/PLCB4/ELK1/HBEGF/ADCY1/CAMK2A/CACNA1D |
| Progesterone-mediated oocyte maturation | STK10/CCNB1/MAPK13/RPS6KA3/ADCY8/PIK3CB/MAPK8/CCNB2/ADCY6/RPS6KA1/CPEB1/MAPK10/ANAPC13/ADCY1/CDC25B |
| Adrenergic signaling in cardiomyocytes | ADRB2/TPM1/MAPK13/CACNB2/ADCY8/ATP1B2/ADCY6/CACNA2D3/TNNT2/CAMK2G/PLCB4/ADCY1/CAMK2A/CREM/CACNA1D |
| Adipocytokine signaling pathway | CD36/ACACB/TNFRSF1B/NFKBIE/ACSL4/RXRA/TNFRSF1A/MAPK8/PRKAB1/MAPK10/IRS2/PRKAG1/IKBKG/STAT3 |
| Epithelial cell signaling in Helicobacter pylori infection | CCL5/LYN/CASP3/ADAM10/MAPK13/CXCL1/MAPK8/MET/MAPK10/HBEGF/IKBKG/PLCG2/CXCL8/ATP6V1G2 |
| Melanoma | FGF9/GADD45B/EGF/PDGFRA/PTEN/HGF/PIK3CB/GADD45G/MET/FGF7/GADD45A/MITF/CDK4/FGF4 |
| Nucleocytoplasmic transport | THOC6/NUP93/NXT1/SAP18/NXF3/EEF1A2/NUP85/TNPO2/EIF4A3/NUP210/NUP155/NUP88/KPNA6/RAE1 |
| Serotonergic synapse | ALOX5/PLA2G4A/PRKCB/CASP3/GNAO1/ALOX12/KCNN2/DUSP1/ALOX12B/PLCB4/HTR2B/SLC6A4/ALOX15B/CACNA1D |
| Vascular smooth muscle contraction | PLA2G4A/PRKCB/GNA13/NPR2/GNA12/KCNMB2/ADCY8/ADCY6/EDN1/PLCB4/PRKG1/ADCY1/ADM/CACNA1D |
| Estrogen signaling pathway | KRT19/FKBP5/GNAO1/ADCY8/PIK3CB/HSPA1L/ADCY6/MMP2/NCOA3/PLCB4/HBEGF/ADCY1/CTSD/GRM1 |
| Pancreatic cancer | GADD45B/EGF/RAC2/VEGFA/JAK1/PIK3CB/GADD45G/MAPK8/GADD45A/MAPK10/IKBKG/STAT3/CDK4 |
| Insulin secretion | PRKCB/VAMP2/KCNMB2/ADCY8/ATP1B2/ADCY6/KCNN2/CAMK2G/PLCB4/ADCY1/CAMK2A/CACNA1D/ADCYAP1R1 |
| Arrhythmogenic right ventricular cardiomyopathy | ITGA4/LEF1/ITGA2/LAMA2/ACTB/CACNB2/SSPN/SNTA1/ITGB7/CACNA2D3/LMNA/CTNNB1/CACNA1D |
| IL-17 signaling pathway | CXCL6/CCL2/CASP3/TRAF3IP2/CASP8/MAPK13/CXCL1/LCN2/MAPK8/FOSB/MAPK10/IKBKG/CXCL8 |
| TGF-beta signaling pathway | FMOD/BMP7/FBN1/MYC/RBL1/BMP5/SMAD6/GREM1/ZFYVE9/INHBA/ACVR2A/E2F5/NBL1 |
| Parathyroid hormone synthesis, secretion and action | PRKCB/GNA13/STK39/CYP24A1/RXRA/GNA12/ADCY8/ADCY6/CYP27B1/SLC34A2/PLCB4/HBEGF/ADCY1 |
| Thyroid hormone signaling pathway | PRKCB/THRA/ACTB/RXRA/PFKP/MYC/PIK3CB/ATP1B2/HIF1A/NCOA3/PLCB4/PLCG2/CTNNB1 |
| Primary immunodeficiency | LCK/IL7R/CD3D/PTPRC/BLNK/ADA/CD8A/ICOS/BTK/ZAP70/IKBKG/CD40 |
| Cholesterol metabolism | LPL/CD36/LDLRAP1/NPC2/APOH/LRPAP1/LIPG/SOAT1/APOC3/VDAC1/TSPO/SORT1 |
| Basal cell carcinoma | GADD45B/FZD1/LEF1/WNT10B/GADD45G/GLI1/WNT2B/PTCH1/FZD9/GADD45A/CTNNB1/WNT5B |
| Non-small cell lung cancer | GADD45B/EGF/PRKCB/RXRA/HGF/PIK3CB/GADD45G/MET/GADD45A/PLCG2/STAT3/CDK4 |
| Thyroid hormone synthesis | PDIA4/PRKCB/GPX1/ADCY8/ATP1B2/ADCY6/TTF2/GPX7/PLCB4/ADCY1/SLC26A4/GPX5 |
| Glioma | GADD45B/EGF/PRKCB/PDGFRA/PTEN/PIK3CB/GADD45G/GADD45A/CAMK2G/PLCG2/CAMK2A/CDK4 |
| EGFR tyrosine kinase inhibitor resistance | EGF/PRKCB/PDGFRA/PTEN/VEGFA/HGF/JAK1/PIK3CB/MET/RPS6/PLCG2/STAT3 |
| Colorectal cancer | GADD45B/EGF/RAC2/CASP3/LEF1/MYC/PIK3CB/GADD45G/MAPK8/GADD45A/MAPK10/CTNNB1 |
| Adherens junction | ACTN1/BAIAP2/RAC2/LEF1/ACTB/PTPRF/NLK/MET/MYL10/MYL12B/CTNNB1/SNAI1 |
| Circadian entrainment | PRKCB/GNAO1/ADCY8/ADCY6/CAMK2G/PLCB4/PRKG1/ADCY1/CAMK2A/GRIA1/CACNA1D/ADCYAP1R1 |
| Salivary secretion | LYZ/ADRB2/PRKCB/VAMP2/ADCY8/ATP1B2/ADCY6/CD38/PLCB4/PRKG1/ADCY1/BST1 |
| Aldosterone synthesis and secretion | PRKCB/ADCY8/ATP1B2/ADCY6/CAMK2G/CYP11B2/PLCB4/ADCY1/CAMK2A/CYP11A1/PRKD2/CACNA1D |
| Choline metabolism in cancer | PLA2G4A/EGF/RAC2/PRKCB/PDGFRA/PIP5K1C/SLC44A4/PIK3CB/MAPK8/HIF1A/MAPK10/SLC22A3 |
| Purine metabolism | RRM2/IMPDH2/NPR2/PDE1B/ENTPD1/ADA/ADCY8/ITPA/AMPD1/ADCY6/ADCY1/ATIC |
| Spinocerebellar ataxia | PRKCB/XBP1/PIK3CB/PPIF/MAPK8/SLC1A6/MAPK10/PLCB4/KCND3/VDAC1/GRIA1/GRM1 |
| Acute myeloid leukemia | CSF1R/ITGAM/LEF1/ZBTB16/MYC/PIK3CB/CD14/CEBPA/BCL2A1/IKBKG/STAT3 |
| Renal cell carcinoma | VEGFA/HGF/PIK3CB/MET/HIF1A/PAK6/TFE3/PAK2/PAK4/EGLN3/PRCC |
| GABAergic synapse | GABRP/PRKCB/GNAO1/ADCY8/ADCY6/GAD1/SLC38A1/ADCY1/PLCL1/SLC12A5/CACNA1D |
| Phosphatidylinositol signaling system | PPIP5K1/INPP5D/PRKCB/PIP5K1C/PTEN/SYNJ2/PIK3CB/CDIPT/PLCB4/INPP4B/PLCG2 |
| Endocrine resistance | MAPK13/ADCY8/PIK3CB/MAPK8/ADCY6/MMP2/NCOA3/MAPK10/HBEGF/ADCY1/CDK4 |
| Pancreatic secretion | CPA3/CEL/PRKCB/ADCY8/ATP1B2/ADCY6/CLCA1/CD38/PLCB4/ADCY1/BST1 |
| Apelin signaling pathway | GNA13/SPP1/ADCY8/ADCY6/PRKAB1/PLCB4/SPHK1/PRKAG1/RPS6/ADCY1/SPHK2 |
| Asthma | FCER1A/HLA-DPA1/HLA-DMA/HLA-DRA/FCER1G/HLA-DMB/HLA-DPB1/HLA-DOB/HLA-DQA1/CD40 |
| Endometrial cancer | GADD45B/EGF/LEF1/PTEN/MYC/PIK3CB/GADD45G/GADD45A/ELK1/CTNNB1 |
| Long-term depression | PLA2G4A/LYN/PRKCB/GNA13/GNAO1/GNA12/PLCB4/PRKG1/GRIA1/GRM1 |
| Long-term potentiation | PRKCB/RPS6KA3/ADCY8/RPS6KA1/CAMK2G/PLCB4/ADCY1/CAMK2A/GRIA1/GRM1 |
| Platinum drug resistance | FAS/TOP2A/CASP3/BIRC3/CASP8/XPA/PIK3CB/ATM/BID/GSTA3 |
| Gastric acid secretion | PRKCB/ACTB/ADCY8/ATP1B2/ADCY6/SST/CAMK2G/PLCB4/ADCY1/CAMK2A |
| Bacterial invasion of epithelial cells | FN1/ARPC1B/HCLS1/ACTB/CLTB/PIK3CB/MET/ARPC3/ACTR3/CTNNB1 |
| Cytosolic DNA-sensing pathway | CASP1/IL33/CCL5/SAMHD1/PYCARD/CASP3/CASP8/IL18/IKBKG/IFI16 |
| Cholinergic synapse | PRKCB/GNAO1/ADCY8/PIK3CB/ADCY6/CAMK2G/PLCB4/ADCY1/CAMK2A/CACNA1D |
| Dopaminergic synapse | PRKCB/GNAO1/MAPK13/MAPK8/CAMK2G/MAPK10/PLCB4/CAMK2A/GRIA1/CACNA1D |
| Autophagy - animal | PTEN/SNAP29/CFLAR/PIK3CB/MAPK8/HIF1A/MAPK10/IRS2/SUPT20H/CTSD |
| African trypanosomiasis | FAS/MYD88/PRKCB/IDO1/VCAM1/IL18/PLCB4/KNG1/F2RL1 |
| VEGF signaling pathway | PLA2G4A/RAC2/PRKCB/MAPK13/VEGFA/PIK3CB/SPHK1/PLCG2/SPHK2 |
| Prolactin signaling pathway | MAPK13/CCND2/PIK3CB/MAPK8/MAPK10/STAT3/SOCS2/IRF1/SOCS7 |
| Inositol phosphate metabolism | INPP5D/PIP5K1C/PTEN/SYNJ2/PIK3CB/CDIPT/PLCB4/INPP4B/PLCG2 |
| Chronic myeloid leukemia | GADD45B/BCR/MYC/PIK3CB/GADD45G/GADD45A/IKBKG/CTBP1/CDK4 |
| Nucleotide metabolism | RRM2/IMPDH2/TYMP/UPP1/CDA/ENTPD1/ADA/ITPA/AMPD1 |
| Cardiac muscle contraction | TPM1/CACNB2/ATP1B2/CACNA2D3/TNNT2/UQCRQ/COX7A1/COX6C/CACNA1D |
| Prostate cancer | EGF/LEF1/PDGFRA/PTEN/SPINT1/PIK3CB/CCNE1/IKBKG/CTNNB1 |
| Glucagon signaling pathway | ACACB/PFKP/PYGL/PRKAB1/CAMK2G/PLCB4/PRKAG1/PYGB/CAMK2A |
| ATP-dependent chromatin remodeling | BAZ1A/RBBP4/SMARCC1/ACTB/TRRAP/RUVBL1/BAZ1B/SMARCD1/MORF4L2 |
| Spliceosome | SNW1/HSPA1L/EIF4A3/HNRNPU/U2AF2/LSM2/LSM3/DHX38/HNRNPK |
| Apoptosis - multiple species | CASP3/BIRC3/CASP8/TNFRSF1A/MAPK8/MAPK10/BID/HTRA2 |
| DNA replication | RPA3/LIG1/RPA2/RFC2/MCM3/MCM2/MCM5/MCM4 |
| Thyroid cancer | GADD45B/LEF1/RXRA/MYC/GADD45G/CCDC6/GADD45A/CTNNB1 |
| Bladder cancer | EGF/TYMP/VEGFA/MYC/MMP2/HBEGF/CDK4/CXCL8 |
| Arachidonic acid metabolism | ALOX5/PLA2G4A/HPGDS/GGT5/ALOX12/HPGD/ALOX12B/ALOX15B |
| Longevity regulating pathway - multiple species | ADCY8/PIK3CB/HSPA1L/ADCY6/PRKAB1/IRS2/PRKAG1/ADCY1 |
| Renin secretion | ADRB2/PDE1B/ADCY6/EDN1/CLCA1/PLCB4/CACNA1D/ADCYAP1R1 |
| Synaptic vesicle cycle | SLC1A3/VAMP2/SLC6A2/CLTB/SLC1A6/RIMS1/SLC6A4/ATP6V1G2 |
| Drug metabolism - other enzymes | RRM2/IMPDH2/TYMP/UPP1/CDA/ITPA/CYP3A4/GSTA3 |
| Polycomb repressive complex | EZH2/RBBP4/PCGF1/PCGF3/PCGF2/EZH1/RING1/CBX8 |
| Bile secretion | RXRA/ADCY8/ABCC3/ATP1B2/ADCY6/KCNN2/CYP3A4/ADCY1 |
| Mitophagy - animal | MON1B/RAB5B/MAPK8/HIF1A/MAPK10/TFE3/FKBP8/MITF |
| Ribosome | RPS3/MRPL11/RPS5/MRPL18/RPS6/RPL39/RPS19/RPS12 |
| Ovarian steroidogenesis | ALOX5/PLA2G4A/ADCY8/ADCY6/ADCY1/CYP11A1/HSD17B1 |
| Sphingolipid metabolism | NEU1/SPTLC2/SMPD2/SPHK1/GALC/SPHK2/B4GALT5 |
| Glutathione metabolism | RRM2/HPGDS/GGT5/GPX1/GPX7/GSTA3/GPX5 |
| Regulation of lipolysis in adipocytes | ADRB2/ADCY8/PIK3CB/ADCY6/IRS2/PRKG1/ADCY1 |
| Nucleotide excision repair | XPA/RPA3/LIG1/RPA2/RFC2/POLR2C/MNAT1 |
| Glycerolipid metabolism | LPL/CEL/LIPG/AKR1B1/AKR1B10/LPIN2/AGPAT3 |
| Retinol metabolism | ALDH1A3/DHRS9/ADH1B/DHRS3/ALDH1A1/CYP3A4/ALDH1A2 |
| Amphetamine addiction | PRKCB/FOSB/CAMK2G/ARC/CAMK2A/GRIA1/CACNA1D |
| Central carbon metabolism in cancer | PDGFRA/PTEN/PFKP/MYC/PIK3CB/MET/HIF1A |
| RIG-I-like receptor signaling pathway | CASP8/MAPK13/MAPK8/MAPK10/IKBKG/CXCL8/RNF125 |
| Peroxisome | ACSL4/AGPS/PEX14/PXMP4/NUDT7/PEX6/PEX26 |
| Longevity regulating pathway | ADCY8/PIK3CB/ADCY6/PRKAB1/IRS2/PRKAG1/ADCY1 |
| Morphine addiction | GABRP/PRKCB/GNAO1/PDE1B/ADCY8/ADCY6/ADCY1 |
| Glycerophospholipid metabolism | PLA2G4A/ETNPPL/LPGAT1/CDIPT/LPIN2/AGPAT3/LPCAT3 |
| AMPK signaling pathway | CD36/ACACB/PFKP/PIK3CB/PRKAB1/IRS2/PRKAG1 |
| Biosynthesis of cofactors | TDO2/IDO1/DHRS3/HMBS/MTHFD2L/HAAO/NADK |
| Steroid biosynthesis | DHCR24/CEL/CYP24A1/LBR/CYP27B1/SOAT1 |
| Mucin type O-glycan biosynthesis | GCNT3/GALNT1/GALNT2/GALNT7/ST3GAL1/B4GALT5 |
| Nicotinate and nicotinamide metabolism | SIRT4/NADK/SIRT2/CD38/BST1/SARM1 |
| Type II diabetes mellitus | PIK3CB/MAPK8/MAPK10/IRS2/SOCS2/CACNA1D |
| Endocrine and other factor-regulated calcium reabsorption | PRKCB/KLK1/CLTB/ATP1B2/ADCY6/PLCB4 |
| Cortisol synthesis and secretion | ADCY8/ADCY6/PLCB4/ADCY1/CYP11A1/CACNA1D |
| GnRH secretion | PRKCB/SPP1/PIK3CB/KCNN2/PLCB4/CACNA1D |
| RNA degradation | ENO3/ENO1/PFKP/LSM2/EDC4/LSM3 |
| Taste transduction | PDE1B/ADCY8/ADCY6/TAS2R16/PLCB4/GRM1 |
| mRNA surveillance pathway | GSPT1/NXT1/SAP18/NXF3/EIF4A3/CSTF1 |
| Oxidative phosphorylation | NDUFC1/UQCRQ/NDUFB8/COX7A1/ATP6V1G2/COX6C |
| Ubiquitin mediated proteolysis | CDC20/BIRC3/MID1/UBE2J1/ANAPC13/SAE1 |
| Olfactory transduction | PDE1B/CAMK2G/PRKG1/CAMK2A/OR1A2/OR51E2 |
| Renin-angiotensin system | CPA3/CTSG/KLK1/PREP/MME |
| Glycosylphosphatidylinositol (GPI)-anchor biosynthesis | PIGV/PIGK/MPPE1/PIGL/PGAP2 |
| Antifolate resistance | FOLR2/ALOX12/ABCC3/IKBKG/ATIC |
| Virion - Hepatitis viruses | CLDN4/CD63/OCLN/CLDN7/CLDN3 |
| Vibrio cholerae infection | PDIA4/ACTB/KDELR2/PLCG2/ATP6V1G2 |
| Carbohydrate digestion and absorption | PRKCB/PIK3CB/ATP1B2/PLCB4/CACNA1D |
| Fanconi anemia pathway | RPA3/RPA2/FANCG/FANCI/PALB2 |
| Pyrimidine metabolism | RRM2/TYMP/UPP1/CDA/ENTPD1 |
| Steroid hormone biosynthesis | CYP3A4/CYP11B2/CYP11A1/HSD17B1/HSD17B12 |
| Drug metabolism - cytochrome P450 | HPGDS/FMO3/ADH1B/CYP3A4/GSTA3 |
| Biosynthesis of amino acids | ALDH18A1/TKT/ENO3/ENO1/PFKP |
| PPAR signaling pathway | LPL/CD36/ACSL4/RXRA/APOC3 |
| Carbon metabolism | TKT/ENO3/ENO1/PFKP/OGDH |
| Ribosome biogenesis in eukaryotes | NXT1/NXF3/MDN1/RPP30/REXO2 |
| Mismatch repair | RPA3/LIG1/RPA2/RFC2 |
| Glycosphingolipid biosynthesis - lacto and neolacto series | FUT4/ST3GAL4/B3GALT2/ST3GAL6 |
| SNARE interactions in vesicular transport | VAMP2/SNAP29/STX18/STX8 |
| Aldosterone-regulated sodium reabsorption | PRKCB/PIK3CB/ATP1B2/SFN |
| Homologous recombination | RPA3/RPA2/ATM/PALB2 |
| Tryptophan metabolism | TDO2/IDO1/HAAO/AOC1 |
| Ferroptosis | CYBB/ACSL4/STEAP3/LPCAT3 |
| Base excision repair | LIG1/RFC2/PARP1/MPG |
| Vasopressin-regulated water reabsorption | VAMP2/RAB5B/ADCY6/ARHGDIB |
| Other types of O-glycan biosynthesis | GALNT1/GALNT2/POFUT2/GALNT7 |
| Amino sugar and nucleotide sugar metabolism | CYB5R4/UAP1L1/GFPT1/CYB5R3 |
| Ether lipid metabolism | PLA2G4A/PAFAH1B3/PLA2G7/AGPS |
| N-Glycan biosynthesis | STT3A/MAN2A2/GANAB/MGAT5 |
| Fatty acid metabolism | ACSL4/ELOVL6/ELOVL4/HSD17B12 |
| Mineral absorption | ATP1B2/SLC34A2/ATOX1/TRPM6 |
| Viral life cycle - HIV-1 | APOBEC3B/SAMHD1/CXCR4/CCR5 |
| Glycolysis / Gluconeogenesis | ADH1B/ENO3/ENO1/PFKP |
| Chemical carcinogenesis - DNA adducts | HPGDS/CYP3A4/GSTA3/CYP3A43 |
| Metabolism of xenobiotics by cytochrome P450 | HPGDS/ADH1B/CYP3A4/GSTA3 |
| Virion - Ebolavirus, Lyssavirus and Morbillivirus | FCGR2B/SLAMF1/CD209 |
| Taurine and hypotaurine metabolism | FMO3/GGT5/GAD1 |
| Glycosaminoglycan biosynthesis - chondroitin sulfate / dermatan sulfate | CHST15/CHST7/CHPF |
| Glycosaminoglycan biosynthesis - heparan sulfate / heparin | HS3ST1/EXT1/HS3ST3A1 |
| Fatty acid elongation | ELOVL6/ELOVL4/HSD17B12 |
| Biosynthesis of unsaturated fatty acids | ELOVL6/ELOVL4/HSD17B12 |
| Hippo signaling pathway - multiple species | RASSF2/FRMD1/TEAD3 |
| Pentose phosphate pathway | TKT/PFKP/RBKS |
| Galactose metabolism | PFKP/AKR1B1/AKR1B10 |
| Fructose and mannose metabolism | PFKP/AKR1B1/AKR1B10 |
| Circadian rhythm | BHLHE41/PRKAB1/PRKAG1 |
| Starch and sucrose metabolism | PYGL/GYG2/PYGB |
| Various types of N-glycan biosynthesis | STT3A/MAN2A2/CHST8 |
| Basal transcription factors | TAF15/TAF12/MNAT1 |
| Hedgehog signaling pathway | CCND2/GLI1/PTCH1 |
| Notch signaling pathway | SNW1/HEY1/CTBP1 |
| Lysine degradation | EZH2/AASS/EZH1 |
| Aminoacyl-tRNA biosynthesis | HARS2/CARS2/GATC |
| Alcoholism | HDAC9/GNAO1/FOSB |
| Glycosphingolipid biosynthesis - ganglio series | ST3GAL5/ST3GAL1 |
| Fatty acid biosynthesis | ACACB/ACSL4 |
| Other glycan degradation | NEU1/MAN2B1 |
| Folate biosynthesis | AKR1B1/AKR1B10 |
| Linoleic acid metabolism | PLA2G4A/CYP3A4 |
| Protein export | SEC62/SEC11A |
| Pentose and glucuronate interconversions | AKR1B1/AKR1B10 |
| Tyrosine metabolism | ADH1B/TYRP1 |
| Alanine, aspartate and glutamate metabolism | GAD1/GFPT1 |
| Biosynthesis of nucleotide sugars | UAP1L1/GFPT1 |
| One carbon pool by folate | MTHFD2L/ATIC |
| Nicotine addiction | GABRP/GRIA1 |
| Fatty acid degradation | ADH1B/ACSL4 |
| Fat digestion and absorption | CD36/CEL |
| ABC transporters | ABCC3/ABCA8 |
| Proteasome | PSMB10/PSMB8 |
| Pyruvate metabolism | ACACB/ADH1B |
| Arginine and proline metabolism | ALDH18A1/AOC1 |
| Non-homologous end-joining | XRCC5 |
| Glycosaminoglycan biosynthesis - keratan sulfate | ST3GAL1 |
| Thiamine metabolism | THTPA |
| Glycosphingolipid biosynthesis - globo and isoglobo series | ST3GAL1 |
| Primary bile acid biosynthesis | CH25H |
| Selenocompound metabolism | TXNRD2 |
| Nitrogen metabolism | CA9 |
| Lipoic acid metabolism | OGDH |
| Histidine metabolism | AOC1 |
| Mannose type O-glycan biosynthesis | FUT4 |
| Proximal tubule bicarbonate reclamation | ATP1B2 |
| alpha-Linolenic acid metabolism | PLA2G4A |
| Butanoate metabolism | GAD1 |
| Collecting duct acid secretion | ATP6V1G2 |
| Citrate cycle (TCA cycle) | OGDH |
| beta-Alanine metabolism | GAD1 |
| Propanoate metabolism | ACACB |
| 2-Oxocarboxylic acid metabolism | OGDH |
| RNA polymerase | POLR2C |
| Porphyrin metabolism | HMBS |
| Valine, leucine and isoleucine degradation | IVD |
| Cocaine addiction | FOSB |
| Cysteine and methionine metabolism | DNMT1 |
