# Supplementary material for: Gene regulatory networks involved in activation of Notch signaling by AGEs in the pathogenesis of diabetic kidney disease
Source: PLoS One. 2026 Jan 2;21(1):e0335768. doi: 10.1371/journal.pone.0335768 (PMC12758727; doi:10.1371/journal.pone.0335768)
Supplement: S1 File — S1 Table. List of up and down regulated genes from GSE 30122. S2 Table. KEGG Pathways associated with DEGs. S3 Table. Hallmarks in the dataset obtained from GSE analysis. S4 Table. Trend for logFC within the existing data and independent data from KPMP Database. (ZIP) [file pone.0335768.s001.zip › S3 Table.docx]

| Description | Set Size | Enrichment Score | NES | p.adjust |
| --- | --- | --- | --- | --- |
| HALLMARK_ALLOGRAFT_REJECTION | 75 | 0.491 | 3.271 | 0.000 |
| HALLMARK_COMPLEMENT | 42 | 0.549 | 3.086 | 0.000 |
| HALLMARK_EPITHELIAL_MESENCHYMAL_TRANSITION | 60 | 0.459 | 2.877 | 0.000 |
| HALLMARK_INTERFERON_GAMMA_RESPONSE | 61 | 0.458 | 2.902 | 0.000 |
| HALLMARK_INFLAMMATORY_RESPONSE | 54 | 0.436 | 2.659 | 0.000 |
| HALLMARK_KRAS_SIGNALING_UP | 55 | 0.433 | 2.652 | 0.000 |
| HALLMARK_APICAL_JUNCTION | 46 | 0.433 | 2.507 | 0.000 |
| HALLMARK_E2F_TARGETS | 40 | 0.387 | 2.147 | 0.004 |
| HALLMARK_ESTROGEN_RESPONSE_LATE | 37 | 0.383 | 2.093 | 0.004 |
| HALLMARK_TNFA_SIGNALING_VIA_NFKB | 53 | 0.320 | 1.925 | 0.012 |
| HALLMARK_IL2_STAT5_SIGNALING | 49 | 0.323 | 1.904 | 0.018 |
| HALLMARK_APOPTOSIS | 38 | 0.338 | 1.852 | 0.022 |
| HALLMARK_COAGULATION | 29 | 0.372 | 1.832 | 0.026 |
| HALLMARK_MYOGENESIS | 36 | -0.230 | -1.653 | 0.043 |
