# Supplementary material for: Gene regulatory networks involved in activation of Notch signaling by AGEs in the pathogenesis of diabetic kidney disease
Source: PLoS One. 2026 Jan 2;21(1):e0335768. doi: 10.1371/journal.pone.0335768 (PMC12758727; doi:10.1371/journal.pone.0335768)
Supplement: Attachment — Submitted filename: PLoS One Rebuttal Letter.docx [file pone.0335768.s003.docx]

**Response to the Reviewers:**

We appreciate the Editor and Reviewers for their time and insightful comments. We provided point-to-point responses to the reviewer’s comments, and we have revised the manuscript considering the comments from the reviewers. We acknowledge that the comments significantly improved the manuscript.

**Editor’s Comments**

**Major Comments**

**1. Both reviewers have noted the abundant availability of next-generation sequencing (NGS) datasets with greater dynamic range and sensitivity than microarrays. Please provide a concise justification for electing to analyze a microarray dataset rather than RNA-Seq or other high-throughput platforms. Importantly, the manuscript would be strengthened by an independent validation of your key findings using RNA-Seq data.**

**Ans:** The primary objective of the study was to investigate how AGEs evoke Notch signaling in glomerular podocytes and contribute to diabetic nephropathy (DN). For this purpose, we identified a microarray dataset (GSE30122) that is relevant to the pathophysiology of DN and provides an ample number of podocyte-specific samples for robust analysis. While we acknowledge that NGS platforms generally offer greater sensitivity and dynamic range, the availability of a robustly provided podocyte-specific RNA-seq dataset directly modeling AGE-mediated DN remains limited. During our search, other datasets were identified, however, those either involved different cell types, small sample sizes, or experimental conditions that did not satisfy our objective.

As suggested by the reviewers, we have analyzed the RNA Seq data of GSE299230 and found that similar trend in expression of DDIT3, GADD45A, THBS2, CCL2, and CSF1R between microarray and RNA-seq analysis. Notably, GSE299230 the experimental model involved human renal proximal tubular epithelial (HK-2) exposed to a hyperosmotic environment using 30 mM mannitol for 72 hours. Since, AGEs are formed through a slow non-enzymatic Maillard reaction between reducing sugars and free amino groups present in biological macromolecules. The conditions provided in GSE299230 do not appropriately reflect AGE mediated responses with respect to podocytes. Nevertheless, to address the reviewers concern we have included the comparative findings from GSE299230 in the supplementary file.

To further strengthen the robustness of our conclusions, we cross-validated our results with independent regional transcriptomic datasets available in the KPMP (Kidney Precision Medicine Project) database. Violin plot analysis (Figure 4D) showed the five genes (COL6A2, ITGB7, IRAK1, TNFRSF1B, and GADD45B) demonstrated consistent expression trends in both datasets, reinforcing their relevance in the context of AGE-induced DN (Figure 4E, Supplementary Table 4).

**2. Clearly articulate the novel aspects of this study in the context of existing cardiovascular transcriptomics research. How do your findings advance the field beyond currently published microarray and RNA-Seq analyses?**

**Ans:** The crux of the study is to unravel the mechanistic insights of influence of AGEs in podocytes, in the context of diabetic nephropathy. However, the effect of AGEs on cardiovascular complications is beyond the scope of this study.

**3. Update the Introduction and Discussion to include and discuss the most recent (past 2–3 years) publications relevant to transcriptomic profiling in atherosclerosis. This will ensure that your work is situated appropriately within the current state of the art.**

**Ans:** We have provided recent literature on how AGEs contribute to the micro and macrovascular complications in diabetic patients in the introduction section (references 21, 22). Since, the study focused on the influence of AGEs in eliciting reactivation of Notch signaling in the terminally differentiated podocytes, we did not cite the references related to transcriptomic profiling in atherosclerosis.

**Minor Comments:**

**1.** Please annotate the heatmap in Figure 2 with the names of the top up- and down-regulated genes for clarity.

**Ans:** We have provided the list of the top up-and down-regulated genes in supplementary table 4.

**2. To better visualize how the 167 differentially expressed genes map onto the top enriched pathways, consider adding a heatmap or chord diagram illustrating their distribution.**

**Ans:** We added a heatmap representing top 15 pathways and number of genes participating in each pathway as figure 3A and a detailed complete list has been provided in supplementary table 5.

**3. Figure 1 requires improved layout and resolution. Please enhance the graphical design (e.g., consistent fonts, clearer panel labels, higher-resolution images) to meet journal quality standards.**

**Ans:** We have enhanced the layout resolution of Figure1.

**4. The current heatmap in Figure 4B is limited in scope. I recommend displaying all samples (rather than a subset) to provide a more comprehensive view of gene expression patterns across experimental groups.**

**Ans:** We have incorporated the suggested changes in the Figure 4B of the revised manuscript.

**Reviewer 1**

**1. Authors have performed microarray data analysis for studying diabetic nephropathy and diabetic kidney disease. Given that RNA-Seq is a more advanced and comprehensive technique for transcriptomic profiling, could you please elaborate on the rationale for choosing microarrays over RNA-Seq? Was this decision influenced by data availability, platform compatibility, or any other specific reason?**

Ans: The primary objective of the study was to investigate how AGEs evoke Notch signaling in glomerular podocytes and contribute to diabetic nephropathy (DN). During our search for suitable transcriptomic datasets, we came across GSE30122, a microarray dataset, which is highly relevant to the pathophysiology of DN and provides an ample number of samples for robust analysis. Although other datasets were also identified, their limited sample sizes made them less suitable for our study.

**2. Authors have employed a combination of bioinformatics tools for functional enrichment analysis, Gene set enrichment analysis, and PPI network, which is commendable. However, I noticed that references for these tools and methods have not been provided. Could you please include appropriate citations for the tools and databases used, to ensure clarity and reproducibility for readers?**

**Ans:** In the revised manuscript, we have provided the appropriate references for the tools and databases used in the study.

**3. I noticed that some of the figure captions are quite lengthy and include detailed methodological descriptions. I would recommend revising them to be more concise and focused on explaining the figure content clearly. Methodological details can be moved to the main Methods section to improve readability and maintain consistency.**

**Ans:** In the revised manuscript we revised the figure captions for brevity and succinct.

**4. In the conclusion, the authors mention (“Our earlier experimental studies also suggest reactivation of Notch signaling is implicated in the podocytes”) that their earlier experimental studies suggest reactivation of Notch signaling in podocytes. However, I could not find any experimental validation results or supporting data presented in the current manuscript. If such validation has been performed previously, it would be helpful to include the relevant citation or briefly summarize the findings for context and clarity.**

Ans: Dear reviewer, our group has been pursuing studies with AGEs for the last ten years or so. We made several observations on cellular and molecular effects of AGEs on kidney function by performing wet-lab experiments (reference 9 (PMID: 32601154), reference 28 (PMID: 34277660), reference 6 (PMID: 25309512)). We recently shifted the focus to analyzing the transcriptomic data employing a few bioinformatics tools. Very interestingly, the outcome of bioinformatics analysis is redundant with our wet-lab studies, which we cited appropriately in the manuscript, and we highlighted in the revised manuscript.

**Reviewer 2**

**1. Although the study is well-structured and based on the GSE30122 microarray dataset, the authors do not provide an explicit justification for the exclusive use of this platform, particularly considering the availability of public RNA-Seq datasets directly related to diabetic kidney disease.**

**I recommend that the authors consult the NCBI Sequence Read Archive (SRA), which contains relevant transcriptomic data from human and murine podocytes under AGE-induced stress (e.g., SRX29078977 and SRX27070947). Given that this is an in silico study, the analysis is expected to be technically comprehensive, and a comparison with RNA-Seq data should be considered a fundamental step, either to cross-validate differentially expressed genes or to reinforce the robustness of the proposed regulatory network.**

**If such a comparison is not made, I suggest that its absence be explicitly addressed and discussed as a limitation of the study.**

**Ans:** We thank the reviewer for an excellent suggestion. The study's primary objective was to investigate how AGEs evoke Notch signaling in glomerular podocytes and contribute to diabetic nephropathy. During our search for suitable transcriptomic datasets, we came across GSE30122. This microarray dataset was highly relevant to the pathophysiology of diabetic nephropathy and provides an ample number of samples for robust analysis. Although other datasets have been identified, their limited sample sizes have made them less suitable for our study.

As suggested by the reviewers, we analyzed the RNA-seq data of GSE299230 and found a similar trend in expression of DDIT3, GADD45A, THBS2, CCL2, and CSF1R between microarray and RNA-seq analysis. Notably, GSE299230, the experimental model involved human renal proximal tubular epithelial (HK-2) cells exposed to a hyperosmotic environment using 30 mM mannitol for 72 hours. AGEs are formed through a slow non-enzymatic Maillard reaction between reducing sugars and free amino groups in biological macromolecules. The conditions provided in GSE299230 do not appropriately reflect AGE-mediated responses concerning podocytes. Nevertheless, to address the reviewers' concern, we have included the findings from GSE299230 in the supplementary file.
