# Supplementary material for: Gene regulatory networks involved in activation of Notch signaling by AGEs in the pathogenesis of diabetic kidney disease
Source: PLoS One. 2026 Jan 2;21(1):e0335768. doi: 10.1371/journal.pone.0335768 (PMC12758727; doi:10.1371/journal.pone.0335768)
Supplement: Attachment — Submitted filename: Response to Reviewers.docx [file pone.0335768.s004.docx]

**Response to Reviewers**

We appreciate the insightful comments of the Additional Editor. We provided point-to-point responses, and we have revised the manuscript accordingly.

**# The current analyses do not convincingly establish the role of the proposed core genes. Please add rigorous computational experiments.**

**Ans:** To strengthen the evidence for our proposed core genes, we performed an independent validation using the RNA-seq dataset GSE299230. RNA-seq validation description with a fully detailed workflow including data acquisition, normalization, and differential expression analysis. These analyses allow us to independently confirm the expression patterns of the proposed core genes and support their relevance in the studied condition. Sections describing this validation have been highlighted in the revised manuscript.

**# Please include RNA-seq analyses of GSE299230 in the main manuscript, with clear QC, normalization, and differential expression methods. Highlight the expression and behavior of DDIT3, GADD45A, THBS2, CCL2, and CSF1R across contrasts, and discuss how these results support (or challenge) your “core” designation.**

**Ans:** We have incorporated and highlighted the RNA-seq analyses of GSE299230 into the main manuscript.

**# If relevant RNA-seq datasets are scarce, say so plainly in the main text (not only in the supplement) and explain how this limitation affects generalizability.**

**Ans:** We have added a statement in the main text acknowledging that relevant RNA-seq datasets for this condition are limited, which may restrict the generalizability of our findings. We discuss that validation across multiple cohorts remains necessary to confirm the core gene signatures' universality fully.

**# Only 2–3 citations from 2023–2024 are included. Please update the Introduction and Discussion to reflect recent (2023–2025) work that is directly relevant to your question, and explicitly situate your findings relative to these studies.**

**Ans:** The Introduction and Discussion sections have been updated to include the most recent studies published between 2023 and 2025 (References 4, 19, 20, 21, 22, 23, 30, 31, 39, 66, 73–78).

**# Volcano plots: label the top genes (by |log2FC| and/or smallest FDR) and ensure key candidates (DDIT3, GADD45A, THBS2, CSF1R) are annotated even if they are not in the top N.**

**Ans:** Volcano plots have been updated per the suggestion and are mentioned as Fig. 5D and Fig. 5E in the revised manuscript.

**# Provide analysis code, software versions, and complete parameter settings.**

**Ans:** The analysis code can be accessed from the git hub link: <https://github.com/Somorita/-Microarray-analysis-for-AGE-responsive-core-genes-in-Diabetic-Nephropathy/tree/main>. Software versions and parameter settings are mentioned in the manuscript.

**# Limitations paragraph: explicitly address dataset scarcity, potential confounders, and the scope of inference.**

**Ans:** We have added a Limitations paragraph in the Discussion highlighting: Dataset scarcity, Potential confounders, and Scope of inference.
